# Exploring Indus crop processing: combining phytolith and macrobotanical analyses to consider the organisation of agriculture in northwest India c. 3200–1500 bc

**DOI:** 10.1007/s00334-016-0576-9

**Published:** 2016-05-21

**Authors:** Jennifer Bates, Ravindra Nath Singh, Cameron A. Petrie

**Affiliations:** 1 Division of Archaeology, University of Cambridge, Downing Street, Cambridge, CB2 3DZ UK; 2 Department of AIHC and Archaeology, Banaras Hindu University, Varanasi, 221005 India

**Keywords:** Indus Civilisation, Crop processing, Phytoliths, Plant macro-remains, South Asia, Bronze Age

## Abstract

This paper presents a preliminary study combining macrobotanical and phytolith analyses to explore crop processing at archaeological sites in Haryana and Rajasthan, northwest India. Current understanding of the agricultural strategies in use by populations associated with South Asia’s Indus Civilisation (3200–1900 bc) has been derived from a small number of systematic macrobotanical studies focusing on a small number of sites, with little use of multi-proxy analysis. In this study both phytolith and macrobotanical analyses are used to explore the organisation of crop processing at five small Indus settlements with a view to understanding the impact of urban development and decline on village agriculture. The differing preservation potential of the two proxies has allowed for greater insights into the different stages of processing represented at these sites: with macrobotanical remains allowing for more species-level specific analysis, though due to poor chaff presentation the early stages of processing were missed; however these early stages of processing were evident in the less highly resolved but better preserved phytolith remains. The combined analyses suggests that crop processing aims and organisation differed according to the season of cereal growth, contrary to current models of Indus Civilisation labour organisation that suggest change over time. The study shows that the agricultural strategies of these frequently overlooked smaller sites question the simplistic models that have traditionally been assumed for the time period, and that both multi-proxy analysis and rural settlements are deserving of further exploration.

**Electronic supplementary material:**

The online version of this article (doi:10.1007/s00334-016-0576-9) contains supplementary material, which is available to authorized users.

## Introduction

The desire to understand the socio-economic organisation of past societies has been a fundamental part of archaeology since its earliest beginnings (e.g., White 1959; Service 1962). One of the fundamental features that all models share, be they the early attempts by Childe (1950) and Service (1962) or the more nuanced and less check-list oriented approaches of Charlton and Nichols (1997), Marcus and Feinman (1998) and Yoffee (2005), is a focus on the sources of power and, most frequently, the notion of economic power. Some recent explorations of social organisation looking at more ‘complex’ societies, particularly those with multiple levels of site hierarchy, have explored the economic power relationships between urban and rural settlements with a focus on subsistence, because food is a universal requirement (e.g., Popper and Hastorf 1988; Yoffee 2005; Fuller and Stevens 2009; Weisskopf 2010; Fuller et al. 2014).

The application of crop processing models has proven to be a useful way of using food remains to study social organisation (Hillman 1981, 1984; Jones, GEM 1984; Jones, MK 1984, 1985; Weber 1991; Stevens 1996, 2003; Fuller 2002; Harvey and Fuller 2005; Fuller and Stevens 2009; Reddy 1997, 2003; Fuller et al. 2014). Traditional approaches to the assessment of crop processing have looked for ‘consumer’ and ‘producer’ sites (e.g., Hillman 1981, 1984; Jones, GEM 1984; Jones, MK 1984, 1985). Such approaches have, however, been critiqued as being overly simplistic because these terms do not simply reflect the relationships between sites and incorporate a number of assumptions, particularly in supposing that a site has a single role or relationship in the system (Khazanov 1984; van der Veen 1992; Stevens 1996, 2003; Mattingly 1997; Morley 1997; Arnold 2000; Fuller et al. 2014). More recently, models have been used which focus on when during the processing sequence crops were stored (e.g., Stevens 1996, 2003; Fuller and Stevens 2009; Fuller et al. 2014). Although other variables can influence when the crop is processed, such as a need to store the grain in chaff longer to prevent attack by pests, labour availability is a major factor in determining what stages are carried out in bulk closer to harvest and which are carried out by fewer people nearer the time of use. Harvest creates a labour bottleneck where there is a substantial amount of work to be carried out in a short amount of time if risks such as untimely rain are to be averted (Stone et al. 1990; Fuller and Stevens 2009; Fuller et al. 2014). The later stages of processing, such as the fine sieving and hand sorting, it has been argued, are more time consuming and therefore if fewer people are available to complete the large amount of work involved at harvest time, then less processing can be done before storage (Fuller et al. 2014). Provided that this holds true, then the use of a crop processing approach allows for an exploration of changes in the organisation of production and labour, from less centralised to more centralised and vice versa.

**Fig. 1:**
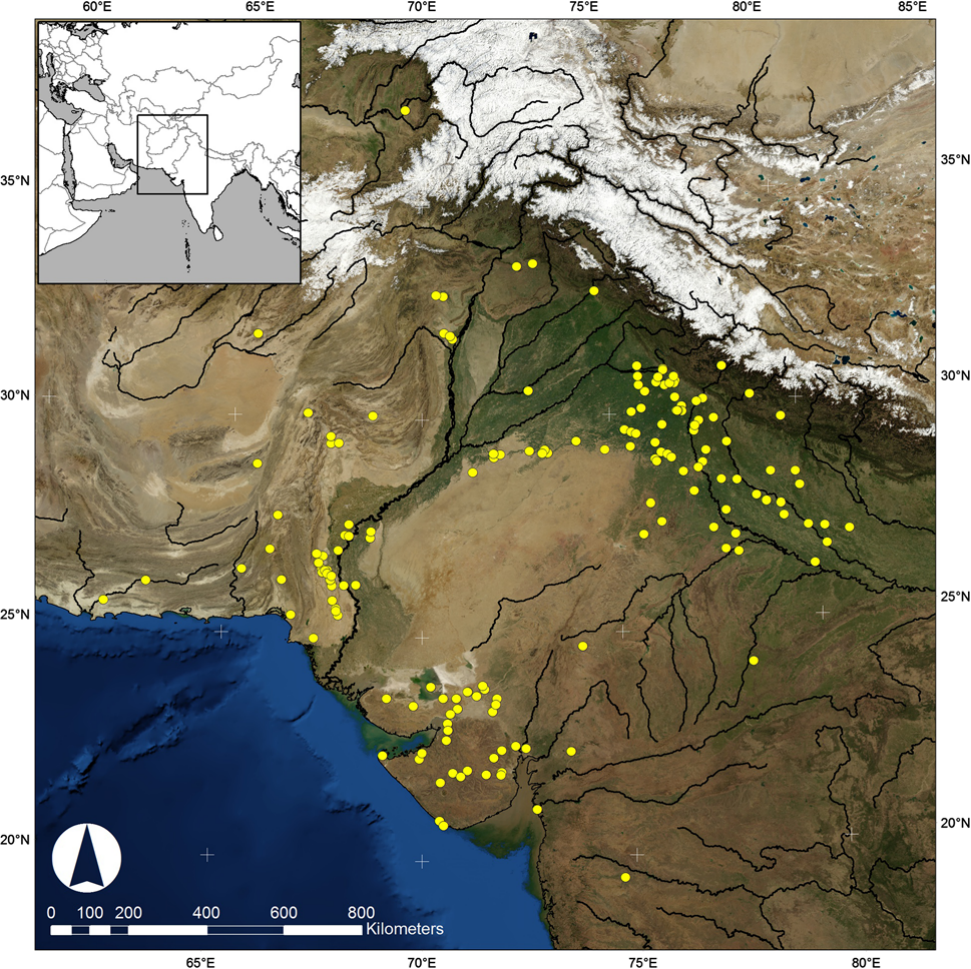
Map of the Indus Civilisation and Painted Grey Ware periods showing the distribution of excavated sites, based on published data as of publication date of paper. Based on reported excavations in Indian Archaeology, a review and Possehl (2002a)

The nature of social organisation within the Indus Civilisation (c. 3200–1300 bc) (Fig. 1; Table 1) remains one of the most debated topics of South Asian archaeology.

**Table 1:** Chronology of the Indus Civilisation (after Possehl 2002b, p. 29)

Stages	Dates (bc)	Regional phases
Early Harappan	3200–2600	Amri-Nal
		Kot Diji
		Sothi-Siswal
		Damb Sadaat
Early-Mature Harappan transition	2600–2500	
Mature Harappan	2500–1900	Sindhi Harappan
		Kulli Harappan
		Sorath Harappan
		Punjabi Harappan
		Eastern Harappan
		Quetta
		Late Kot Diji
Late Harappan	1900–1300	Jhukar (1900–1700 bc)
		Early Pirak (1800–1700 bc)
		Late Sorath Harappan (1900–1600 bc)
		Lustrous Red Ware (1600–1300 bc)
		Cemetery H (1900–1500 bc)
		Swat Valley Period IV (1650–1300 bc)
		Late Harappan in Haryana and Western Uttar Pradesh (1900–1300 bc)
Painted Grey Ware (PGW) (north-east regional development)	1300–500	Late Harappan–PGW overlap (1300–1000 bc)
		PGW (1100–500 bc)

The link between food and social organisation was noted early in Indus archaeology (e.g., Marshall 1931, p. 27). However, where this food originated was not initially theorised by early archaeologists, and this lack of discussion has continued into many subsequent models of Indus Civilisation social organisation (e.g., Wheeler 1950; Piggott 1950; Jacobsen 1987; Malik 1967, 1979; Chakrabarti 1988, 1999; Kenoyer 1997, 2000). Wright (2010) has attempted to draw on direct archaeobotanical evidence to explore urban–rural subsistence relationships in her discussion of Indus Civilisation social organisation. Based on the archaeobotanical data from Harappa, Wright (2010, pp. 166–170, 203–207) has argued that cities relied on networks of smaller settlements, with control diminishing by distance, and with reciprocal relationships existing between settlements (see Weber 2003). Extrapolating from Harappa, Wright (2010) suggested that during the urbanised Mature Harappan period (c. 2600–1900 bc, Table 1) agricultural production became centralised, with crop processing being carried out in bulk and not by households, while in the de-urbanised Late Harappan period (c. 1900–1300 bc) a shift back to decentralised processing within households was noted (Wright 2010, pp. 166–170, 203–207). Building from this, it was argued that the city of Harappa increased its control over villages in the hinterland, drawing on them for food and changing their production systems to produce a surplus to support the non-agricultural urban specialists during the Mature Harappan period (Wright 2010, pp. 166–170, 203–207).

However, Harappa is only a single city, and is located in the west of the Indus region, and Wright (2010, p. 127) acknowledged that it is unlikely to be representative of the Indus Civilisation as a whole. Similarly, a focus on cities, which are only one element of the urban–rural network, provides only half the story (Charlton and Nichols 1997, p. 9). The ‘village’, a term used here to refer to small settlements primarily involved in agricultural production (Petrie 2013; Eltsov 2008, for terminology debates), has often been neglected in Indus archaeology (Schuldenrein 2002; Mehta 1993, p. 168; Ratnagar 1991) because both the sites and their products are often less visible or durable at the village level than in the larger urban settlements. This paper will attempt to address some of this imbalance by exploring the organisation of crop production at five small ‘village’ settlements in the north-east of the Indus Civilisation excavated by the *Land*, *Water*, *Settlement Project*, thus providing a starting point for building in this missing but crucial building block in our understanding of Indus social organisation, the basic food producer.

A multi-proxy approach was undertaken as part of the bioarchaeological sampling programme for investigating plant exploitation. This approach was chosen for several reasons. While charred macrobotanical remains can provide species level identifications and are the most commonly recovered remains from Indus Civilisation sites, they require specific preservation conditions such as the chance for carbonisation (Jones and Colledge 2005) and their preservation on South Asian sites has been characterised as generally poor (Fuller 2000; Harvey 2006), possibly due to the presence of salts and other minerals in most South Asian soils. Phytoliths are siliceous “mineral deposits that form in and between plant cells” (Mulholland and Rapp 1992, p. 1), and are considerably more robust than charred plant remains (Harvey and Fuller 2005) as their silica structure is resistant to decay and can survive in a range of soil conditions. However, unlike macrobotanical remains, phytoliths are not always identifiable to a lower taxonomic level such as species or genus (Piperno 1988, 2006; Pearsall 1989; Ball et al. 1999, 2001), although recent work is beginning to demonstrate that in some cases genus and even species identifications may be possible through morphometrics (Ball et al. 2015 for summary and discussion). Phytoliths are no longer seen as the last resort of archaeobotanists in the absence of macrobotanical remains (Pearsall 1989), but as complementary data, capable of providing a different scale of information. Phytoliths are produced in a range of plant organs that are usually destroyed by fire such as the leaf or stem, thus providing a picture that would be unlikely to be preserved in macrobotanical form. A combined macrobotanical and phytolith analysis thus overcomes the different strengths and weaknesses of the two proxies. Both macrobotanical and phytolith samples from the *Land*, *Water*, *Settlement Project* excavations form the basis for this analysis. This paper uses these two proxies to explore the archaeobotanical remains at these five village settlements in order to consider the nature of labour organisation over time for crop processing, with a view to using this to think about Indus social organisation from the perspective of the villager. The combination of the two datasets provides the potential for a more robust picture of the relationship between villages and social change.

## Materials and Methods

The samples that have been analysed come from excavations in north-west India. This collaborative project, between the University of Cambridge and Banaras Hindu University with support and permissions from the Archaeological Survey of India, has been conducting surveys and excavations in the modern states of Rajasthan, Haryana and Uttar Pradesh since 2008 (Singh et al. 2008, 2010a, b, 2011, 2012, 2013a, b; Petrie et al. 2009, in press a, b, c; Pawar 2012; Dixit et al. 2014, in press; Bates 2016; Bates et al. in press; Jones, PJ et al. in press; Parikh and Petrie in press).

**Fig. 2:**
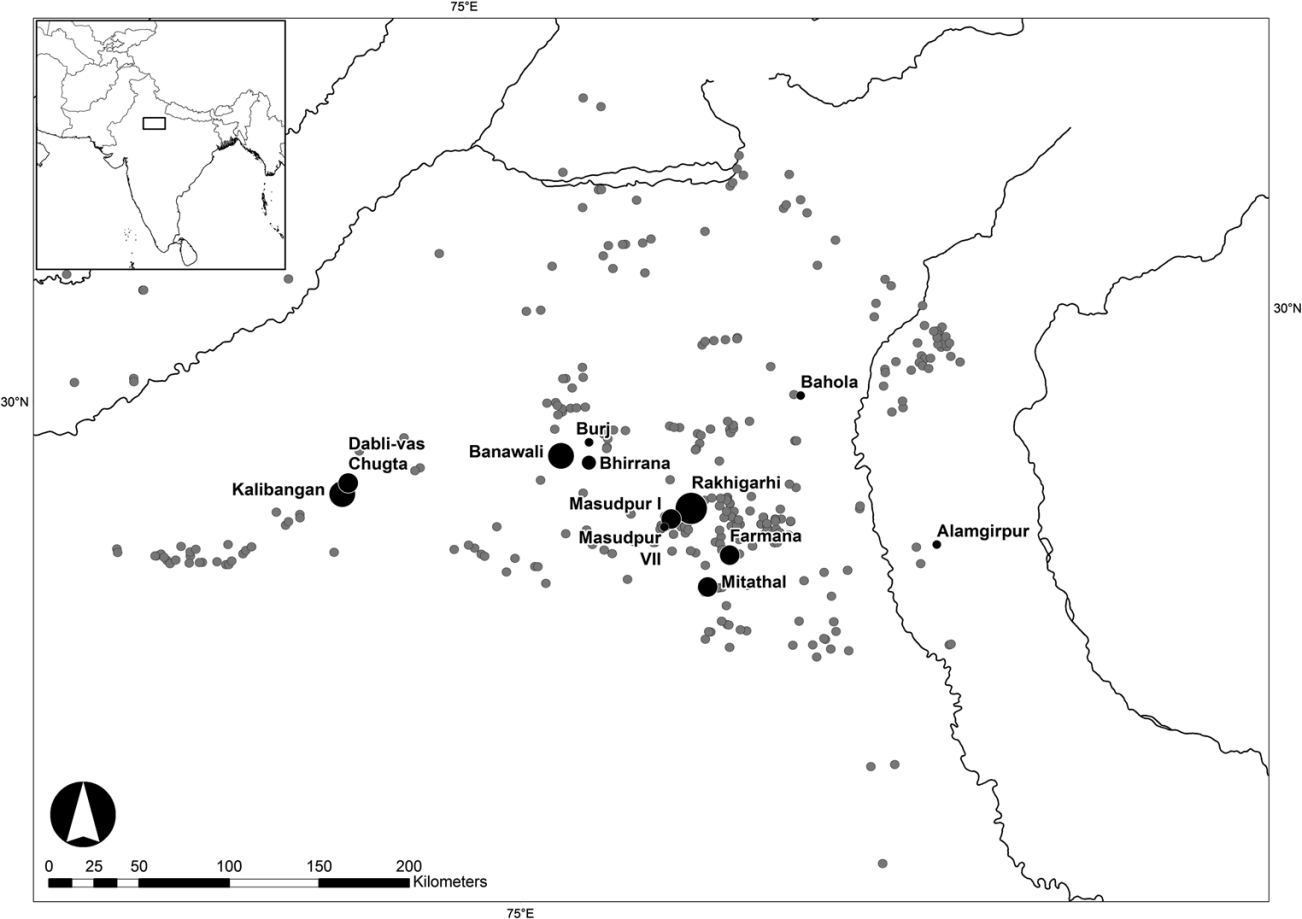
Dabli vas Chugta, Burj, Masudpur *VII* and *I*, Bahola, and Alamgirpur, six sites excavated by the Land, Water, Settlement Project and other Indus sites

Five of the settlements that have been excavated by the *Land*, *Water*, *Settlement Project* are considered here: Dabli vas Chugta, Burj, Masudpur VII, Masudpur I and Bahola (Fig. 2), which each have various phases of occupation ranging from the Early Harappan to the Painted Grey Ware (PGW) period (Table 2).

**Table 2:** Periodisation of the sites analysed in this paper

	Dabli vas Chugta (DVC)	Burj (BRJ)	Masudpur I (MSD I)	Masudpur VII (MSD VII)	Bahola (BHA)
Early Harappan	X	X		X	
Mature Harappan			X	X	
Late Harappan				X	X
PGW		X			X

Dabli vas Chugta, the most westerly settlement site, covers an area of approximately 5–6 ha (Singh et al. 2012) and is situated within 7 km of the Indus Centre of Kalibangan (Singh et al. 2012) in Hanumangarh District, Rajasthan. Extensive agricultural damage has occurred on the site, including levelling of all material above 180.9 m a.s.l., and despite the presence of Mature Harappan ceramics on the surface survey, only Early Harappan layers were found in excavations (Singh et al. 2012). Two trenches were excavated—ZA6 and ZI7 (ESM 1, Fig. S1), although only samples from ZI7 were analysed, due to poor taphonomic conditions in ZA6. Trench ZI7 was located on the east side of the site after cleaning of an exposed section revealed structural deposits. An 11 × 2 m trench was excavated revealing 60 stratigraphic deposits in three phases. These included surfaces with post-holes, mudbrick walls with associated surfaces, fills, hearths and pits, and mudbrick platforms (Singh et al. 2012) (ESM 1, Fig. S2). 38 Macrobotanical and 36 phytolith samples were taken for analysis (ESM 1, Table S1). Radiocarbon dating is pending, but the ceramics suggest that Early Harappan occupation was exposed (Singh et al. 2012).

The ancient village at Burj is located partly under its modern namesake in the modern district of Fatehabad, Haryana (Kumar 1984; Singh et al. 2010a). It lies in close proximity to the Indus settlements at Banawali, Kunal and Bhiranna. Two trenches were excavated in 2010: ZA2 and ZG9 (ESM 1, Fig. S3). Trench ZA2, a 2 × 2 m sondage, was located at the highest point of the mound in the hope of recovering the full occupation sequence. 63 Stratigraphic deposits were found in four phases, with the lowest being formed of ploughed material containing redeposited Early Harappan ceramics. This was overlain by PGW deposits that did not contain structures but did include several pits (Singh et al. 2010b) (ESM 1, Fig. S4). A second 2 × 2 m sondage, Trench ZG9, was located the northeast of ZA2 and contained 26 deposits in two phases: lower Early Harappan levels with large structures and associated fills, with several large pits cut into it, overlain by PGW rammed surfaces and pits (Singh et al. 2010b) (ESM 1, Fig. S5). A range of artefacts, including grinding stones, were found alongside both Early Harappan and PGW pottery and later Early Historic remains. 20 macrobotanical and 19 phytolith samples were recovered and analysed (ESM 1, Table S2). Radiocarbon dating has confirmed the ceramic chronology (Singh et al. 2010b; Bates 2016). The preservation of the Early Harappan macrobotanical remains was too poor for their data to be used in this analysis (Bates 2016).

Masudpur VII (known locally as Bhimwada Jodha) is a 1 ha ‘small village’ site situated within 15 km of the Indus City of Rakhigarhi in Hissar District, Haryana (Petrie et al. 2009, p. 45). Two trenches were excavated, YA2 and YB1 (ESM 1, Fig. S6). Trench YA2 was located at the highest point of the mound to identify the full cultural sequence in a 2 × 2 m sondage, while YB1 was positioned to the side of this trench and opened up as a larger trench to uncover more of the site (Petrie et al. 2009). 31 Stratigraphic layers in 9 phases were excavated from YA2 and 28 deposits in 12 phases from YB1 (ESM 1, Figs. S7, S8). In both trenches mudbrick architecture with associated occupation surfaces, fills and pits were found, along with a range of local and non-local small artefacts, including one gold and one lapis lazuli bead (Petrie et al. 2009). 25 macrobotanical and 19 phytolith samples were recovered and analysed (ESM 1, Table S3). Radiocarbon dating and the associated ceramic material suggested that this site was established in the Early Harappan period, occupied during the earlier parts of the Mature Harappan, and was reoccupied during the Late Harappan period (Petrie et al. 2009, in press a; Bates 2016).

Masudpur I (known locally as Sampolia Khera) is a 6 ha ‘large village’ site, situated around 12 km from Rakhigarhi (Petrie et al. 2009, p. 39). Three trenches were excavated—XA1, YA3, XM2 (ESM 1, Fig. S9). Trench YA3 did not produce archaeobotanical remains so this will not be discussed further. Trench XM2 was excavated because mudbrick architecture was seen in an exposed section on the western side of the mound (Petrie et al. 2009). 24 deposits in 10 phases were identified. Trench XA1 was located at the highest point of the mound in order to recover the full stratigraphic sequence. 38 deposits in 9 phases were identified. In both 2 × 2 m trenches structural deposits with associated occupation fills, surfaces and pits were identified (ESM 1, Figs. S10, S11), and a wide range of cultural material recovered, including several beads made of non-local materials like carnelian and faience (Petrie et al. 2009, in press b, c). In total 29 macrobotanical and 20 phytolith samples were analysed for this research (ESM 1, Table S4). Radiocarbon dates and the associated ceramic material indicate that the site was occupied in the middle and later parts of the Mature Harappan period (Petrie et al. 2009; Bates 2016).

Bahola is a 1–2 ha “small village” site in Karnal district with Late Harappan, PGW and Early Historic occupation (Singh et al. 2013a, p. 7; Petrie et al. in press b, c). One sounding trench, AB1 (ESM 1, Fig. S12), and a section cleaning, YK3, were excavated, but only material from AB1 was collected for analysis. Trench AB1 was located at the highest point of the mound and formed a 2 × 2 m sondage to identify the full stratigraphic sequence (Singh et al. 2013a, b). 45 deposits in 12 phases were identified, formed of structural deposits with associated fills, surfaces, pits and hearths (ESM 1, Fig. S1). As at Masudpur I and VII, local and non-local artefacts were found including agate and faience objects (Singh et al. 2013a, b). 30 macrobotanical and 30 phytolith samples were analysed for this research (ESM 1, Table S5). Radiocarbon dating is pending, although the ceramic sequence suggests occupation dating to the Late Harappan and PGW periods (Singh et al. 2013a, b; see Bates 2016).

Phytoliths were extracted from 5 g soil samples using sodium polytungstate after removal of carbonates, clays and organics following Madella et al. (1998). Phytolith preparations were counted and identified on with a GX ML3230 microscope at ×600 magnification at the George Pitt Rivers Laboratory at the McDonald Institute for Archaeology, University of Cambridge. Description of the phytoliths follows the International Code for Phytolith Nomenclature 1.0 (Madella et al. 2005). Following Lancelotti (2010), 350 individual phytoliths were counted per slide with silica skeletons counted separately, and a note of the number of phytoliths in each silica skeleton also made. A qualitative scan of each slide at ×200 magnification was also made to check for rare morphotypes and silica skeletons. Quantification of the concentration of phytoliths per gram acid insoluble fraction follows Albert and Weiner (2001).

Macrobotanical remains were obtained using a bucket flotation system. This system was used for practical reasons as availability of water and electricity was limited at the sites and materials for this system could easily be obtained in India. An average of 20 litres of sediment was aimed for, although in some cases this was not possible, such as when the context was small, e.g.: pit linings. A 500 µm mesh was used in order to maximise the range of seeds and chaff collected while preventing clogging of the mesh. The samples were sorted using a Leica MZ8 microscope in the George Pitt Rivers Laboratory at the McDonald Institute for Archaeology, University of Cambridge using their reference collection as well as that of the Institute for Archaeology, University College London and botanical literature, including Martin and Barkley (1961), Berggren (1969), Gallinato et al. (1999), Fuller (2000), Jacomet (2006), Nesbitt (2006), Cappers et al. (2009) and Zohary et al. (2012). Unfortunately there is no coherent, complete recent flora for South Asia. The *Flora of British India* by Hooker (1875) and the incomplete *Fascicles of the Flora of India* by the Botanical Survey of India do not provide coverage for the region in the detail needed and as such it was decided that the *Tropicos* online flora of Pakistan (www.tropicos.org/Project/Pakistan) would be used to describe and name the seeds as the research area borders Pakistan and the eflora makes distinctions as to which states the plants are found in, allowing for more refined comparisons with the research area. Cereal crops, however, were named according to the nomenclature in Zohary et al. (2012).

The samples recovered from these sites by flotation yielded four main types of cereals (Bates 2016): *Triticum* sp. (wheat) which, when identifiable to species level was all *T. durum*/*aestivum*, *Hordeum vulgare* (hulled barley), *Oryza* sp. (rice), and *Echinochloa colona*, *Setaria* cf. *pumila* and *Panicum* sp. (small native hulled millets). Wheat and barley are described in South Asian archaeobotany as *rabi* cereals, referring to the season in which they are sown and harvested and the rainfall they rely on, the winter rains. Rice and millets on the other hand are *kharif* cereals, sown and harvested in the summer and relying on summer monsoonal rainfall. Specific varieties of wheat and barley are common at the Indus settlements. The diversity of taxa found at the sites necessitated the use of a combination of crop processing models relevant for the different crops. For free-threshing wheat and hulled barley the approaches outlined by Hillman (1981, 1984) and Stevens (2003) were applied, for hulled millets the approaches of Reddy (1994, 1997, 2003), Harvey (2006), Harvey and Fuller (2005) and Weisskopf (2010) were used, and for rice, the approaches of Thompson (1996), Harvey (2006) and Weisskopf (2010) were used. The details of each model are outlined in ESM 2.

Several methods exist for studying crop processing. A common macrobotanical method is to look at the ratio of glumes–grains (Stevens 1996, 2003). This approach, however, relies on good preservation, which is not common on many South Asian sites (Harvey 2006). Instead simpler models looking for generalised patterns are best used. The ratios of grain to weeds to chaff for each context provide a starting point for considering whether the samples are biased towards any one element (Stevens 1996, 2003), and specific ratios can then be explored. These include weed to grain ratios, weed to size ratios (for wheat and barley) and weed weight ratios (for rice and millets) (Reddy 1994, 1997, 2003; Stevens 1996, 2003; Fuller et al. 2014). Not many models have been proposed for using phytoliths to studying crop processing. It has, however, been shown that ratios of crop husk to weed husk to leaf or culm (stem) can be useful for exploring crop processing through phytoliths (Harvey and Fuller 2005; Harvey 2006; Weisskopf 2010), and triplots have been created to look at this ratio. How the different morphotypes were classified as these elements can be found in Table 3.

**Table 3:** Classification of phytoliths used in this analysis into groups for crop processing models

Context	Classification to groups
Elongate psilate	Leaf/stem
Elongate irregular	Leaf/stem
Elongate echinate indet.	Unknown inflorescence (not included in analysis)
Echinate wheat/barley type	Wheat/barley inflorescence
Elongate dendritic wheat/barley type	Wheat/barley inflorescence
Elongate echinate short spine	Weedy type inflorescence (wheat/barley, millet and rice)
Elongate echinate hook spine	Weedy type inflorescence (wheat/barley, millet and rice)
Elongate dendritic short spine	Weedy type inflorescence (wheat/barley, millet and rice)
Elongate echinate wavy millet type indet.	Millet inflorescence
Elongate echinate wavy *Echinochloa*-type	Millet inflorescence
Elongate echinate wavy *Brachiaria*-type	Millet inflorescence
Elongate echinate wavy *Pennisetum*-type	Millet inflorescence
Elongate echinate wavy cf. *Sorghum*-type	Millet inflorescence
Elongate echinate wavy cf. *Setaria verticillata*-type	Millet inflorescence
Elongate echinate wavy cf. *Setaria italica*-type	Millet inflorescence
Elongate echinate wavy *Panicum miliaceum*-type	Millet inflorescence
Elongate non-psilate	Leaf/stem
Double peak husk	Rice inflorescence
Commelinaceae (Eichhorn^a^ L)	Commelinaceae: weedy (rice)
Commelinaceae (Eichhorn^a^ K)	Commelinaceae: weedy (rice)
Commelinaceae (Eichhorn^a^ A)	Commelinaceae: weedy (rice)
Commelinaceae (Eichhorn^a^ I)	Commelinaceae: weedy (rice)
Commelinaceae indet. cone	Commelinaceae: weedy (rice)

Only those forms included in the analysis have been outlined here. For full dataset see Bates (2016). Grass short cells were not included as these are commonly found in both inflorescence and leaf/stem and therefore cannot be used to refine the crop processing models

^a^Eichhorn et al. (2010)

It is also essential to consider taphonomy, that is, the likelihood that specific plant parts ended up at a settlement, and for macrobotanical remains, the likelihood that they would have been burnt. For example, the later stages of processing are more likely to be represented as these typically occur within settlements. Other taphonomic factors include the fact that hulled millets have a greater chance of charring than other cereals because of the need for parching, and that different plant parts also have different preservation characteristics. Lemmas and paleas, for example, are more fragile than glumes. Consideration of such factors emphasises the importance of using the combination of macrobotanical and phytolith analyses (Harvey and Fuller 2005).

## Results

The raw data on which the following discussion is based can be found in ESM 3 Tables S1, S2, S3, S4, S5, S6, S7, S8, S9 and S10 for macrobotanical remains and Tables S11, S12, S13, S14 and S15 for phytoliths; images of phytoliths are given in ESM 3, Figs. S1, S2, S3, S4, S5, S6, S7, S8, S9, S10, S11, S12, S13 and S14.

### *Rabi* (winter) crops

#### Wheat/barley macrobotanical and microbotanical remains

**Fig. 3:**
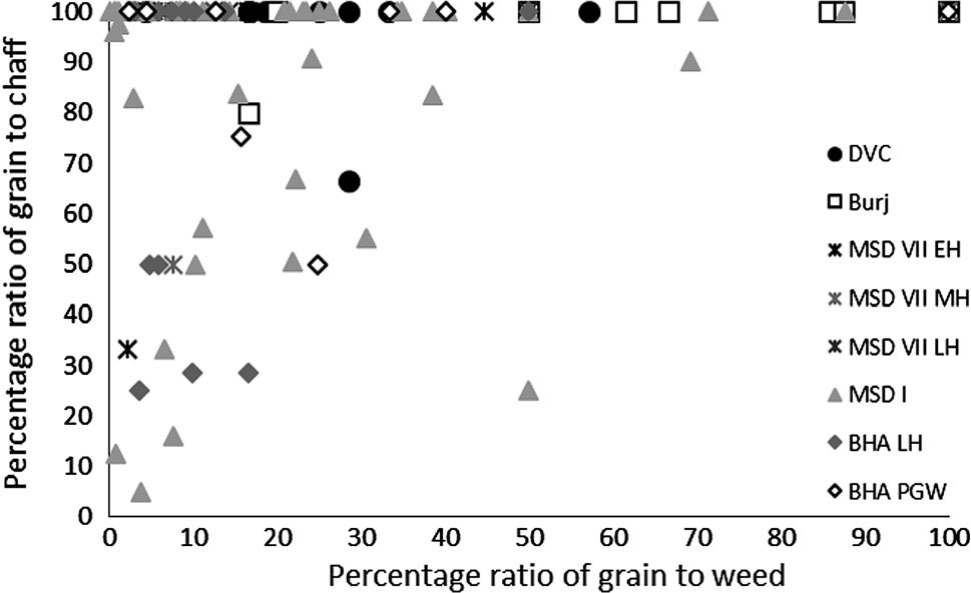
Ratio of *rabi* (winter) grain–chaff, and *rabi* (winter) grain–weed macro-remains

There were limited quantities of winter cereal chaff in the macrobotanical samples, from either wheat or barley, and with the exception of single piece of barley rachis internode from Bahola in the Late Harappan period, that which could be identified was poorly preserved and described as *Hordeum*/*Triticum* rachis internodes, based on the presence of a few features commonly noted on barley rachis internodes. As such, the analysis of the winter cereals has been combined. The low proportion of chaff compared with the significant quantities winter-grown cereal grain (Fig. 3) could be interpreted in several ways.

**Fig. 4:**
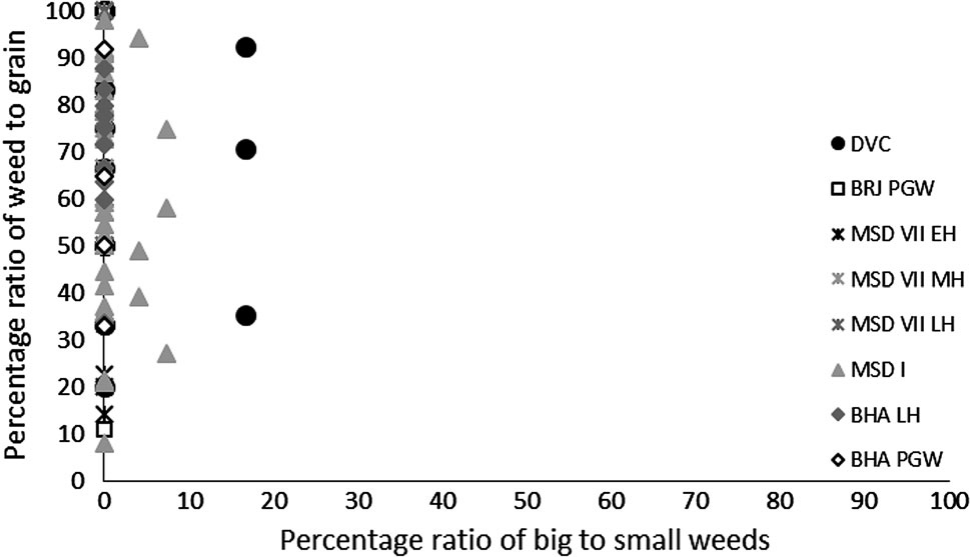
Ratio of weed seeds–*rabi* (winter) grain, and large–small weed macro-remains

These results might suggest that the grain was being stored after hummeling (pounding to remove awn bases of hulled barley: Hillman 1981, 1984) and winnowing, not in spikelet form in the case of barley, or after coarse sieving in the case of free-threshing wheat, thus implying that bulk processing of winter cereals was carried out before storage. The low proportion of chaff relative to grain could therefore be interpreted as evidence of storage before the coarse-sieving stage for hulled barley and storage after coarse sieving for free-threshing wheat (Stevens 1996, 2003). However, hummeling and winnowing in particular might also have been carried out off-site in bulk throughout the year, therefore reducing the chance of remains being burnt and/or deposited in the settlement area which was excavated. This explanation seems more likely, given that winnowing requires large open areas and is usually carried out in bulk rather than piecemeal at settlements. Coarse sieving is more likely to have been carried out on site throughout the year, probably in non-bulk quantities as it is more labour and time intensive (Stevens 1996, 2003) and therefore the lack of evidence for this on site seems unusual. Chaff is problematic taphonomically as it is more easily damaged than grain (Boardman and Jones 1990), and an alternative explanation for the lack of chaff could be its destruction due to differential reactions to fire. The only exception to the low proportion of chaff in *rabi* (winter) cereals was at Bahola in the Late Harappan period, in which five contexts had more chaff than grain, perhaps suggestive of earlier processing stages like coarse sieving having been carried out within the settlement. However, the majority of Late Harappan contexts at Bahola were rich in grain or weeds, again indicating either cooking accidents or that later stage processing were the norm. The proportion of weeds to grain was otherwise variable within sites and periods (Fig. 4), ranging from no weeds, which might indicate grains preserved in cooking accidents or the differential preservation of grains over weeds, to weed rich, which could indicate the burning of crop processing waste or these could have resulted from other processes such particular care being taken so as not to waste grain (Jones, MK 1984 , 1985).

The possibility that the *rabi* (winter) weeds were being stored after some bulk and even some non-bulk processing such as coarse sieving had occurred is further supported by the types of weeds noted. There was a lack of seed heads or seed headed taxa of winter weeds at all sites and in all periods. These data could again reflect taphonomic processes that resulted in the loss of the fragile seed heads through burning. If this was the case, some seed remains might be expected, as they are tougher than the seed heads themselves. However, no seed remains of such plants were found, supporting the idea that coarse sieving was not regularly being carried out at the site, as weeds with seed heads are removed during this stage of processing. Large seeded weeds, those of a similar size to the grain, were rare across all sites and periods. Indeed no large weed seeds were noted at Burj in the PGW period, Masudpur VII in the Mature Harappan period, and Bahola in both the Late Harappan and PGW periods. Weed seeds that are smaller than the grains dominated. The low presence or complete absence of large weed seeds in the samples could be explained in several ways: firstly that there was little hand sorting on site, which seems unlikely given the presence of grain; secondly that it is an artefact of differential preservation, again unlikely given the weed seeds are similar in size to the grain and therefore not likely to be more fragile; thirdly that this stage of processing was not being regularly carried out on site, again unlikely given the amount of fine sieving waste; or fourthly that hand sorting was either being carried out elsewhere on site, or that the waste did not reach fire to be charred. The latter explanation seems to be the most probably, and it is therefore suggested that what is seen at the sites is the result of regular daily processing of winter cereals on site from the fine sieving stage onwards. The macrobotanical remains thus suggest that winter cereal crops appear to have been stored from at least the winnowing stage, if not the coarse sieving stage onwards, and that processing waste from fine sieving was regularly burnt on site.

**Fig. 5:**
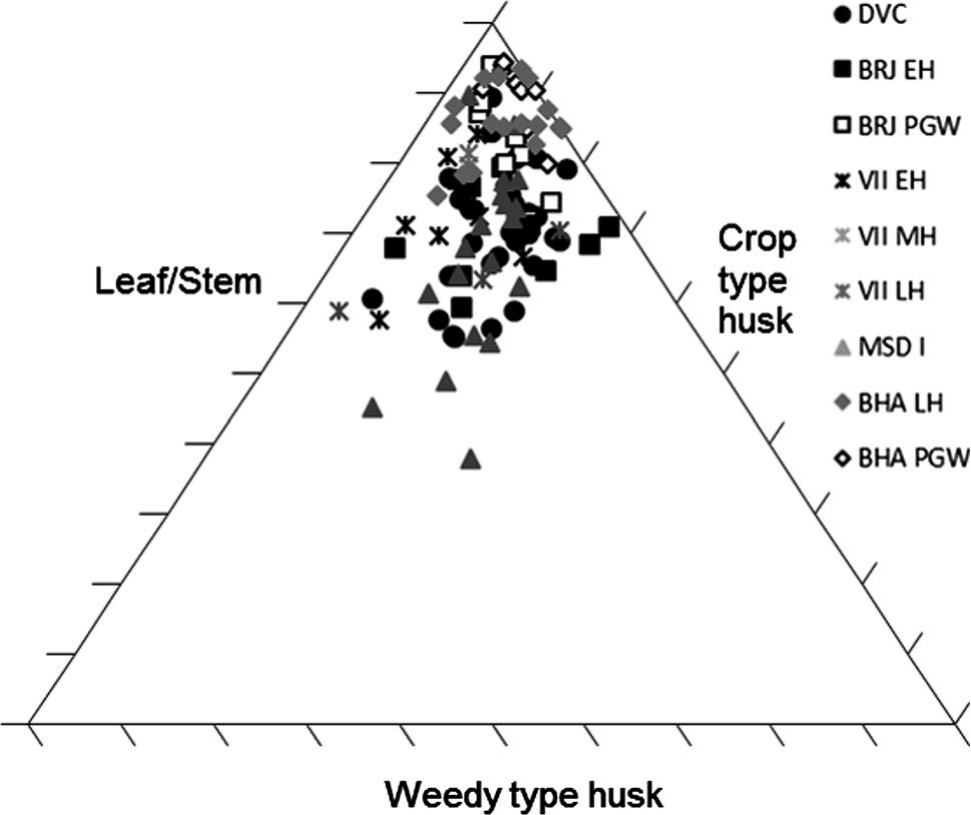
Triplot of leaf/stem, wheat/barley type husk and weedy type husk phytoliths

The phytoliths for wheat/barley type however show a slightly different picture, not only from the macrobotanical remains but also by site. At all sites and in all periods the data clustered in the triplots of leaf/stem, wheat/barley type husk and weedy type husk at the leaf/stem end, with occasional spread along the husk axis such as Dabli vas Chugta, Masudpur VII in the Late Harappan and Masudpur I (Fig. 5).

**Fig. 6:**
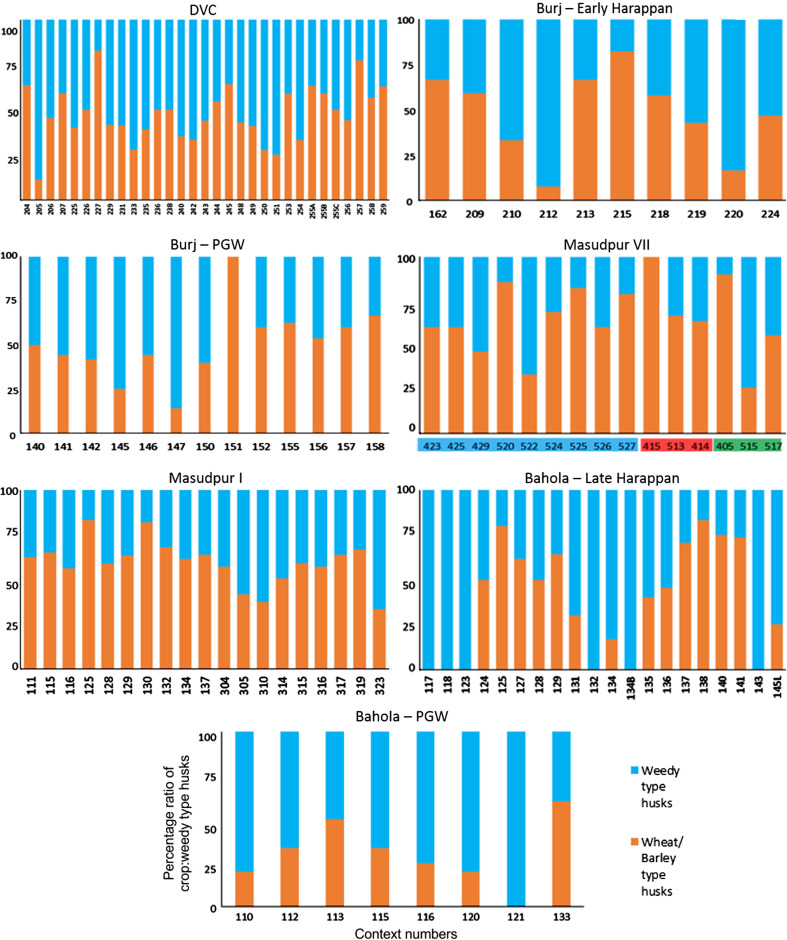
Ratio of wheat/barley type and weedy type husk phytoliths

The high proportion of leaf/stem could be a result of early stage processing like threshing at the settlement, but could equally represent the bringing of such crop processing by-products to the settlement for things such as bedding, roofing, and/or matting. It was thus decided that a focus on the relative proportion of inflorescence phytoliths would be more interesting, as it would help to explore which later processing stages were represented at each settlement. At all sites both crop and weedy type husks were present in the soil, suggesting that both chaff and weed seeds were still being removed from the cereal crop at the settlement. There were, however, some differences between sites. At Dabli vas Chugta and Burj in the Early Harappan, and to a degree Bahola in the Late Harappan period (although this site was unusual in that it had five contexts with only weedy types present) a mixture of weedy type dominant and crop type dominant samples was seen (Fig. 6).

At Burj in the PGW period, Masudpur VII in the Late Harappan and Bahola in the PGW period, the majority of samples had higher proportions of weedy type than crop type husk phytoliths, while at Masudpur VII in the Early Harappan, Mature Harappan and at Masudpur I the majority of samples had higher proportions of crop type husk phytoliths. These ‘higher proportions’ were, however, marginal, and only in a few cases were samples truly dominated by one of the other type, as with the samples with the weedy type only from Bahola Late Harappan. It is difficult to determine what the relationship between the proportions of weedy and crop type husks represent. For instance, was the increased input of these elements into the soil system due to the specific processing stage or could it reflect taphonomy and preservation issues, differential production or perhaps could it even be related more to actions such as fuel preference? Further studies on the impact of crop processing stages on phytolith input into soil are needed to further understand such interpretations. What these data do suggest is that there may have been earlier stages of processing such as the removal of wheat or barley husks at all sites that are not seen in the macrobotanical remains, as at all sites in all periods the phytoliths indicative of earlier stages of processing are present, and in some cases in greater proportions than weedy type phytoliths.

#### *Kharif* (summer) crops

Millet and rice have been dealt with separately as certain weeds could be assigned specifically to rice in the macrobotanical analysis (Moody 1989).

#### Millet macrobotanical and microbotanical remains

**Fig. 7:**
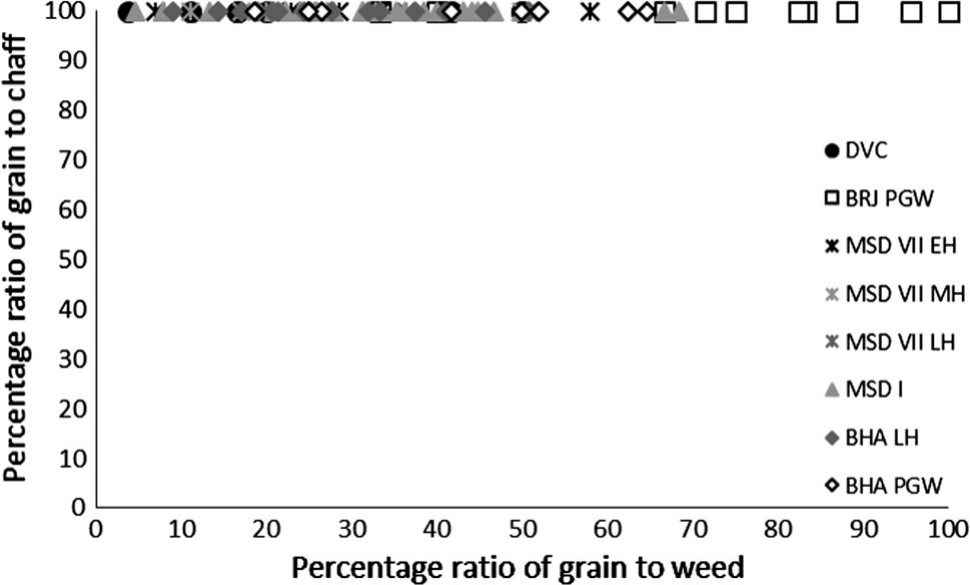
Ratio of millet grain–chaff, and millet–weed macro-remains

The macrobotanical remains of the summer crops of hulled millets and rice differ not only from the winter crops in their processing by-products, but also from one another. Millet provided no evidence for chaff, which is perhaps unsurprising as these small cereal grains produce extremely fragile rachillas that are easily destroyed in fire, and their absence from the macrobotanical remains is explained best through taphonomy (Reddy 1994, 1997, 2003). However, no chaff-fused millet grains were found, suggesting that parching was not the main stage of processing that resulted in the creation of these remains (Reddy 1994, 1997, 2003). The absence of chaff-fused millet grains could suggest that the parching stage was being carefully controlled to prevent loss of grain through burning. In general a range of weed to grain proportions was noted from grain rich contexts, including grain only samples, to weed rich contexts, with the former possibly indicating cooking accidents or taphonomically altered samples in which weeds had been destroyed by fire (Fig. 7).

**Fig. 8:**
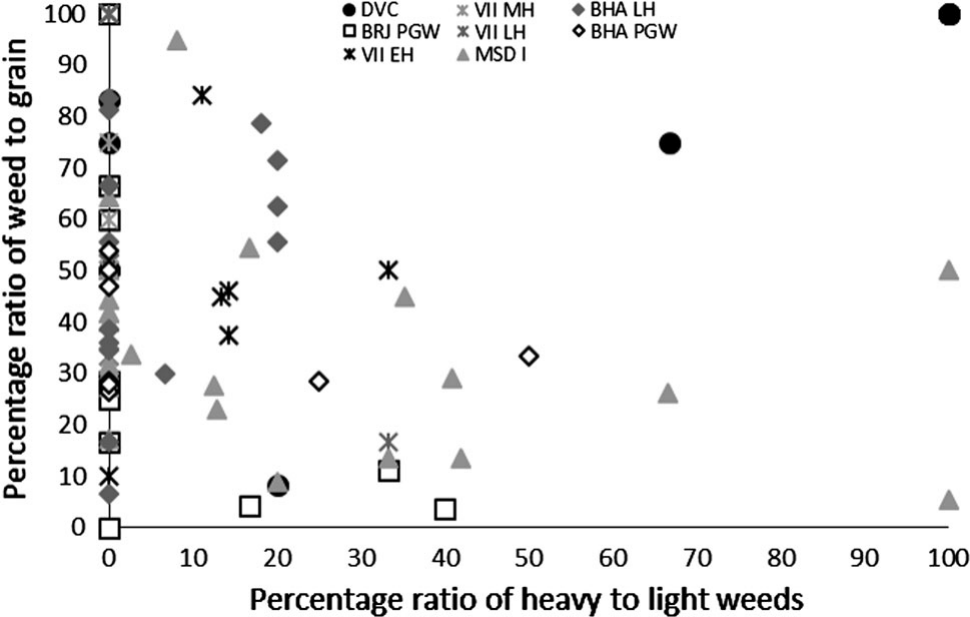
Ratio of weed seeds–millet grain, and heavy–light weed macro-remains

Again, no seed heads or seed headed weeds were noted, which suggests that that later stages of processing are indicated on the sites. As millets are winnowed to remove weed seeds rather than sieved, the weight of weeds was explored, and indeed the majority of weeds at all sites in all periods consisted of light weeds, which would have been removed by winnowing (Fig. 8).

Millet winnowing, unlike sieving, can be carried out in bulk and therefore is more likely to have taken place soon after harvest if labour is available (Reddy 1994, 1997, 2003). The fact that light weeds were still being removed, and in large proportion, at the settlements implies a lack of people available to carry out processing during harvest time. This could therefore imply a greater labour bottleneck after harvest during the *kharif* (summer) months than the *rabi* (winter) months, or that more effort was put into cleaning barley than millet before storage. Masudpur I was slightly different from the other sites in that it had a broader range of samples with differing weed weight proportions, including a couple of samples with heavy weed seeds dominating, which might suggest that slightly more bulk processing occurred at harvest time. However the majority of samples were still mainly light weed seeds, fitting with the general pattern that winnowing was still done on site as a regular part of daily life. Millet thus appears, according to the macrobotanical remains, to have required daily bulk processing before use.

**Fig. 9:**
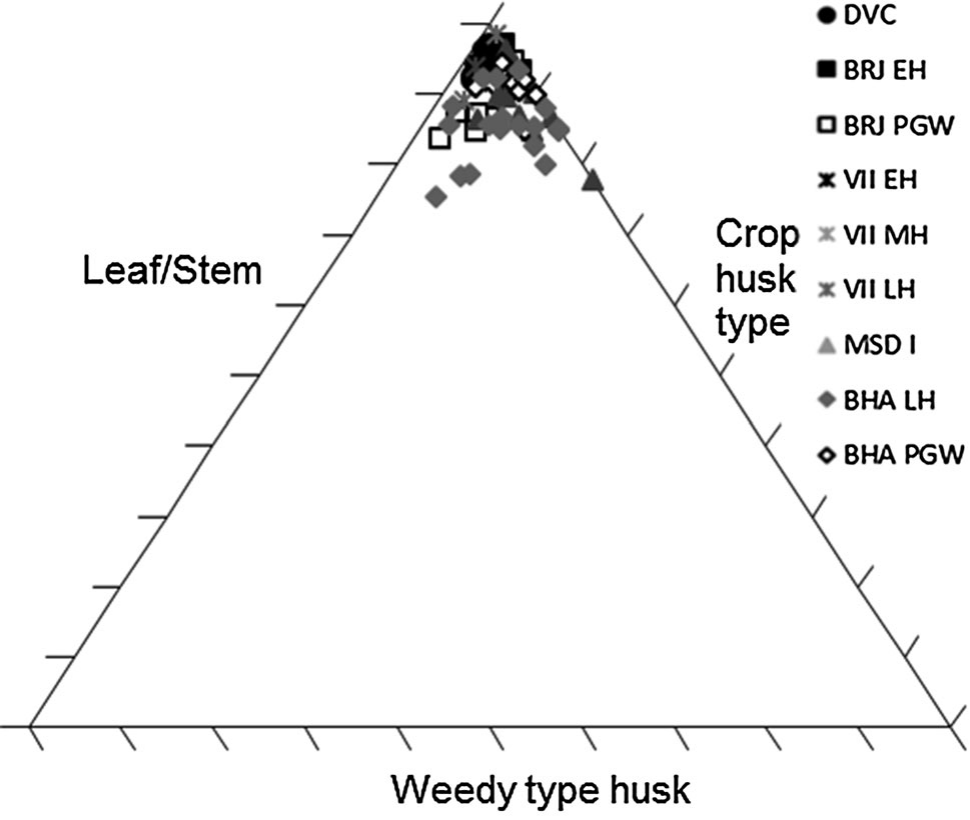
Triplot of leaf/stem, millet type husks and weedy type husk phytoliths

The millet type phytolith triplots of leaf/stem, weedy type husks and millet type husks showed similar patterns to one another across sites and periods. They were more clustered than the wheat/barley type patterns, suggesting less input of husk elements (Fig. 9).

**Fig. 10:**
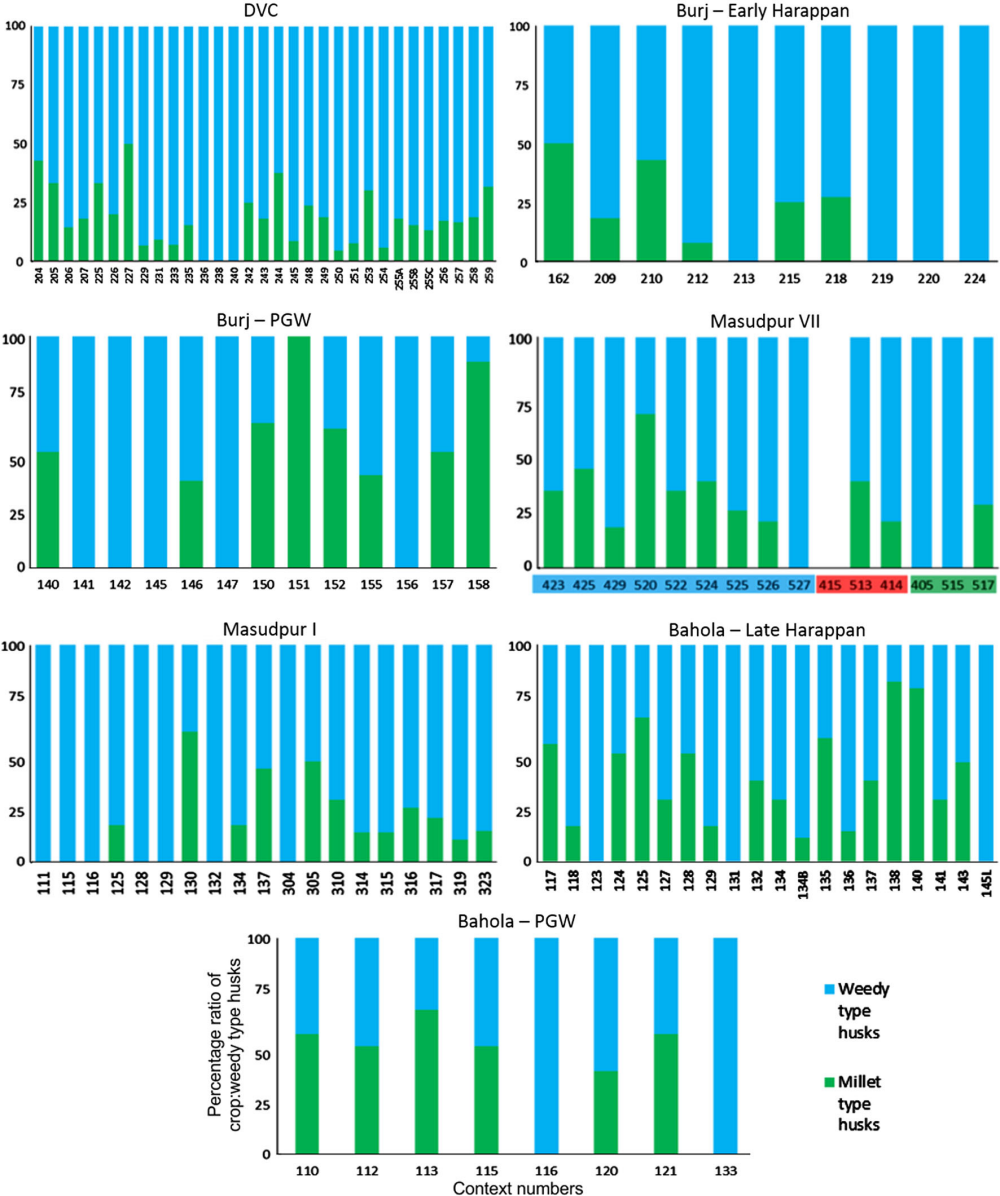
Ratio of millet type and weedy type husk phytoliths

To explore the relative proportion of the husk elements, the weedy types and millet types were compared and in general showed that millet husks were not commonly found at the sites, supporting to a degree the macrobotanical remains (Fig. 10).

These were not wholly absent, however, suggesting that some differential loss had occurred in macrobotanical preservation. The assemblages were dominated by weedy types at Dabli vas Chugta, Burj in the Early Harappan period, at Masudpur VII in the Early Harappan period, Mature Harappan period (with the exception of one sample with no remains at all of weedy or millet type chaff—unsurprising given their lack in the macrobotanical remains), Late Harappan period and Masudpur I (with the exception of one crop dominated sample). Masudpur I also presents some interesting patterns as the millet chaff presence is almost entirely confined to contexts originating in trench XM2. This could imply a spatial distribution of processing stages at Masudpur I, but more samples would be needed to explore this further. At Bahola in the Late Harappan period and at Burj and Bahola in the PGW periods there was a more even mix of crop and weed dominated samples, although as in all cases the notion of ‘high proportions’ is slight. It might be suggested that there was a change then in the later periods towards the inclusion of more millet chaff in the assemblages, which might suggest more late stage processing remains were being included into the soil alongside the weeds of the earlier stages, although as with the wheat and barley types, relating proportions to stages of processing would require further ethnographic work. The presence of millet type husk phytoliths does however suggest that their absence from the macrobotanical samples may be due to taphonomic processes, particularly the destruction by charring of the fragile rachilla or lemma/palea, and this shows the importance of a multi-proxy approach to millet crop processing analysis.

#### Rice macrobotanical and microbotanical remains

**Fig. 11:**
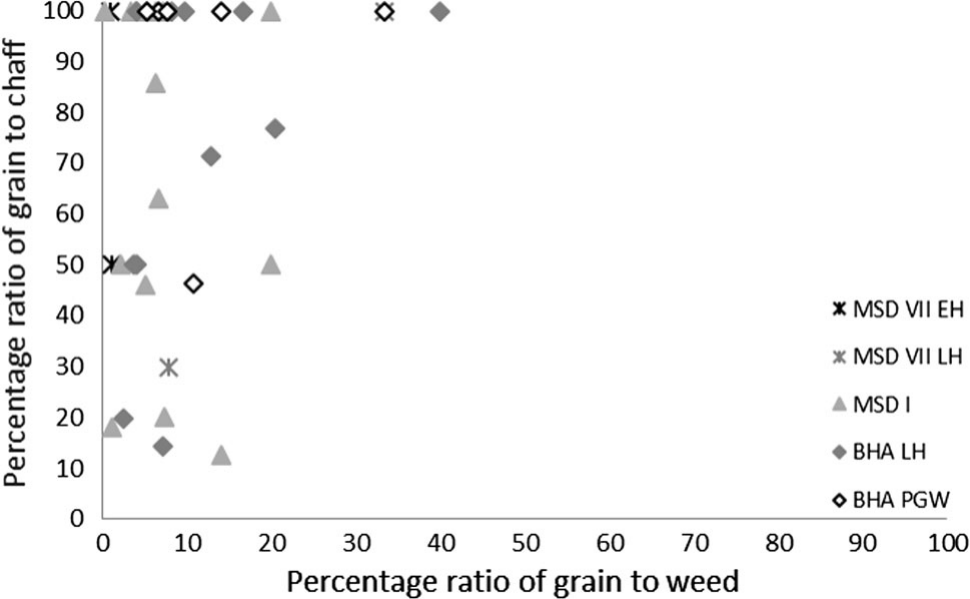
Ratio of rice grain–chaff, and rice grain–weeds macro-remains

**Fig. 12:**
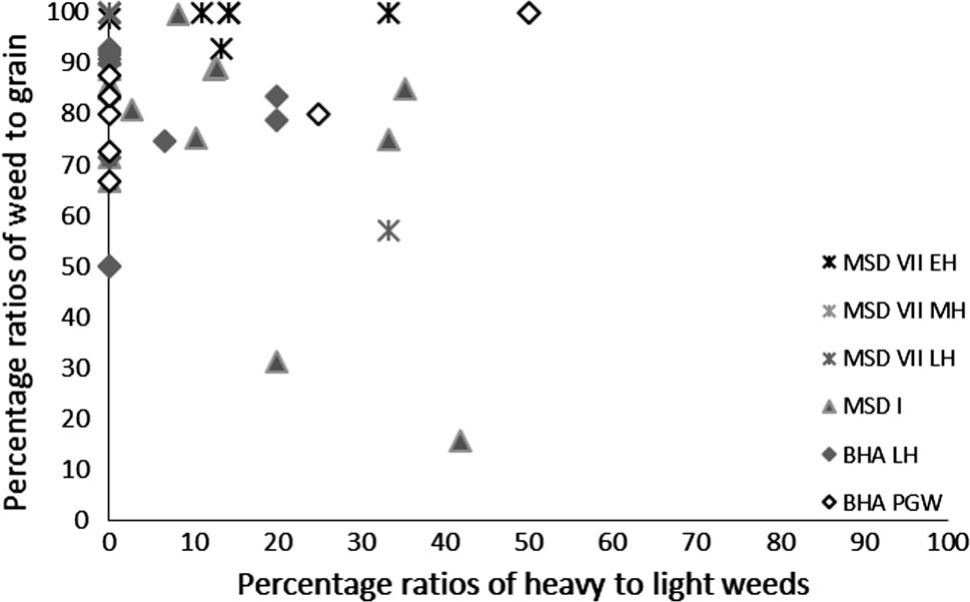
Ratio of weed seeds–rice grain, and heavy–light weed macro-remains

Rice macrobotanical remains were present only at Masudpur VII, Masudpur I and Bahola (Figs. 11, 12).

At Masudpur VII in the Early Harappan period the number of contexts containing rice was small (n = 2), so interpretations are cautious. Chaff, in the form of spikelet bases, was found in one context, and these elements are removed in second winnowing (ESM 2). The two samples with rice remains also contained high proportions of summer weed seeds, and in one sample (428) these were light weed seeds only, which suggests that winnowing was carried out, while in the other (429), which contained the spikelet bases, the light weed seeds were accompanied by some heavy weed seeds. Whether these were specifically rice weeds or those from the millet processing is difficult if not impossible to ascertain, but it could suggest both that rice was stored early like millet, after threshing, but before the first winnowing. In the Mature Harappan period at Masudpur VII the picture for rice becomes more complex as although no rice grains were found; there were rice spikelet bases present in a single context (514). This context was not abutting any earlier or later period contexts, and did not show any signs of disturbance that might lead to contamination. The chaff could also have been accidentally brought from another area of the settlement for a specific purpose like pottery temper, although the pottery does not show high proportions of chaff tempering (Parikh and Petrie in press). The evidence from this one context does, however, suggest that rice may have been processed and consumed in the Mature Harappan period at Masudpur VII and that some processing of the grain from the second winnowing stage onwards may have been taking place on site. The small number of samples from the Late Harappan period at Masudpur VII (n = 3) results again in a cautious interpretation, but rice was present in two of these contexts, and one of these also contained spikelet bases (515). Context 515 also contained weed seeds. These were mainly light weed seeds, which suggests that they represent a crop that required both first winnowing, indicated by light weed seeds, and second winnowing, indicated by spikelet bases (ESM 2). The presence of heavy weed seeds also suggests that hand sorting contributed to the formation of this assemblage. The other context (517) had grain only, which might indicate a cooking accident, taphonomic processes or differential preservation. At Masudpur I in the Mature Harappan and Bahola in the Late Harappan and PGW periods the picture was similar. Rice chaff was present in most samples, but the samples were mainly grain rich, and compared with chaff, the weed seeds were mainly light, suggesting storage before first winnowing. Evidence for hand sorting was seen in the small numbers of heavy weed seeds found. As such, based on the macrobotanical remains alone, it would appear that rice at all three sites was being stored before first winnowing, and that this stage may have been carried out on site, or that the waste was brought to the settlements and reached fire perhaps as fuel, a component of fuel or through other processes of waste disposal.

However, when looking at the phytoliths, a different picture emerges. The phytoliths for rice are extremely difficult to discuss as only one double-peak husk phytolith associated with rice chaff was found, at Burj in the PGW period, which is a site with no evidence for rice exploitation in the macrobotanical remains. Some evidence for Ehrhartoideae, of which the rice family is part, was seen in the form of scalloped fan-shaped bulliforms, but these can come from a range of plants within the Ehrhartoideae, and are found in a range of tissues within the plant, not just husks. As such they cannot be used to inform on crop processing stages. The lack of rice double-peak husks in the phytoliths therefore creates an interesting conundrum in terms of establishing which dataset represents on site processing. The only rice chaff found at the sites with rice macro-remains were spikelets, which are removed at the same stage as the lemma and palea in which the distinctive double-peaked husk phytoliths of rice are found. Different processing stages and taphonomic issues such as sweeping or even the use of chaff for fuel therefore cannot explain the lack of phytoliths, but the presence of spikelet bases. One possibility is the use of spikelet bases as temper in pottery, but there is a lack of chaff tempered pottery at the sites (Parikh and Petrie in press). Rice actively takes up silicon (Ma et al. 2006) and up to 10 % of the dry weight of the plant can be silicon. However, low silicon rice mutation has also been discovered in wild rice, a situation which makes the rice less resistant to disease and pests (Ma et al. 2006). Indeed Scott–Cummings (personal communication) has suggested that although rice husks are often highly silicified, this is not always the case and she has observed poorly silicified stands of rice plants. However, as Ma et al. (2006) note, low silicon rice mutations are found in less than one tenth of the wild populations that they studied. At the same time it can also be noted that both wild-type and domesticated-type rice were present at these sites, which suggests that the potential for high silicon uptake rice is more likely to have been the norm than the rarer low silicon uptake form, and as such poorly silicified rice does not provide a good argument to explain the lack of phytoliths but presence of spikelet bases. Differential preservation of silicon phytoliths compared with charred spikelet bases could explain the differences, but further work is required to explore this. Given that spikelets are present in the macrobotanical remains but not phytoliths, the macrobotanical interpretations are preferred for evidence of rice processing analysis, until further work on the phytolith preservation is undertaken.

## Discussion and Conclusions

Some initial interpretations can be made regarding the processing stages carried out for the various cereals at the sites being discussed (Table 4).

**Table 4:** Summary of main findings

	Main conclusions
Macrobotanical	– Wheat/barley showed fewer processing stages, with the main focus on mid-processing stage of fine sieving – Millet and rice showed all processing stages were present on site with the main focus on early processing of winnowing
Phytoliths	– Wheat/barley showed all stages including early processing were present – Millet processing showed more late stage than early stage – Little rice chaff: taphonomy? But weeds showed similar pattern to millet—late stage processing was present
Combined datasets	– Wheat/barley showed less final stage cleaning on site, but both early and mid-stage processing were present – Millet and rice showed all stages present and carried out regularly on sites – Macrobotanical grain analysis (Bates 2016; Bates et al. in press; Petrie et al. in press c) suggested that millets were more regularly used and in greater proportions on these sites than wheat/barley and to a lesser extent rice

The macrobotanical analysis suggests that the *rabi* (winter) cereals of wheat and barley consistently had fewer processing stages than *kharif* (summer) crops of rice and millet. The wheat and barley data suggest that fine sieving was the main stage carried out on all sites, with some hand sorting. Rare evidence for chaff in the form of rachis internodes potentially suggests that some earlier stages of processing were also being carried out. In contrast, the presence of light weed seeds in the *kharif* (summer) assemblage provided evidence for winnowing of summer cereals, which is a stage that can usually be carried out in bulk close to harvest. As such, it could be argued that the macrobotanical remains indicate that the *kharif* crops were stored at all sites closer to the harvest than the *rabi* (winter) crops, and that this might imply differences in labour organisation at harvest dependant on the season of cropping (Stevens 1996, 2003; Fuller et al. 2014). The different processing approaches used for the various cereals indicated by the macrobotanical remains suggest that the seasonality of cropping drove the labour division and decisions relating to labour organisation at these settlements, rather than the time period or the geographical location of the settlements. It is notable that the macrobotanical remains imply that *rabi* (winter) cereal processing was more centralised than *kharif* (summer) processing. Alternative explanations are also possible, and processing waste from these bulk stages may have been incorporated in fuel differing either in what parts of the plant were being fed to animals and thus incorporated into dung fuel at differing times of the year, perhaps through differing grazing and foddering practices dependant on season, or through what was being incorporated into dung or used as additional fuel to increase fuel potential at different seasons.

The phytolith data alters this picture somewhat as at all sites, wheat and barley type and millet type inflorescence phytoliths were noted, filling in a possible ‘taphonomic gap’ in the macrobotanical evidence. The data for the *rabi* (winter) cereals of wheat and barley suggests that waste from an earlier stage of processing, from winnowing and/or coarse sieving, which was not evident from the macrobotanical remains, was present on-site. This was either as the by-products of these activities being carried out on site or of the material being brought to site through other routes, such as inclusion in fuel. The millet remains, on the other hand, suggested that although some chaff removal was being done at or around the settlements, weed seed removal produced the bulk of the waste until the Late Harappan periods, which is a pattern not seen in the macrobotanical remains. The phytoliths thus suggest that storage was similar for summer and winter cereals, implying evidence for more early stage rather than late stage processing at the settlements, and therefore a lack of bulk processing before storage. A slight shift in millet processing in the later periods was noted, perhaps implying some increasing centralisation in millet processing. Based on the models outlined by Stevens (1996, 2003) and Fuller et al. (2014), it could be argued therefore that these patterns imply that household processing was the norm for wheat, barley and millet throughout the Early, Mature and Late Harappan periods, at least at the sites being investigated, but also perhaps more broadly.

When combined however, the macrobotanical and phytolith data highlights the importance of the dual-proxy approach to crop processing analysis despite the difficulties regarding the rice remains. Barley and wheat showed fewer processing stages, less late-stage processing and, although often found in larger proportions, were generally less frequently used at the settlements than millets (Bates 2016). The low ubiquity and less processing implies that *rabi* (winter) cereals of wheat and barley were less commonly used and less regularly processed all the way through to cleaned grain than small-hulled millets, indicating that millets may have played a more important role in the daily lives and diets of people living at these settlements.

The possibility that seasonality may have affected labour organisation is contrary to many of the models of crop processing that have been developed for the Indus Civilisation and also to models that have explored the impact of urbanisation on village labour organisation. Many of these models have suggested that crop processing was centralised in the Mature Harappan period and devolved to households in the Late Harappan period, either as a socio-economic response to cope with climate change (Madella and Fuller 2006) or as a result of social change (Kenoyer 2000). These results presented here suggest that more complex models need to be considered for settlements where a complex mix of cropping seasons and cereal taxa were exploited.

The role of millets and also rice, as well as wheat and barley as regular parts of both the annual cropping cycle and the daily lives of people at these settlements implies that the inhabitants were well adapted to a variable environment (Petrie et al. in press a, b, c). Evidence for pre-urban adaptation to local climatic and environmental variability also suggests that a hypothetical switch towards millets as a possible precursor to urban decline (Madella and Fuller 2006) is not applicable in this region. The stability in the processing strategies for all cereals at these settlements is indicated by the fact that there was little variation over time, and implies that as cities rose and fell around these villages, the rural agricultural strategies continued as before, with household-scale processing being the norm.

## Electronic supplementary material

Below is the link to the electronic supplementary material.

Supplementary material 1 (DOC 8029 kb)

Supplementary material 2 (DOCX 1138 kb)

Supplementary material 3 (DOCX 9311 kb)
