# Supplementary material for: Exploring Indus crop processing: combining phytolith and macrobotanical analyses to consider the organisation of agriculture in northwest India c. 3200–1500 bc
Source: Veg Hist Archaeobot. 2016 May 21;26(1):25–41. doi: 10.1007/s00334-016-0576-9 (PMC7346983; doi:10.1007/s00334-016-0576-9)
Supplement: Supplementary file 1 — Supplementary material 1 (DOC 8029 kb) [file 334_2016_576_MOESM1_ESM.doc]

**Exploring Indus crop processing: combining phytoliths and macrobotanical analysis to consider the organisation of agriculture in northwest India c. 3200-1500 BC**

Jennifer Bates^1^, Ravindra Nath Singh^2^, Cameron A. Petrie^1^

*^1^ Division of Archaeology, University of Cambridge, Downing Street, Cambridge, CB2 3DZ, UK, e-mail: jb599@cam.ac.uk*

*^2^ Department of AIHC and Archaeology, Banaras Hindu University, Varanasi – 221005, India*

Supplementary Information 1: Materials and methods


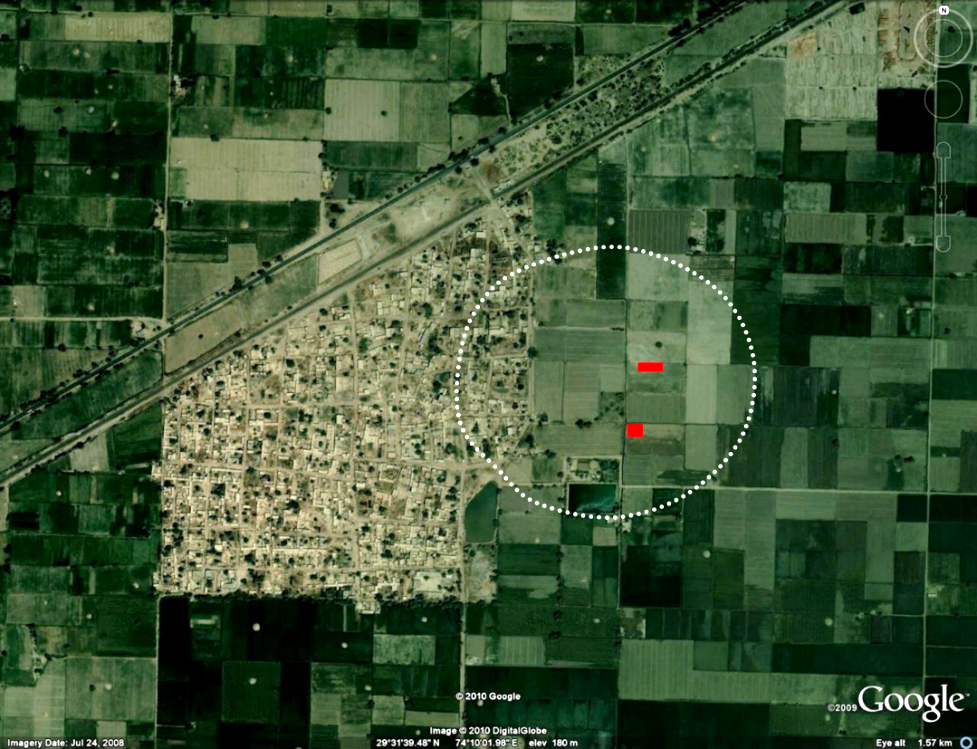


**Fig. S1** Location of trenches ZA6 and ZI7 at Dabli vas Chugta (adapted from Singh et al. 2012: Fig.3, used with permission)


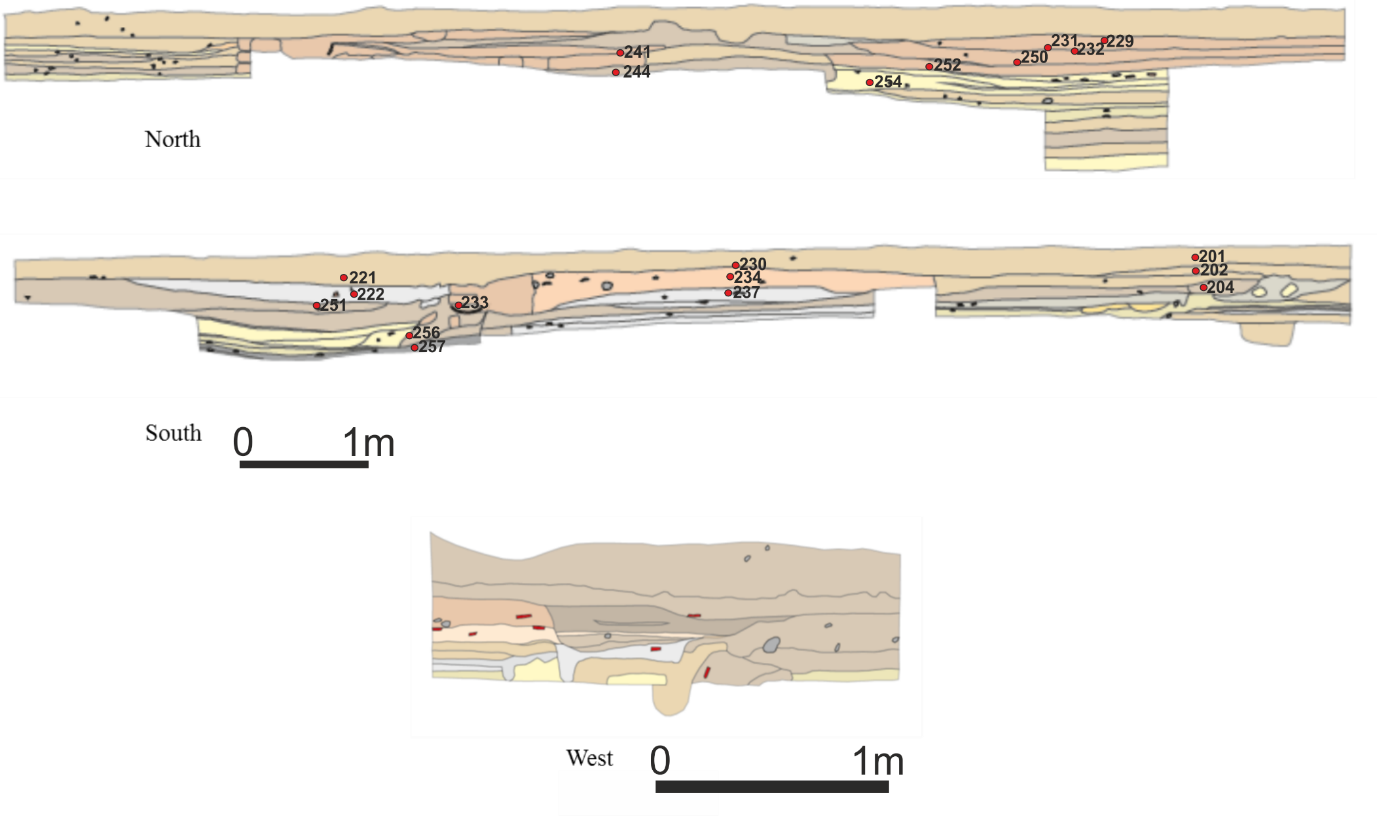


**Fig. S2** Stratigraphy of trench ZI7 at Dabli vas Chugta including relative location of some of the samples analysed (Bates 2016, used with permission)

**Table S1** Samples analysed at Dabli vas Chugta (Bates 2016)

| **Trench** | **Context** | **Context Type** | **Period** | **Macro** | **Phytolith** |
| --- | --- | --- | --- | --- | --- |
| ZI7 | 204 | Fill | Early Harappan | X | X |
|  | 205 | Hearth |  | X | X |
|  | 206 | Pit |  | X | X |
|  | 207 | Fill |  | X | X |
|  | 223 | Structural |  | X |  |
|  | 224 | Collapse |  | X | X |
|  | 225 | Hearth |  | X | X |
|  | 226 | Pit |  | X | X |
|  | 227 | Pit |  | X | X |
|  | 229 | Pit |  | X | X |
|  | 230 | Collapse |  | X | X |
|  | 231 | Fill |  | X | X |
|  | 233 | Pit |  | X | X |
|  | 234 | Collapse |  | X | X |
|  | 235 | Fill |  | X | X |
|  | 236 | Fill |  | X | X |
|  | 238 | Fill |  | X | X |
|  | 239 | Collapse |  | X | X |
|  | 240 | Fill |  | X | X |
|  | 241 | Surface |  | X |  |
|  | 242 | Fill |  | X | X |
|  | 243 | Surface |  | X | X |
|  | 244 | Fill |  | X | X |
|  | 245 | Surface |  | X | X |
|  | 248 | Hearth |  | X | X |
|  | 249 | Ashy fill |  | X | X |
|  | 250 | Surface |  | X | X |
|  | 251 | Fill |  | X | X |
|  | 252 | Collapse |  | X | X |
|  | 253 | Pit |  | X | X |
|  | 254 | Fill |  | X | X |
|  | 255 A | Hearth |  | X | X |
|  | 255 B | Hearth |  | X | X |
|  | 255 C | Hearth |  | X | X |
|  | 256 | Surface |  | X | X |
|  | 257 | Surface |  | X | X |
|  | 258 | Ashy fill |  | X | X |
|  | 259 | Surface |  | X | X |


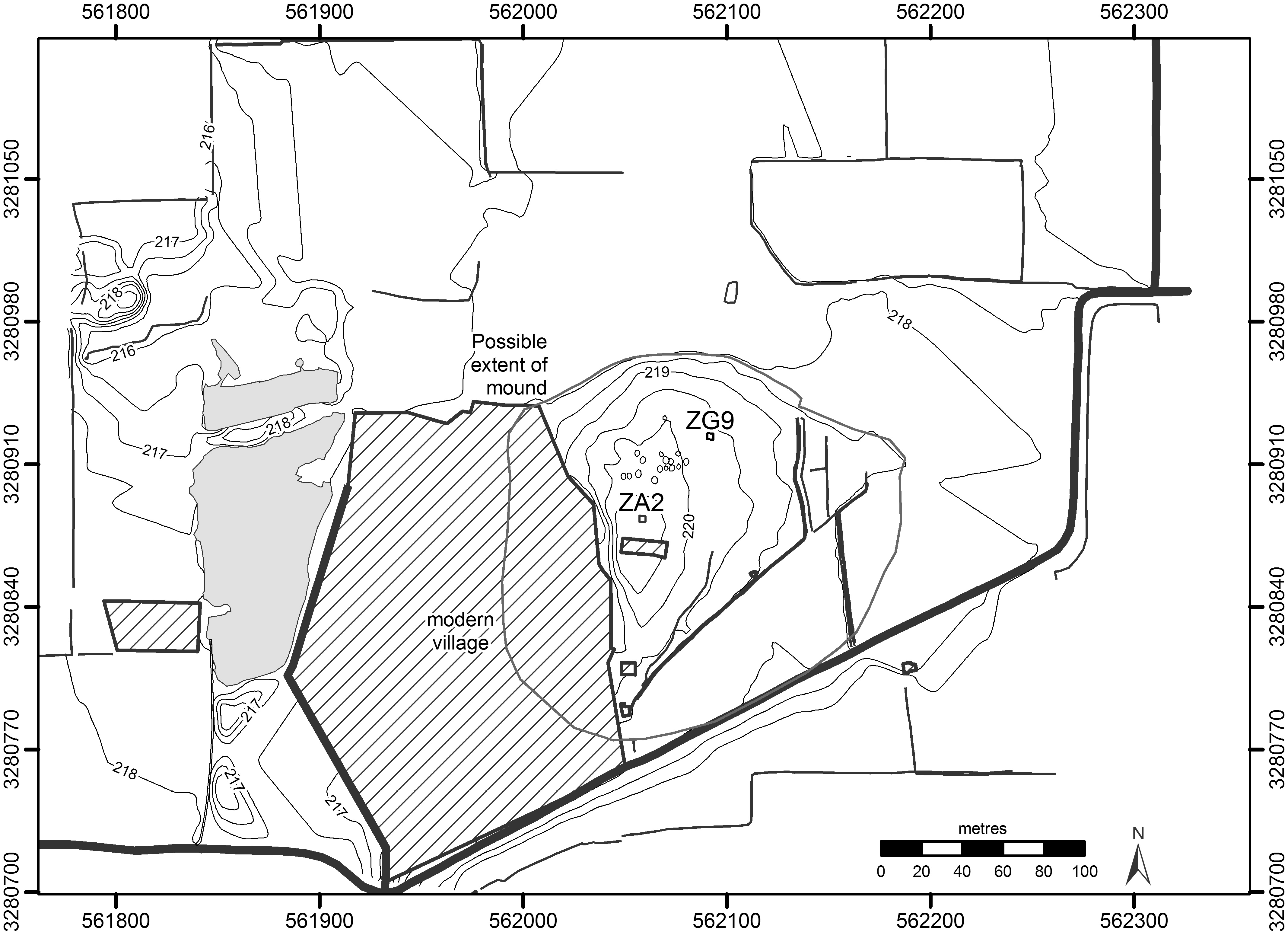


**Fig. S3** Location of trenches ZA2 and ZG9 at Burj (Singh et al. 2010a: Fig. 2, used with permission)


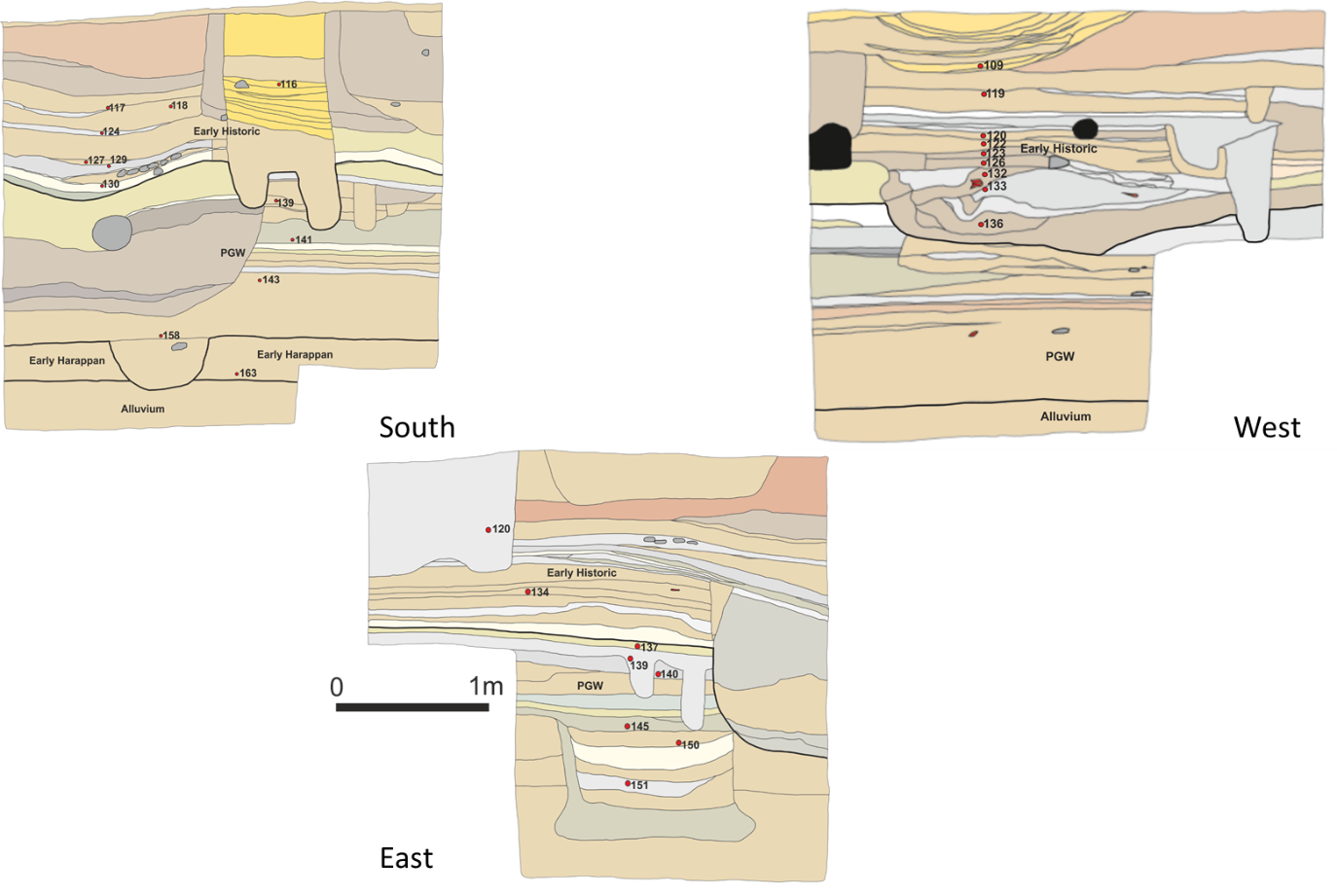


**Fig. S4** Stratigraphy of trench ZA2 at Burj including relative location of some of the samples analysed and phasing (Bates 2016, used with permission)


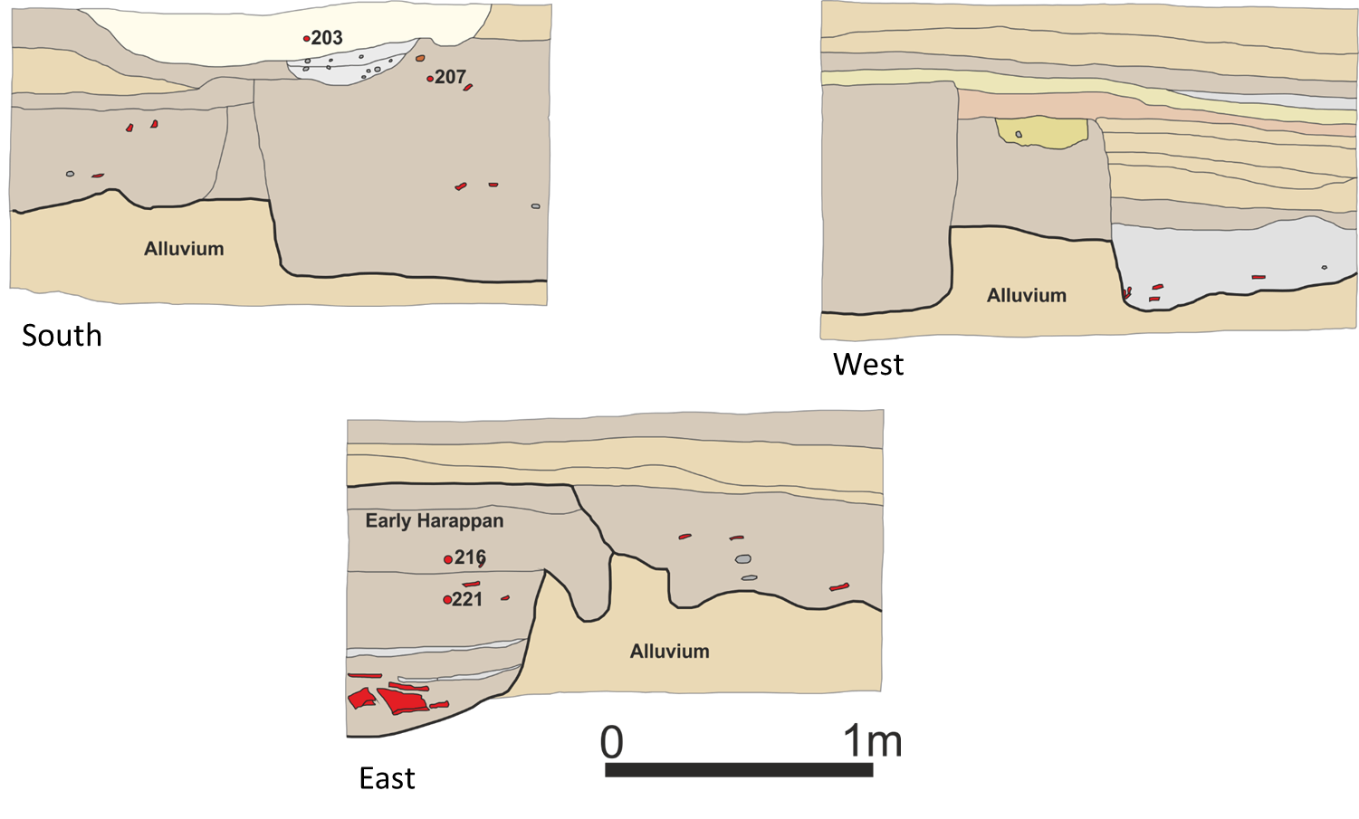


**Fig. S5** Stratigraphy of trench ZG9 at Burj including relative location of some of the samples analysed and phasing (Bates 2016, used with permission)

**Table S2** Samples analysed at Burj (Bates 2016)

| **Trench** | **Context** | **Context Type** | **Period** | **Macro** | **Phytolith** |
| --- | --- | --- | --- | --- | --- |
| ZA2 | 140 | Surface | PGW | X | X |
|  | 141 | Surface |  | X | X |
|  | 142 | Surface |  | X | X |
|  | 145 | Pit |  | X | X |
|  | 146 | Pit/Posthole |  | X |  |
|  | 147 | Pit/Posthole |  | X |  |
|  | 148 | Pit/Posthole |  | X |  |
|  | 150 | Pit |  | X | X |
|  | 151 | Pit |  | X | X |
|  | 152 | Pit lining |  | X | X |
|  | 156 | Fill |  | X |  |
|  | 157 | Pit |  | X | X |
|  | 158 | Pit |  | X | X |
|  | 162 | Pit |  | X | X |
| ZG9 | 209 | Fill | Early Harappan | X | X |
|  | 210 | Pit |  | X | X |
|  | 212 | Fill |  |  | X |
|  | 213 | Pit |  | X | X |
|  | 215 | fill |  |  | X |
|  | 216 | Fill |  | X |  |
|  | 218 | Fill |  |  | X |
|  | 219 | Surface |  | X | X |
|  | 220 | Fill |  | X | X |
|  | 221 | Fill |  | X |  |
|  | 224 | Fill |  |  | X |


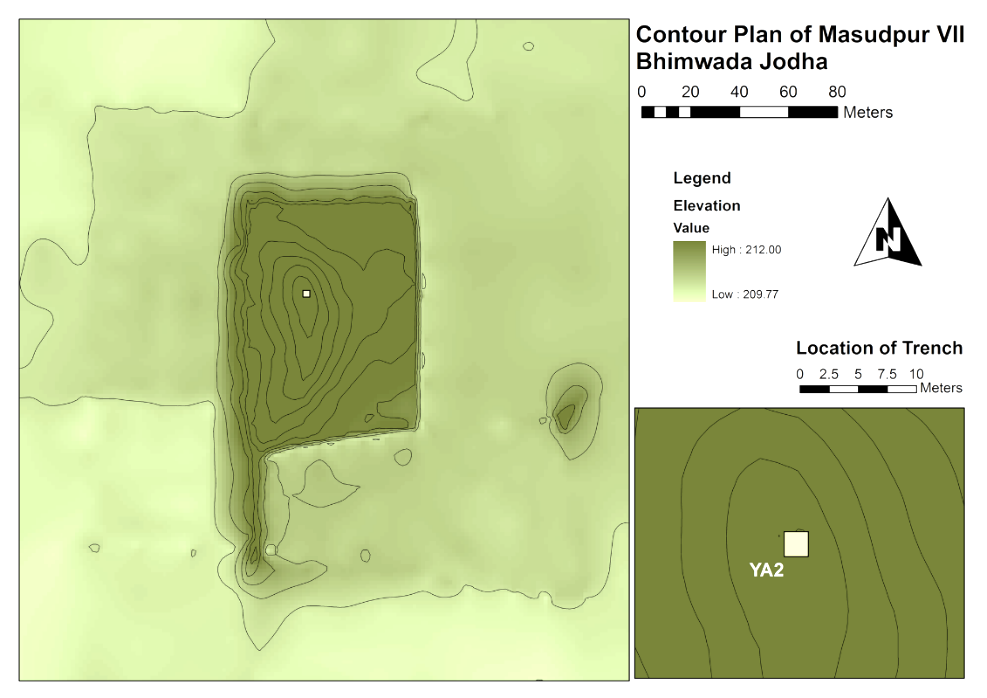


**Fig. S6** Location of trenches YA2 and YB1 at Masudpur VII (adapted from Petrie et al.2009: Plate 2, used with permission)


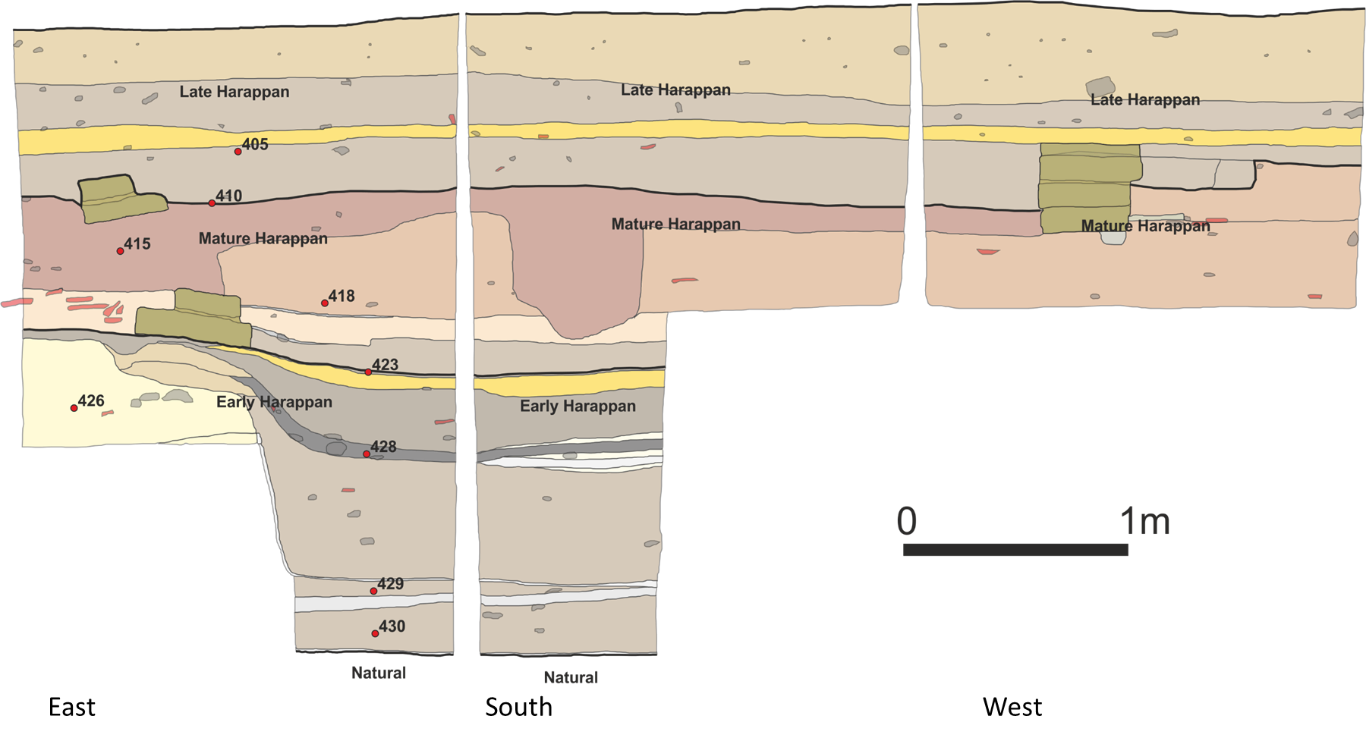


**Fig. S7** Stratigraphy of trench YZ2 at Masudpur VII including relative location of some of samples analysed and phasing (Bates 2016, used with permission)


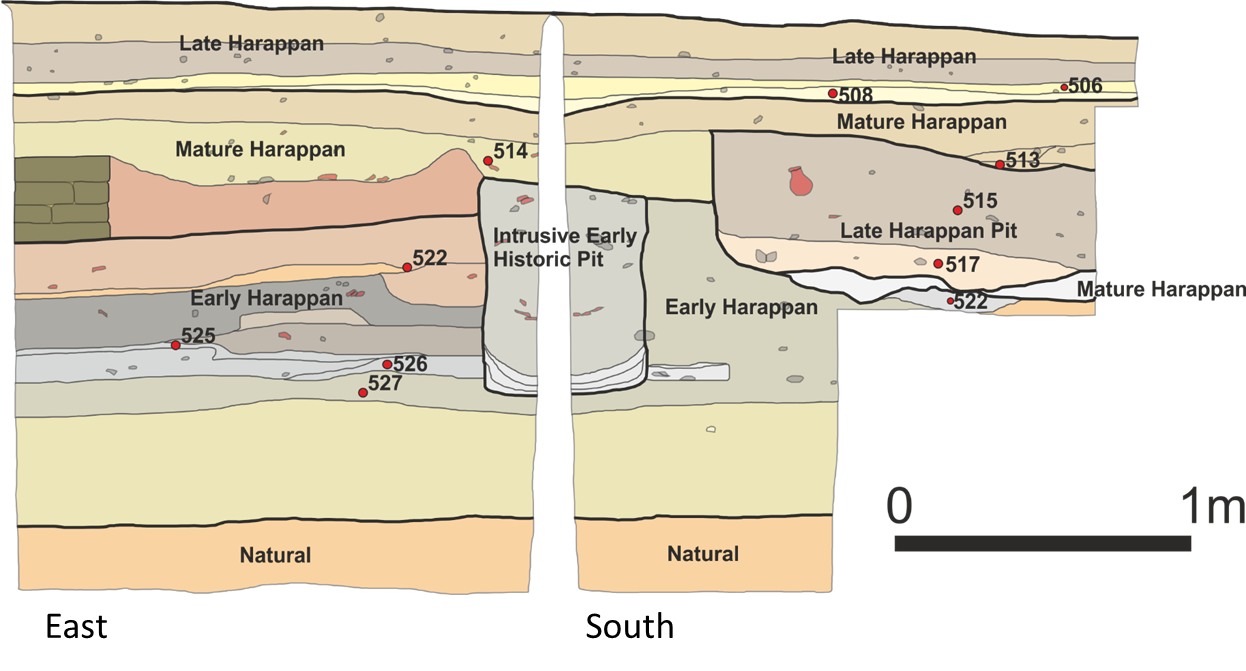


**Fig. S8** Stratigraphy of trench YB1 at Masudpur VII including relative location of some of samples analysed and phasing (Bates 2016, used with permission)

**Table S3** Samples analysed at Masudpur VII (Bates 2016)

| **Trench** | **Context** | **Context Type** | **Period** | **Macro** | **Phytolith** |
| --- | --- | --- | --- | --- | --- |
| YA2 | 405 | Surface | Late Harappan |  | X |
|  | 406 | Fill | Mature Harappan | X |  |
|  | 407 | Fill |  | X |  |
|  | 410 | Fill |  | X |  |
|  | 409 | Collapse |  | X | X |
|  | 414 | Pit |  | X | X |
|  | 415 | Ashy fill |  | X | X |
|  | 418 | Collapse |  | X | X |
|  | 419 | Fill |  | X |  |
|  | 422 | Fill |  | X |  |
|  | 422 burning | Ashy fill |  | X |  |
|  | 423 | Pit | Early Harappan | X | X |
|  | 425 | Fill |  | X | X |
|  | 428 | Pit |  | X |  |
|  | 429 | Pit |  | X | X |
|  | 426 | Fill |  | X |  |
|  | 430 | Fill |  | X |  |
| YB1 | 508 | Collapse | Late Harappan | X | X |
|  | 515 | Fill |  | X | X |
|  | 517 | Fill |  | X | X |
|  | 513 | Fill | Mature Harappan | X | X |
|  | 514 | Collapse |  | X | X |
|  | 520 | Pit | Early Harappan |  | X |
|  | 522 | Ashy fill |  | X | X |
|  | 524 | Surface |  |  | X |
|  | 525 | Fill |  | X | X |
|  | 526 | Fill |  | X | X |
|  | 527 | Fill |  | X | X |


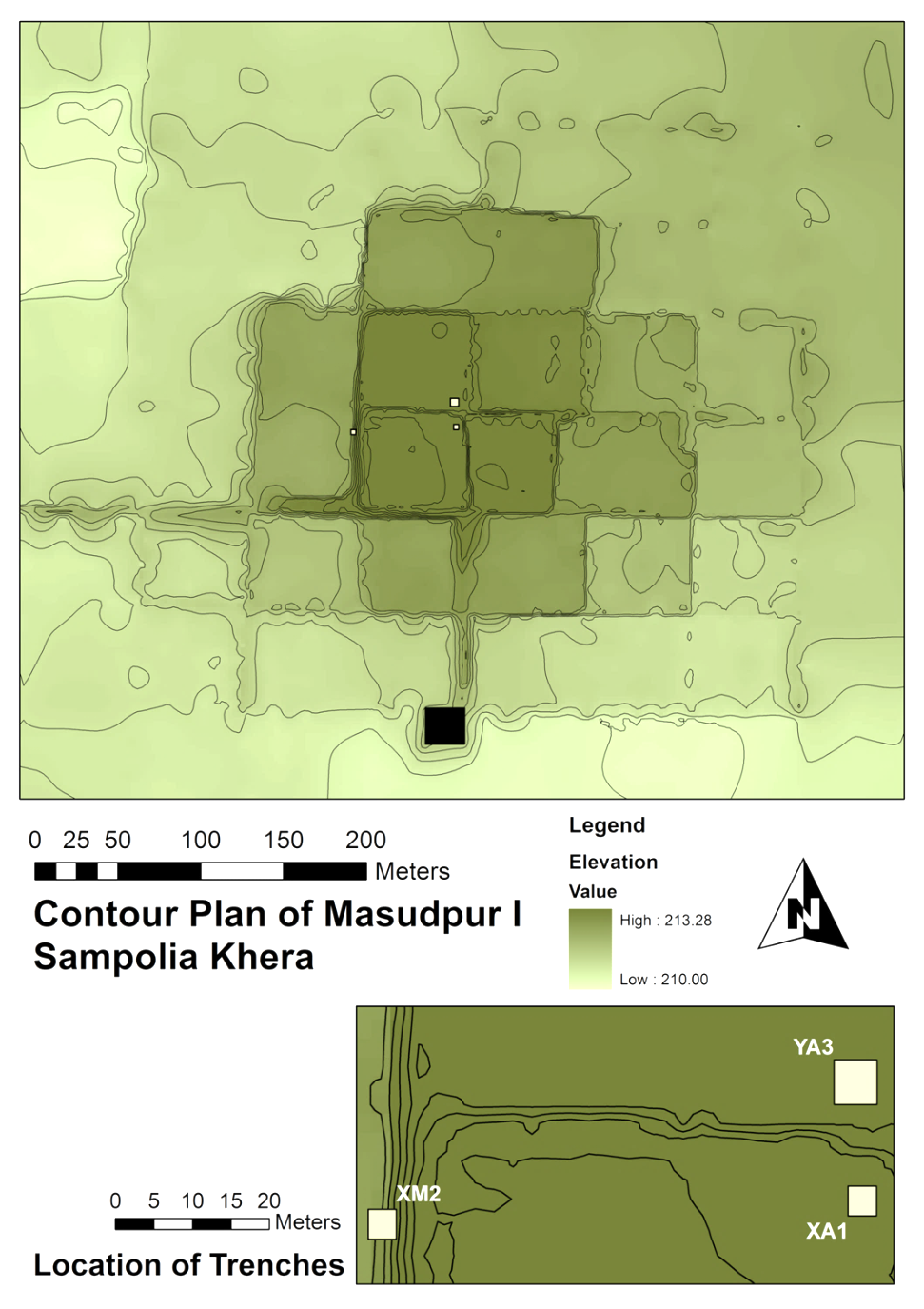


**Fig. S9** Location of trenches XM2, YA3 and XA1 at Masudpur I (adapted from Petrie et al. 2009: Plate 1, used with permission)


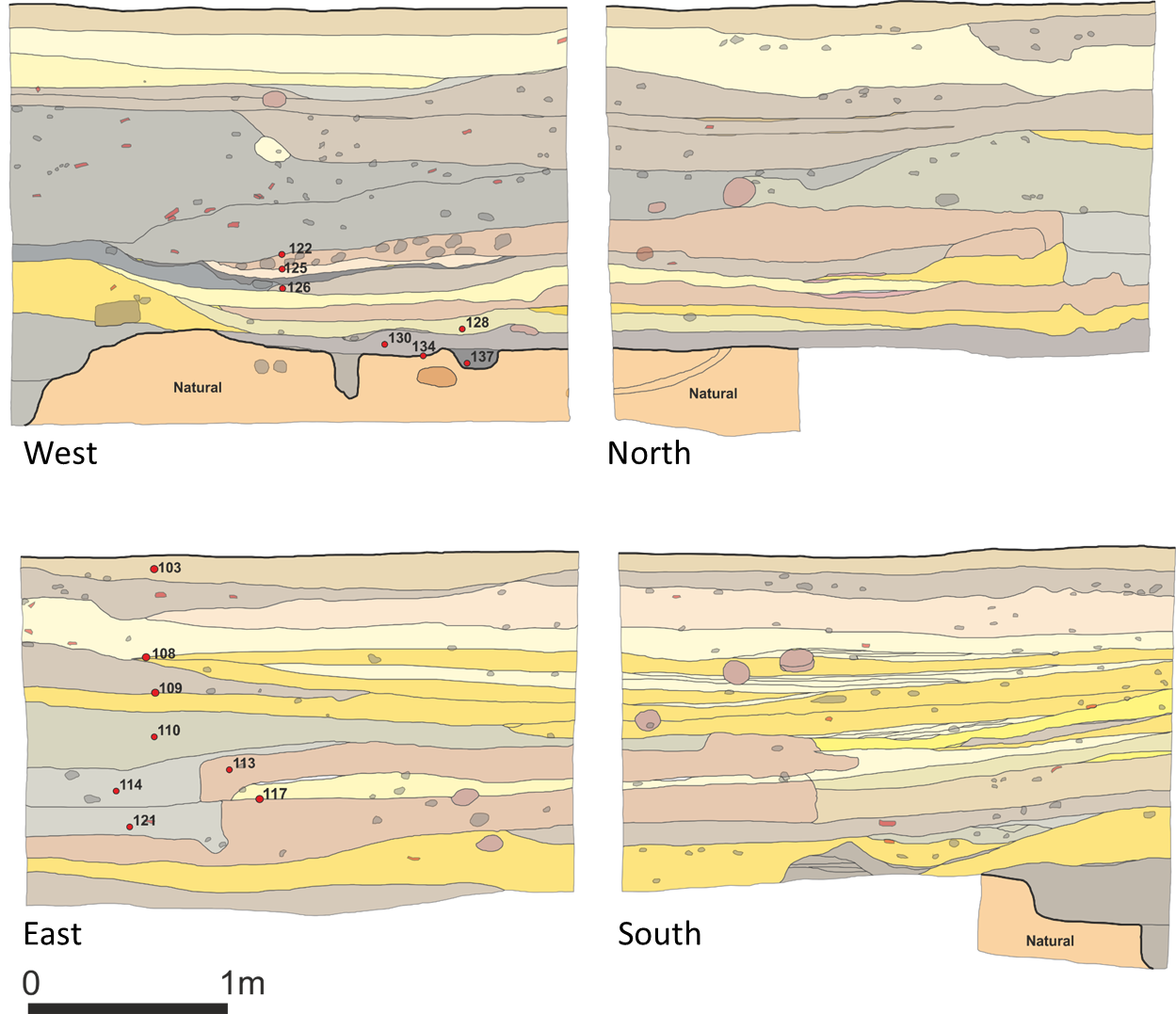


**Fig. S10** Stratigraphy of trench XA1 at Masupdur I showing relative location of some of the samples analysed (Bates 2016, used with permission)


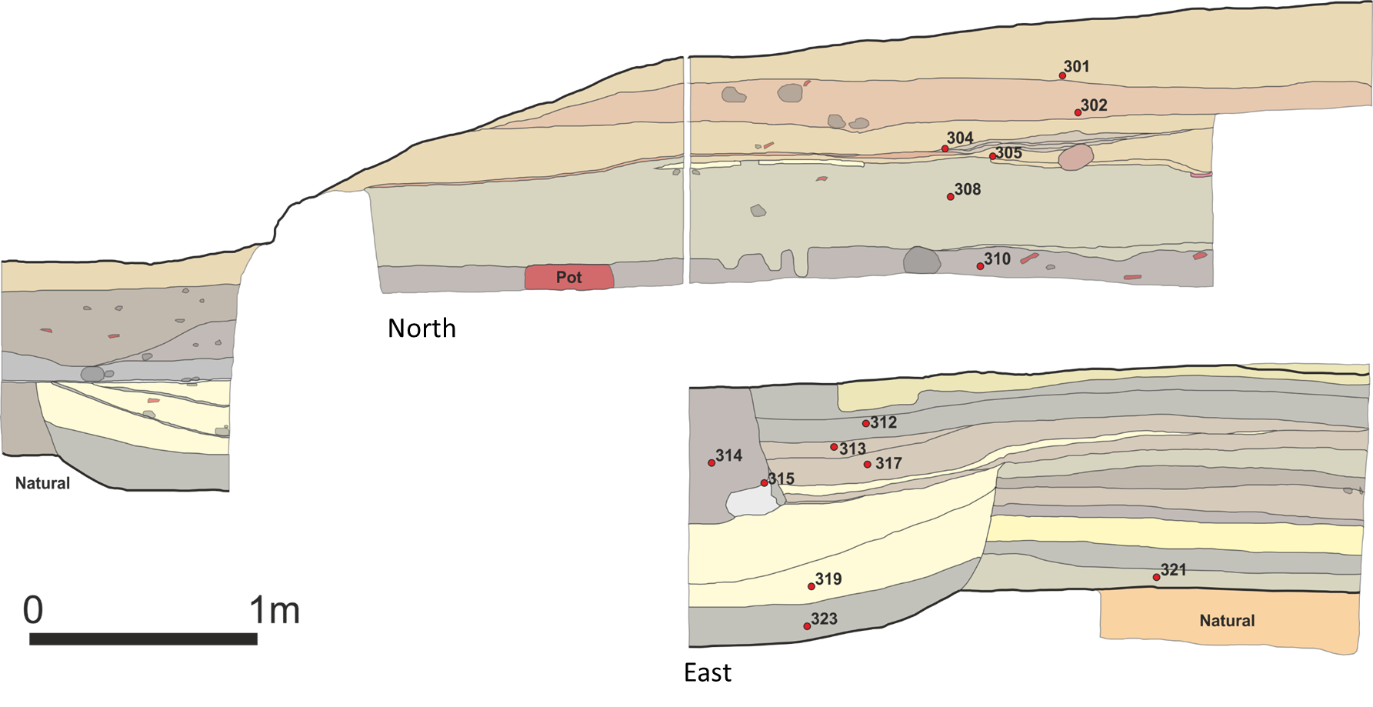


**Fig. S11** Stratigraphy of trench XM2 at Masupdur I showing relative location of some of the samples analysed (Bates 2016, used with permission)

**Table S4** Samples analysed at Masudpur I (Bates 2016)

| **Trench** | **Context** | **Context Type** | **Period** | **Macro** | **Phytolith** |
| --- | --- | --- | --- | --- | --- |
| XA1 | 109 | Fill | Mature Harappan | X |  |
|  | 111 | Pit |  | X | X |
|  | 110 | Fill |  | X |  |
|  | 113 | Fill |  | X |  |
|  | 116 | Pit/Posthole fill |  | X |  |
|  | 119 | Pit |  | X |  |
|  | 121 | Pit |  | X |  |
|  | 120 | Collapse |  | X |  |
|  | 115 | Pit |  | X | X |
|  | 125 | Ashy fill |  | X | X |
|  | 126 | Fill |  | X |  |
|  | 128 | Fill |  | X | X |
|  | 129 | Pit |  | X | X |
|  | 130 | Pit |  | X | X |
|  | 132 | Pit |  | X | X |
|  | 134 | Ashy surface |  | X | X |
|  | 135 | Pit |  | X |  |
|  | 137 | Pit |  | X | X |
| XM2 | 314 | Pit |  | X | X |
|  | 315 | Pit |  |  | X |
|  | 316 | Pit Lining |  |  | X |
|  | 302 | Collapse |  | X | X |
|  | 303 | Collapse |  | X | X |
|  | 304 | Ashy fill |  | X | X |
|  | 305 | Fill |  | X | X |
|  | 308 | Fill |  | X |  |
|  | 310 | Fill |  | X | X |
|  | 317 | Fill |  | X | X |
|  | 319 | Pit |  | X | X |
|  | 323 | Pit |  | X | X |
|  | 321 | Fill |  | X |  |


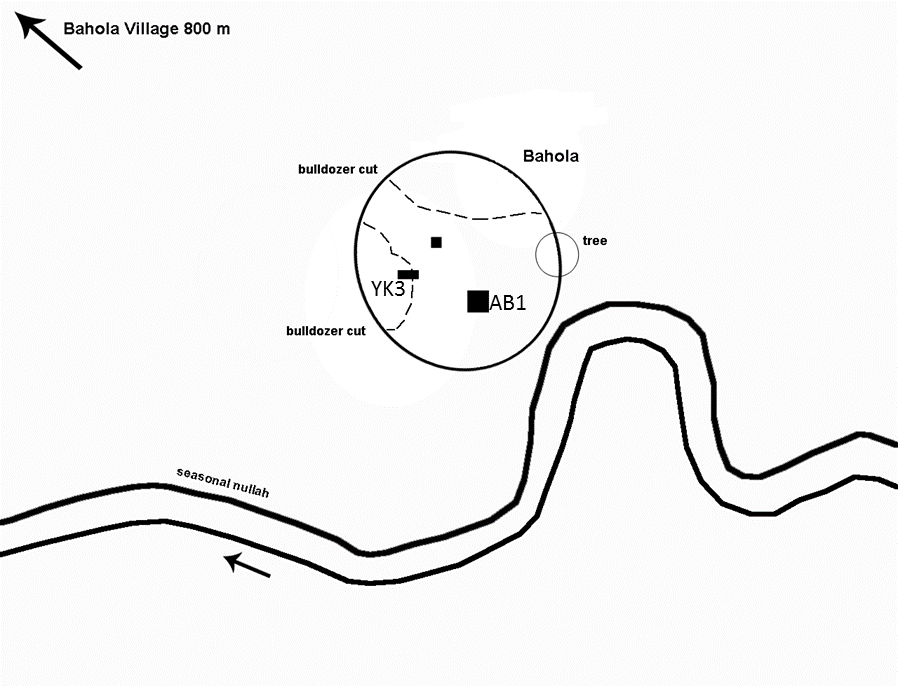


**Fig. S12** Location of trenches AB1 and YK3 at Bahola (Singh et al. 2013: Fig. 4, used with permission)


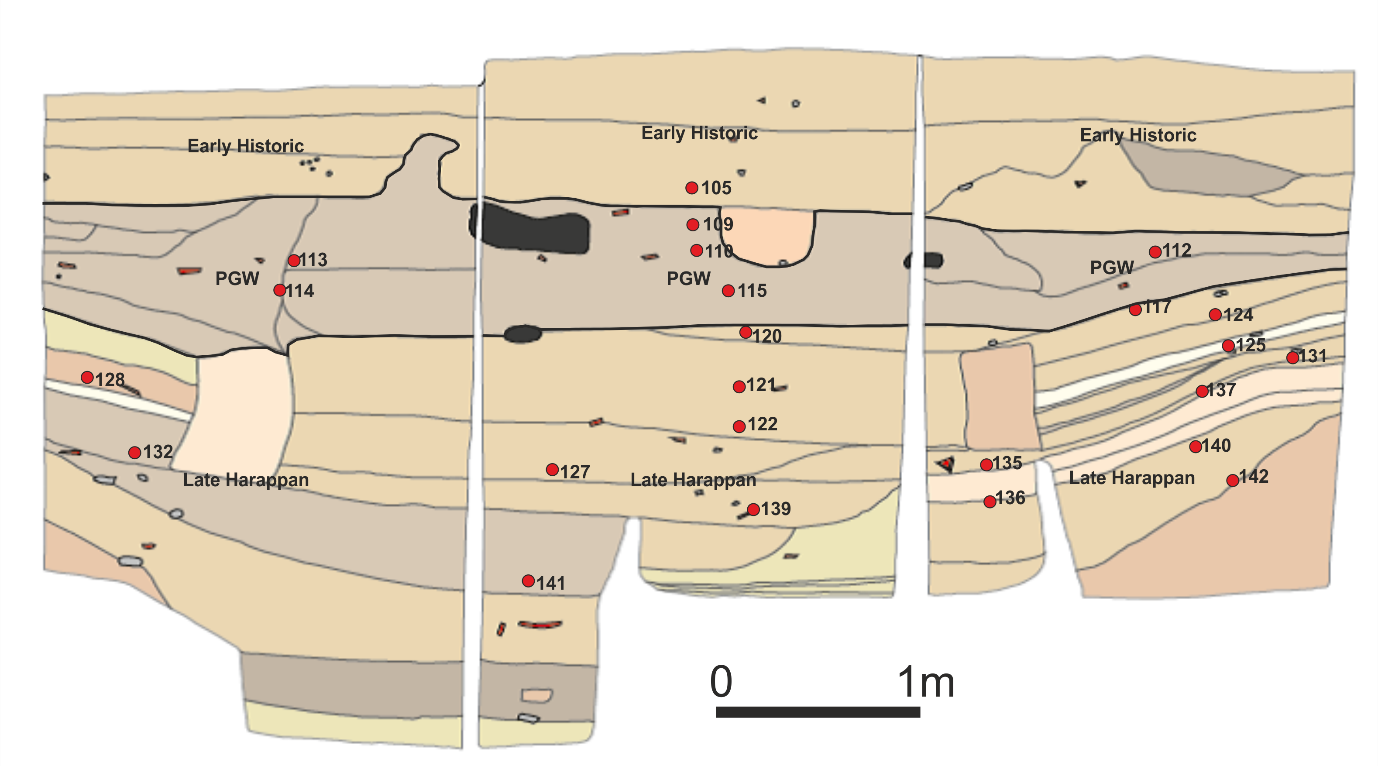


**Fig. S13** Stratigraphy of trench AB1 at Bahola including relative location of some of the samples analysed and phasing (Bates 2016, used with permission)

**Table S5** Samples analysed at Bahola (Bates 2016)

| **Trench** | **Context** | **Context Type** | **Period** | **Macro** | **Phytolith** |
| --- | --- | --- | --- | --- | --- |
| AB1 | 109 | Surface | PGW | X |  |
|  | 110 | Fill |  | X | X |
|  | 110B | Bricky patch in 110 |  |  | X |
|  | 111 | Fill |  | X |  |
|  | 112 | Structural |  | X | X |
|  | 113 | Pit |  | X | X |
|  | 115 | Surface |  | X | X |
|  | 116 | Fill |  | X | X |
|  | 117 | Structural | Late Harappan | X | X |
|  | 118 | Hearth |  |  | X |
|  | 120 | Fill | PGW | X | X |
|  | 121 | Fill |  | X | X |
|  | 122 | Fill | Late Harappan | X |  |
|  | 123 | Structural |  | X | X |
|  | 124 | Fill |  |  | X |
|  | 125 | Fill |  | X | X |
|  | 125B | Bricky in 125 |  |  | X |
|  | 126 | Fill |  | X |  |
|  | 127 | Pit |  | X | X |
|  | 128 | Pit |  | X | X |
|  | 129 | Fill |  | X | X |
|  | 131 | Fill |  | X | X |
|  | 131g | Ashy fill |  | X |  |
|  | 132 | Ashy fill |  | X | X |
|  | 133 | Fill | PGW | X | X |
|  | 134 | Structural | Late Harappan | X | X |
|  | 134B | Structural |  |  | X |
|  | 135 | Pit |  | X | X |
|  | 136 | Pit |  | X | X |
|  | 137 | Fill |  | X | X |
|  | 138 | Fill |  | X | X |
|  | 139 | Pit |  | X |  |
|  | 140 | Fill |  | X | X |
|  | 141 | Fill |  | X | X |
|  | 143 | Pit |  | X | X |
|  | 143L | Pit lining (external) | Late Harappan |  | X |
