# Supplementary material for: Exploring Indus crop processing: combining phytolith and macrobotanical analyses to consider the organisation of agriculture in northwest India c. 3200–1500 bc
Source: Veg Hist Archaeobot. 2016 May 21;26(1):25–41. doi: 10.1007/s00334-016-0576-9 (PMC7346983; doi:10.1007/s00334-016-0576-9)
Supplement: Supplementary file 2 — Supplementary material 2 (DOCX 1138 kb) [file 334_2016_576_MOESM2_ESM.docx]

**Exploring Indus crop processing: combining phytoliths and macrobotanical analysis to consider the organisation of agriculture in northwest India c. 3200-1500 BC**

Jennifer Bates^1^, Ravindra Nath Singh^2^, Cameron A. Petrie^1^

*^1^ Division of Archaeology, University of Cambridge, Downing Street, Cambridge, CB2 3DZ, UK, e-mail: jb599@cam.ac.uk*

*^2^ Department of AIHC and Archaeology, Banaras Hindu University, Varanasi – 221005, India*

Supplementary Information 2: Crop Processing Models

I Introduction

In order to explore the data presented in this paper, models of crop processing products and by-products were needed. As noted in the paper, these were developed from Hillman (1981, 1984) and Stevens (2003) for free-threshing wheat and hulled barley, Reddy (1994, 1997, 2003), Harvey (2006), Harvey and Fuller (2005) and Weisskopf (2010) for hulled millets and Thompson (1996), Harvey (2006) and Weisskopf (2010) for rice. This supplementary information outlines these models in more detail, showing how the two proxies, macrobotanical remains and phytoliths, were combined.

II Wheat

Hillman’s (1981, 1984) models focused on wheat and barley, and he noted the differences in hulled and naked grains. Hulled grains require extra processing as the chaff (glumes, lemma, palea) are tough and adhere to the grain tightly, while in free-threshing grains these elements are brittle and break apart quickly, leaving a free grain. Hulled grains therefore need an extra processing stage to break the chaff from the grain, which in turns affects the assemblage content throughout the processing.

Fuller (2002) and Fuller and Madella (2002) have argued that in the Indus Civilisation, free-threshing wheats dominated, such as *Triticum durum/aestivum* or *Triticum sphaerococcum*. No obviously hulled wheat varieties were noted in the *Land, Water, Settlement Project* samples and those grains that could be identified to species level were identified as *Triticum durum/aestivum* (Bates 2015). As such, a free-threshing model will be explored here (Fig. SII.1).

The first stage in any processing is harvest. Different harvest techniques are needed for different plants. For free-threshing wheats Hillman (1981, 1984) suggests that reaping with a sickle or uprooting are most common. These methods would result in an assemblage incorporating both ear, stem and leaf, and weeds (Hillman 1981, 1984). The next step is to break the ear and chaff from the straw and to free the grains from the straw (Hillman 1981, 1984). This is done through threshing. Threshing can be carried out by flail or trampling (Hillman 1981, 1984).

Following threshing there is raking (Hillman 1981, 1984), which divides the grain and light chaff (glumes, lemma, palea) from the culm, leaf and rachis internode fragments. In free-threshing wheat the glumes are fragile and break easily, releasing the grain, but the rachises are tough and remain attached to one another and often to the culm. They are therefore removed through raking, which removes the larger elements from the smaller ones like grains, glumes and weeds. During threshing, however, some of the rachis fragments can break from the culm (the basal rachis internode being the weakest point) and as such some rachis fragments can continue into the later stages. Winnowing is then used to remove the lighter chaff and weeds from the heavier grains and weeds (Hillman 1981, 1984). Some rachises if light enough can also be removed while the grain, most weeds and some rachis fragments remain.

Following these stages, the bulk of the leaf, culm and glume have been removed, as have many of the rachis fragments. The remaining contaminants are the weeds and removal of these is done through sieving, relying on the size of the weed to remove it (Hillman 1981, 1984; Stevens 2003). Two stages of sieving are needed: the first is coarse sieving to remove seeds larger than the grain and any remaining rachis fragments, and the second is fine sieving to remove those weeds smaller than the grain (Hillman 1981, 1984; Stevens 2003).

The final stage of wheat processing is hand-sorting to remove those weeds that are the same size as the grain (Stevens 2003), and any remaining contaminants that have accidentally managed to slip through the earlier stages.

Hillman’s (1981, 1984) model suggests that storage occurs after the fine sieving stage, and that further fine sieving is carried out after storage but before hand-sorting. However, as suggested in Stevens (1996, 2003) the point of storage is not fixed and can occur at any point in the process. As such it has not been placed on the model in Fig. SII.1. It will also not be placed on the barley, millet, rice or pulse processing models for similar reasons.

Free-threshing wheat chaff is rare on sites (Ballantyne pers. comm.) as it is often used for other purposes such as fodder or fuel. This is, however in reference to European sites, and may not apply to Indus sites, but it is an important point to bear in mind. Similarly, although this model has described rachis internode fragments as being attached to one another, in charring they often break into individual rachis units (Ballentyne pers. comm.), which is another issue that has to be remembered, and which also applies to hulled barley.


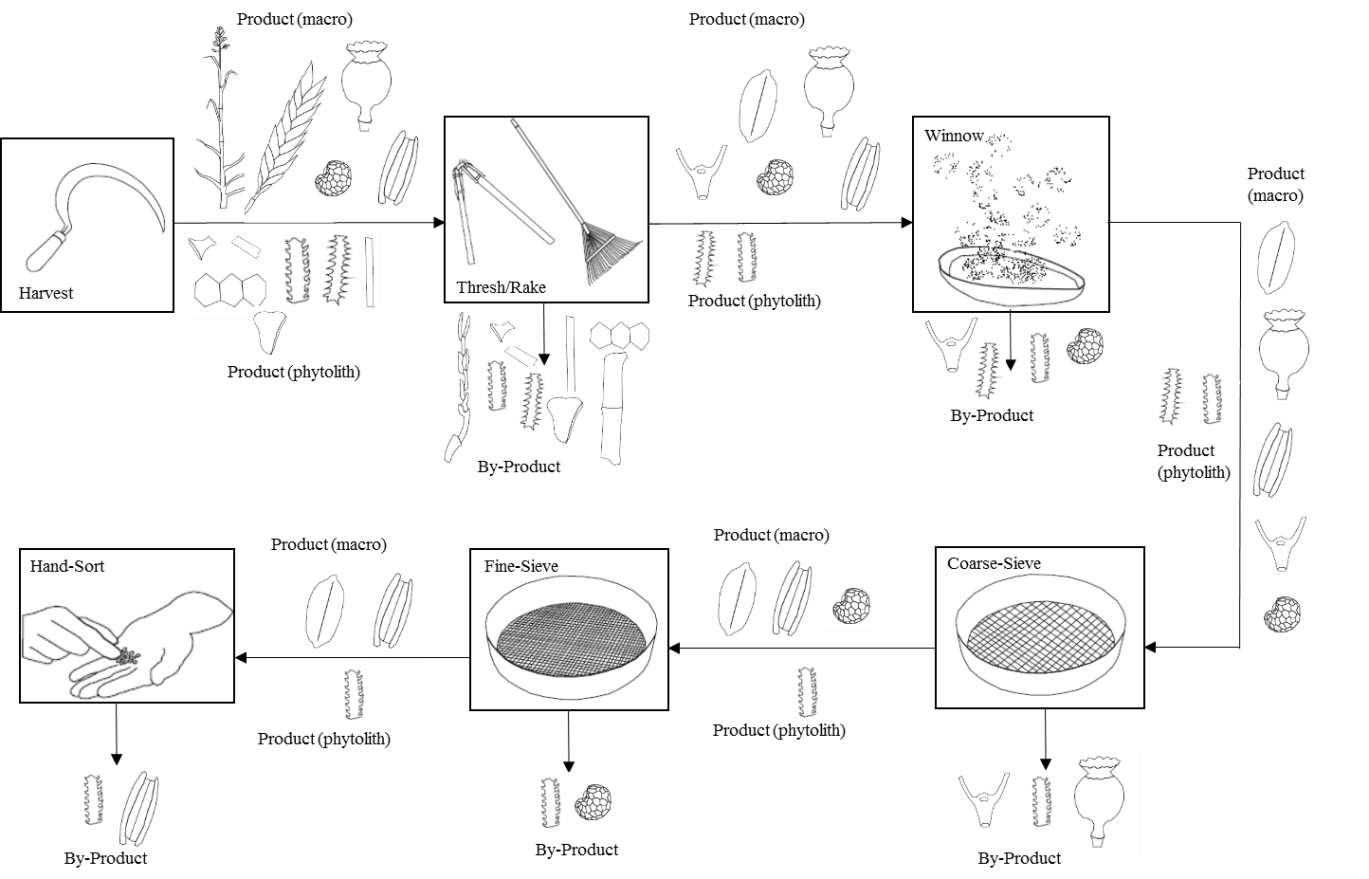


**Fig. S1** Wheat processing stages (After Hillman 1984).

III Barley

The type of barley exploited by Indus Civilisation populations has also been debated (Fuller 2002; Fuller and Madella 2002). Fuller (2002) and Fuller and Madella (2002) have argued that the majority of barley at Indus Civilisation settlements is hulled 6-row barley. However, this is not supported in the literature review in which a range of barley types were noted. The majority of the grains present at the sites that could be identified to this level of specificity were hulled (Bates 2015). A hulled barley model is therefore presented.

Hillman (1981, 1984) noted that hulled barley processing is similar to wheat processing, but with extra stages added in: including hummeling, which breaks the awns and tips of the chaff off from the grain, and is added before winnowing, after raking (Hillman 1981, 1984), and milling/pounding to remove the chaff before a final winnowing, after all the other stages are completed (Hillman 1981, 1984). The nature of the barley ear will make the parts present at each stage different from wheat. The processing stages are shown in Fig. SII.2.

Like free-threshing wheat, the first stages are harvest, threshing and raking. These produce similar products and by-products. The next stage differs though as hummeling is carried out to break the awns from the palea and lemma. This, combined with the different light chaff parts and their nature on barley, therefore changes the by-products of the next stage, the winnowing. The barley grain remains invested in the lemma and palea even when the awns are broken off (Hillman 1981, 1984). The awns can survive well in archaeological contexts as they are often tough (Ballantyne pers. comm.).

The next stages, coarse and fine sieving and the hand sorting, are the same, as with free-threshing wheat, with the exception that the barley grains remain in their lemma and palea protection. To remove these two extra stages are needed once the grain is cleaned of weeds: milling or pounding to break the chaff from the grain and winnowing to remove this chaff (Hillman 1981, 1984).


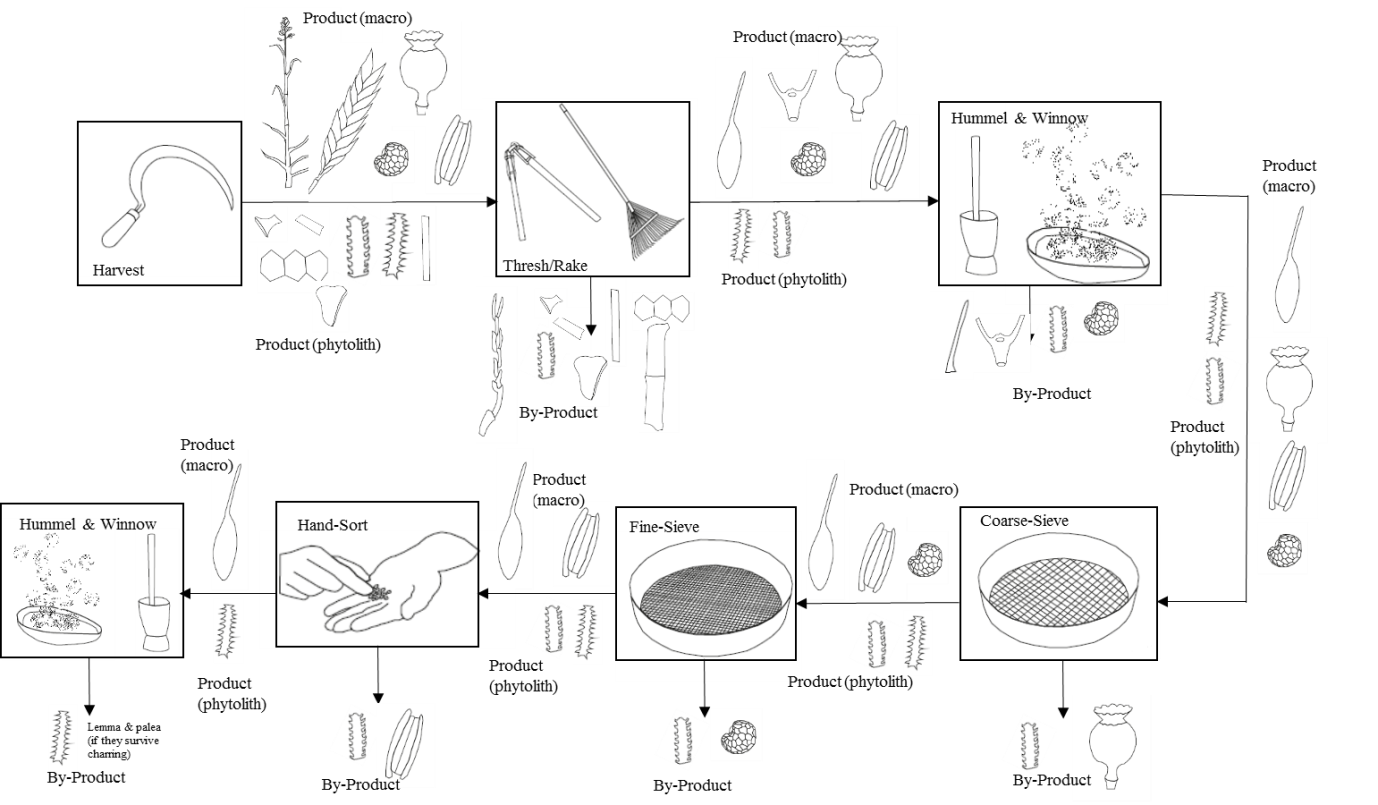


**Fig. S2** Hulled barley processing stages. (After Hillman 1984).

IV Millets

The seminal study for millet processing was carried out by Reddy (1994, 1997, 2003) using ethnographic analysis form India. Reddy (1994, 1997, 2003) developed and applied millet processing models to archaeological remains from Oriyo Timbo and Babar Kot. More recently these models have been adapted by Harvey (2006), Harvey and Fuller (2005) and Weisskopf (2010) to include phytoliths as well as macrobotanical remains.

Before outlining the models, an important distinction has to be made: between hulled and naked millets (Reddy 1994, 1997, 2003; Fuller 2002). This is because it affects the stages needed to process the grains and therefore the archaeological assemblages (Reddy 1994, 1997, 2003). Looking at the literature review from Chapter 4, it can be seen that while both hulled millets such as *Setaria* sp. and free-threshing millets like *Eleusine* sp. are present at Indus Civilisation sites, it is hulled millets that predominate in the eastern Harappan region. These are *Echinochloa sp*., *Panicum sp*., *Pennisetum sp*., and *Setaria sp*. *Echinochloa colona*, *Setaria* cf. *pumila* and *Panicum* sp. were identified in the *Land, Water, Settlement Project* samples and as such only a hulled processing model will be presented here.

The first stage is harvest. The millets discussed above all fall into Reddy’s (1994, 1997, 2003) ‘Type B’ harvesting category. This refers to the way the millets are harvested: Type B millets are those with slender stalks which are therefore usually harvested at the base of the stem, rather than taking just the panicle as in the thick stemmed Type A millets (Reddy 1994, 1997, 2003). Cutting these millets at the base therefore incorporates not only stems but also a wide variety of weed as well (Reddy 1994, 1997, 2003). Threshing divides the panicles from the leaf and culm and can be done several ways: beating with a stick, using feet, or getting cattle to trample it (Reddy 1994, 1997, 2003). This depends on the grain maturity and quantity (Reddy 1994, 1997, 2003), and cattle trampling is not usually used on small grained millets (Reddy 1994, 1997, 2003). The threshed crop is then sorted, either by raking or by using hands like combs to pull out the bigger by-products of culm and leaf (Reddy 1994, 1997, 2003).

Following this, the smaller elements of the spikelets and weeds are left, and these are winnowed to remove light weeds and separated chaff like the rachilla. In South Asia this is often carried out using specially designed winnowing baskets (Lundström-Baudais et al. 2002). This stage is very time consuming because of the repetition needed (Weisskopf 2010).

Following the first winnowing stage, the grains are still in spikelets, the hull of the glumes, lemma and palea still adhering to the grain. Parching is common to make these elements more brittle before they are removed (Reddy 1994, 1997, 2003). This parching is commonly done at settlements and introduces the potential for charring.

The final stages are to pound the grain to break up the glumes, and to winnow and hand-pick to remove the chaff and remaining heavy weeds (Reddy 1994, 1997, 2003) (Fig. SII.3).


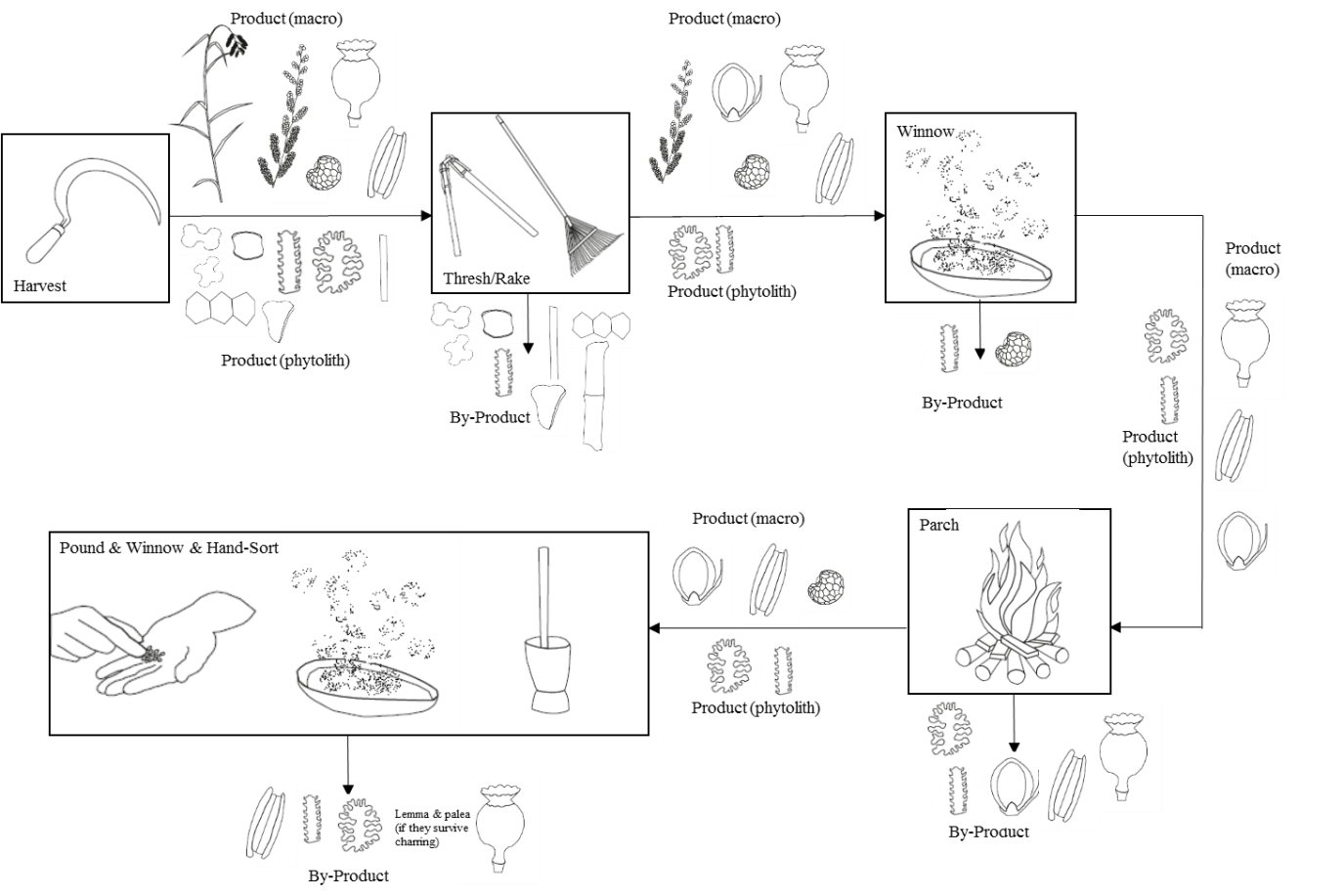


**Fig. S3** Small, hulled millet processing stages. (After Reddy 1994, 1997, 2003).

V Rice

Thompson (1996) carried out ethnographic analysis of rice processing in Thailand, and his model has become the basis for rice crop processing analysis in archaeology. Harvey (2006) and Weisskopf (2010) have subsequently adapted this to incorporate phytoliths. What follows is an adapted version of these three models.

The first stage in rice processing is harvest. Thompson (1996) observed three main methods for this: by sickle, by uprooting or by use of finger-knives. Each method affects the assemblage, as can be seen in Fig. SII.4. Harvey (2006) has argued that finger-knife harvesting is rare in India, and as such it has not been included in this model. Similarly, while Thompson (1996) suggested that rice growing produces fewer weeds than other cereals, it has been observed that dry-cropping, which would include *Oryza nivara* or *O. indica*, produces more weeds than paddy fields, as the seeds are broadcast sown rather than transplanted (Moody 1989; Thompson 1996). As such, using both the uprooting and sickle method would include a significant number of weeds. Both sickle harvesting and uprooting produce the same assemblage: panicles with straw and weeds, and as such have been combined to simplify Fig. SII.4.

The next stage is threshing and raking to remove the straw from the spikelets. Weisskopf (2010) suggests that threshing can be done in four ways: through cattle trampling, through using a stone roller, by hitting it with flails, or by dragging the panicles through wooden combs. These methods break the panicle into spikelets away from the leaf and culm and which then need to be divided from the light chaff. Like millets, this is usually done through basket winnowing rather than sieving (Reddy 1994, 1997, 2003). Winnowing is then carried out to remove the light weeds (Thompson 1996; Weisskopf 2010).

From this point on the processing becomes more time consuming (Thompson 1996; Harvey 2006; Weisskopf 2010). After winnowing the spikelets need to be broken up and the grain separated from the palea and lemma and spikelet bases, and from the heavy weeds. Unlike millet, rice is rarely parched in India (Harvey 2006) and instead is left to sun dry before being pounded or milled. This is then winnowed again and finally hand sorted (Fig. SII.4).


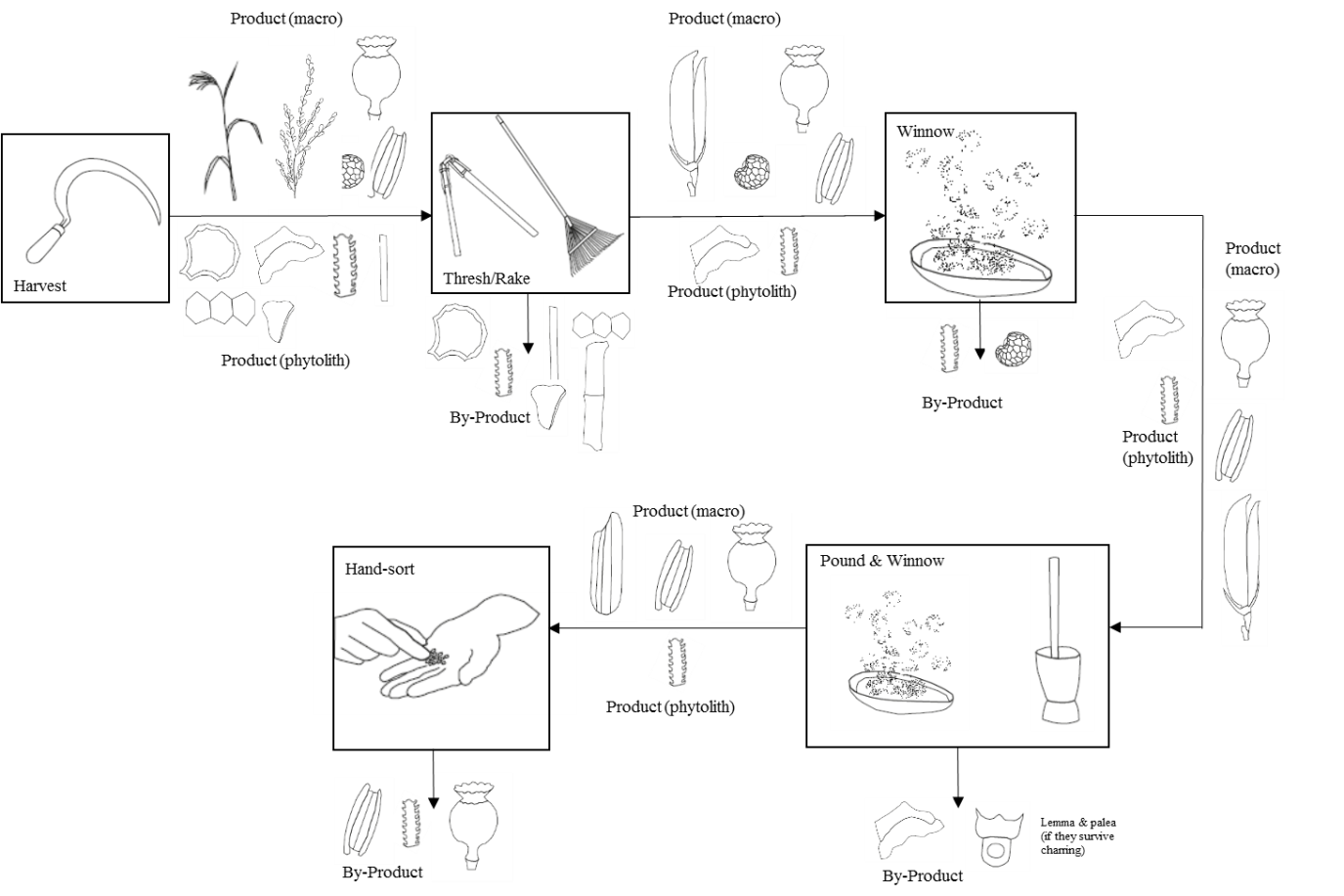


**Fig. S4** Rice processing stages (after Thompson 1996; Harvey 2006; Weisskopf 2010)

References

Bates J (2015) Social organisation and change in Bronze Age South Asia: a multi-proxy approach to urbanisation, deurbanisation and village life through phytolith and macrobotanical analysis. Dissertation, University of Cambridge

Fuller DQ (2002) Fifty years of archaeobotanical studies in India: laying a solid foundation. In: Settar S, Korisettar R (eds) Indian archaeology in retrospect III: archaeology and interactive disciplines. Manohar, New Delhi, pp 247-364

Fuller DQ, Madella M (2002) Issues in Harappan archaeobotany: retrospect and prospect. In: Settar S, Korisettar R (eds) Indian archaeology in retrospect II: protohistory. Manohar, New Delhi, pp 317-390

Harvey EL (2006) Early agricultural communities in Northern and Eastern India: an archaeobotanical investigation. Dissertation, University College London

Harvey EL, Fuller DQ (2005) Investigating crop processing using phytolith analysis: the example of rice and millets. J Archaeol Sci 32:739-752. doi:10.1016/j.jas.2004.12.010

Hillman GC (1981) Reconstructing crop husbandry practices from charred remains of crops. In: Mercer R (ed) Farming practice in British prehistory. Edinburgh University Press, Edinburgh, pp 123-161

Hillman GC (1984) Interpretation of archaeological plant remains: the application of ethnographic models from Turkey. In: Van Zeist W, Casparie WA (eds) Plants and ancient man, studies in palaeoethnobotany. Balkema, Rotterdam

Lundström-Baudais K, Rachoud-Schneider AM, Baudais D, Poissonnier B (2002) Le broyage dans la chaine de transformation du millet (*Panicum miliaceum*): outils, gestes et ecofacts. In: Procopiou H, Treuil R (eds) Moudre et broyer, vol. 1, méthodes. Comité des Travaux Historiques et Scientifiques, Paris, pp 155-180

Moody K (1989) Weeds of rice reported in rice in South and Southeast Asia. International Rice Research Institute, Manila

Reddy SN (1994) Plant usage and subsistence modelling: an ethnoarchaeological approach to the Late Harappan of northwest India. Dissertation, University of Wisconsin-Madison

Reddy SN (1997) If the threshing floor could speak: integration of agriculture and pastoralism during the Late Harappan in Gujarat, India. J Anthropol Archaeol 16:162-187. doi:10.1006/jaar.1997.0308

Reddy SN (2003) Discerning palates of the past: an ethnoarchaeological study of crop cultivation and plant usage in India. (Ethnoarchaeological series 5) International Monographs in Prehistory, Ann Arbor

Stevens CJ (1996) Iron Age and Roman agriculture in the Upper Thames Valley: archaeobotanical and social perspectives. Dissertation, University of Cambridge

Stevens CJ (2003) An investigation of agricultural consumption and production models for prehistoric and Roman Britain. Environ Archaeol 8:61-76. Doi:10.1179/env.2003.8.1.61

Thompson GB (1996) Ethnographic models for interpreting rice remains. In: Higham C, Thosarat R (eds) The excavations at Khok Phanom Di, a prehistoric site in Central Thailand. The Society of Antiquaries of London, London, pp 119-150

Weisskopf A (2010) Vegetation, agriculture and social change in Late Neolithic China: a phytolith study. Dissertation, University College London
