# Supplementary material for: Exploring Indus crop processing: combining phytolith and macrobotanical analyses to consider the organisation of agriculture in northwest India c. 3200–1500 bc
Source: Veg Hist Archaeobot. 2016 May 21;26(1):25–41. doi: 10.1007/s00334-016-0576-9 (PMC7346983; doi:10.1007/s00334-016-0576-9)
Supplement: Supplementary file 3 — Supplementary material 3 (DOCX 9311 kb) [file 334_2016_576_MOESM3_ESM.docx]

**Exploring Indus crop processing: combining phytoliths and macrobotanical analysis to consider the organisation of agriculture in northwest India c. 3200-1500 BC**

Jennifer Bates^1^, Ravindra Nath Singh^2^, Cameron A. Petrie^1^

*^1^ Division of Archaeology, University of Cambridge, Downing Street, Cambridge, CB2 3DZ, UK, e-mail: jb599@cam.ac.uk*

*^2^ Department of AIHC and Archaeology, Banaras Hindu University, Varanasi – 221005, India*

Supplementary Information 3: Raw Data

**I Macrobotanical Remains**

**Table S1** Context-by-context raw counts of cereals at Dabli vas Chugta. Only MNI counts given. (Bates 2016)

| Context | 204 | 205 | 206 | 207 | 223 | 224 | 225 | 226 | 227 | 229 | 230 | 231 | 233 | 234 | 235 | 236 | 238 | 239 | 240 | 241 | 242 | 243 | 244 | 245 | 248 | 249 | 250 | 251 | 252 | 253 | 254 | 255A | 255B | 255C | 256 | 257 | 258 | 259 |
| --- | --- | --- | --- | --- | --- | --- | --- | --- | --- | --- | --- | --- | --- | --- | --- | --- | --- | --- | --- | --- | --- | --- | --- | --- | --- | --- | --- | --- | --- | --- | --- | --- | --- | --- | --- | --- | --- | --- |
| Litres | 40 | 20 | 40 | 40 | 15 | 20 | 20 | 20 | 20 | 20 | 20 | 20 | 20 | 20 | 20 | 20 | 20 | 20 | 20 | 20 | 20 | 20 | 20 | 20 | 20 | 15 | 20 | 20 | 20 | 20 | 20 | 10 | 15 | 5 | 15 | 20 | 20 | 20 |
| Hordeum vulgare |  |  | 1 | 1 |  |  |  |  |  |  |  |  |  |  |  |  |  |  |  |  |  |  |  | 1 |  | 1 | 2 |  |  | 1 |  | 1 |  | 2 | 2 | 2 | 22 | 1 |
| Triticum sp. |  |  |  |  |  |  |  |  |  |  |  |  |  |  |  |  |  |  |  |  |  |  |  |  |  |  |  |  |  |  |  |  |  |  |  |  | 1 |  |
| F-T Triticum rachis |  |  |  |  |  |  |  |  |  |  |  |  |  |  |  |  |  |  |  |  |  |  |  |  |  |  |  |  |  |  |  |  |  |  | 1 |  |  |  |
| Hordeum/Triticum |  |  | 1 |  |  |  |  |  |  |  |  |  |  |  |  |  |  |  |  |  |  |  |  |  |  |  |  | 2 |  |  |  | 4 | 1 |  | 1 | 1 | 5 | 1 |
| Setaria sp. |  |  |  |  |  |  |  |  |  |  |  |  |  |  |  |  |  |  |  |  |  |  |  |  |  |  |  |  |  |  |  |  |  |  |  | 1 |  |  |
| Panicum sp. |  |  |  |  |  |  |  |  |  |  |  |  |  |  |  |  |  |  |  |  |  |  |  |  |  |  |  |  | 1 |  |  |  |  |  |  |  |  |  |
| SEB |  | 1 | 1 | 1 |  |  |  |  |  |  |  |  |  |  |  |  |  |  |  |  |  |  |  | 1 |  |  |  |  |  |  | 1 |  |  |  |  |  | 2 |  |
| Indet. millet |  |  |  |  |  |  |  |  |  |  |  |  |  |  |  |  |  |  |  |  |  |  |  |  |  |  |  |  |  |  |  |  |  |  |  |  | 23 | 1 |
| Domesticated Oryza spikelet base |  |  |  |  |  |  |  |  |  |  |  |  |  |  |  |  |  |  |  | 1 |  |  |  |  |  |  |  |  |  |  |  |  |  |  |  |  |  |  |

**Table S2** Context-by-context raw counts of weeds at Dabli vas Chugta. Only MNI counts given. (Bates 2016)

| Context | 204 | 205 | 206 | 207 | 223 | 224 | 225 | 226 | 227 | 229 | 230 | 231 | 233 | 234 | 235 | 236 | 238 | 239 | 240 | 241 | 242 | 243 | 244 | 245 | 248 | 249 | 250 | 251 | 252 | 253 | 254 | 255A | 255B | 255C | 256 | 257 | 258 | 259 |
| --- | --- | --- | --- | --- | --- | --- | --- | --- | --- | --- | --- | --- | --- | --- | --- | --- | --- | --- | --- | --- | --- | --- | --- | --- | --- | --- | --- | --- | --- | --- | --- | --- | --- | --- | --- | --- | --- | --- |
| Litres | 40 | 20 | 40 | 40 | 15 | 20 | 20 | 20 | 20 | 20 | 20 | 20 | 20 | 20 | 20 | 20 | 20 | 20 | 20 | 20 | 20 | 20 | 20 | 20 | 20 | 15 | 20 | 20 | 20 | 20 | 20 | 10 | 15 | 5 | 15 | 20 | 20 | 20 |
| Trianthema triquetra |  |  |  |  |  |  |  |  |  |  |  |  |  |  | 1 |  |  |  |  | 1 | 1 |  |  |  | 5 |  |  |  |  | 1 | 2 |  |  |  |  |  |  |  |
| Stellaria sp. |  |  |  |  |  |  |  |  | 1 |  |  |  |  |  |  |  |  |  |  |  |  |  |  |  |  |  |  |  |  |  |  |  |  |  |  |  |  |  |
| Chenopdium album |  |  |  |  |  |  |  |  |  |  |  |  |  |  |  |  |  |  |  |  |  |  |  |  |  |  |  |  |  |  |  |  |  |  |  |  |  | 1 |
| Eleocharis sp. |  |  |  |  |  |  |  |  | 1 |  |  |  |  |  |  |  |  |  |  |  |  |  |  |  |  |  | 3 |  |  |  | 1 |  |  |  | 1 | 3 | 9 |  |
| Cyperaceae |  |  |  |  |  |  |  |  |  |  |  |  |  |  |  |  |  |  |  |  |  |  |  |  |  |  |  |  |  |  |  |  |  |  |  |  | 1 |  |
| (all) Fabaceae |  |  |  |  |  |  |  |  |  |  |  |  |  |  |  |  |  |  |  |  |  | 1 |  |  |  |  |  |  |  |  |  | 1 |  |  |  |  |  |  |
| Avena sp. |  |  |  |  |  |  |  |  |  |  |  |  |  |  |  |  |  |  |  |  |  |  |  |  |  |  |  |  |  |  |  |  |  |  |  |  | 2 |  |
| Chrysopogon sp. |  |  |  |  |  |  |  |  |  |  |  |  |  |  |  |  |  |  |  |  |  |  |  |  |  |  |  |  |  |  |  |  |  |  |  |  | 6 |  |
| Eragrostis sp. |  |  |  |  |  |  |  |  |  |  |  |  |  |  |  |  |  |  |  |  |  |  |  |  |  |  | 1 |  |  |  |  |  |  |  |  |  |  |  |
| Grass 1 |  |  |  | 2 |  |  |  |  |  |  |  |  |  |  |  |  |  |  |  |  |  |  |  |  |  |  |  |  |  |  |  |  |  | 1 |  |  | 1 |  |
| Indet. grass |  |  |  | 3 |  |  |  |  |  |  |  |  |  |  |  | 1 |  |  |  |  |  |  |  |  |  |  | 2 |  | 1 |  |  | 1 |  | 1 | 1 | 2 | 10 | 4 |
| Polygonum sp. |  |  |  |  |  |  |  |  |  |  |  |  |  |  |  |  |  |  |  |  |  |  |  |  |  |  |  |  |  |  |  |  |  |  |  |  |  |  |
| Curled embryo |  |  |  |  |  |  |  |  |  |  |  |  |  |  |  |  |  |  |  |  |  |  |  |  |  |  |  |  |  |  | 1 |  |  |  | 2 | 1 |  |  |
| Indet. round weed |  | 1 | 1 |  | 2 |  |  |  |  |  |  | 20 |  |  |  |  |  |  |  |  |  |  |  |  |  |  |  |  | 3 |  | 22 | 1 |  | 1 | 2 | 2 | 7 |  |

**Table S3** Context-by-context raw counts of crops at Burj. Only MNI counts given. (Bates 2016)

| Context | 140 | 141 | 142 | 145 | 146 | 147 | 148 | 150 | 151 | 152 | 156 | 157 | 158 | 162 | 209 | 210 | 213 | 216 | 219 | 220 |
| --- | --- | --- | --- | --- | --- | --- | --- | --- | --- | --- | --- | --- | --- | --- | --- | --- | --- | --- | --- | --- |
| Litres | 20 | 20 | 20 | 20 | 20 | 20 | 20 | 20 | 20 | 20 | 20 | 20 | 20 | 20 | 20 | 20 | 20 | 20 | 20 | 20 |
| Period | PGW | PGW | PGW | PGW | PGW | PGW | PGW | PGW | PGW | PGW | PGW | PGW | PGW | PGW | EH | EH | EH | EH | EH | EH |
| Hordeum vulgare | 2 | 2 | 6 | 1 |  |  |  | 1 | 8 | 1 | 2 |  |  |  |  |  |  |  | 1 |  |
| Triticum sp. |  |  |  |  |  |  |  |  |  |  | 1 |  |  |  |  |  |  |  |  |  |
| Hordeum/Triticum |  | 2 | 7 | 1 |  | 1 |  | 1 | 1 | 1 | 1 |  |  |  |  |  | 2 |  |  | 2 |
| Large cereal rachis |  |  |  |  |  |  |  |  | 1 |  |  |  |  |  |  |  |  |  |  |  |
| Echinochloa sp. |  | 5 | 105 | 3 |  |  | 1 | 6 | 10 |  |  | 2 | 49 |  |  |  |  |  |  |  |
| Setaria sp. |  |  | 7 |  |  |  |  | 10 | 27 | 1 | 2 |  | 51 |  |  |  |  |  |  |  |
| Panicum sp. |  |  | 4 |  |  |  |  | 1 |  |  |  |  | 8 |  |  |  |  |  |  |  |
| SEB |  |  | 13 |  |  |  |  |  | 3 |  |  |  | 8 |  |  |  |  |  |  |  |
| Indet. millet |  |  |  |  |  | 1 |  | 3 | 7 |  |  |  | 17 | 1 |  |  |  |  |  |  |

**Table S4** Context-by-context raw counts of weeds at Burj. Only MNI counts given. (Bates 2016)

| Context | 140 | 141 | 142 | 145 | 146 | 147 | 148 | 150 | 151 | 152 | 156 | 157 | 158 | 162 | 209 | 210 | 213 | 216 | 219 | 220 |
| --- | --- | --- | --- | --- | --- | --- | --- | --- | --- | --- | --- | --- | --- | --- | --- | --- | --- | --- | --- | --- |
| Litres | 20 | 20 | 20 | 20 | 20 | 20 | 20 | 20 | 20 | 20 | 20 | 20 | 20 | 20 | 20 | 20 | 20 | 20 | 20 | 20 |
| Period | PGW | PGW | PGW | PGW | PGW | PGW | PGW | PGW | PGW | PGW | PGW | PGW | PGW | PGW | EH | EH | EH | EH | EH | EH |
| Stellaria sp. |  | 2 | 3 | 1 | 1 |  | 1 | 1 | 3 |  | 2 |  |  | 2 |  |  |  |  |  |  |
| Scirpus sp. |  |  |  |  |  |  |  | 1 |  |  |  |  | 1 |  |  |  |  |  |  |  |
| Cyperaceae |  |  |  |  |  |  |  |  |  |  |  |  | 9 |  |  |  |  |  |  |  |
| (all) Fabaceae |  |  |  |  |  |  |  |  | 3 |  |  |  |  |  |  |  |  |  |  |  |
| Fumaria sp. |  |  |  |  |  |  |  |  |  |  |  |  | 1 |  |  |  |  |  |  |  |
| Chrysopogon sp. |  |  |  |  |  |  |  | 1 |  |  |  |  |  |  |  |  |  |  |  |  |
| Echinochloa crus-galli |  |  | 2 |  |  |  |  |  |  |  |  |  |  |  |  |  |  |  |  |  |
| Pennisetum sp. |  |  |  |  |  |  |  |  | 2 |  |  |  |  |  |  |  |  |  |  |  |
| Indet. Grass |  |  |  |  |  |  |  | 2 | 1 |  | 1 |  | 5 |  |  | 2 |  |  |  |  |
| Indet. round weed |  |  | 1 |  |  |  |  | 1 | 1 |  |  | 1 | 1 |  |  |  |  |  |  |  |

**Table S5** Context-by-context raw counts of cereals at Masudpur VII. Only MNI counts given (Bates 2016)

| Context | 508 | 515 | 517 | 406 | 407 | 410 | 409 | 414 | 415 | 418 | 419 | 422 | 422 burning | 513 | 514 | 423 | 425 | 428 | 429 | 426 | 430 | 522 | 525 | 526 | 527 |
| --- | --- | --- | --- | --- | --- | --- | --- | --- | --- | --- | --- | --- | --- | --- | --- | --- | --- | --- | --- | --- | --- | --- | --- | --- | --- |
| Litres | 20 | 20 | 20 | 20 | 20 | 20 | 20 | 10 | 5 | 20 | 20 | 20 | 20 | 20 | 20 | 20 | 20 | 20 | 20 | 10 | 20 | 20 | 20 | 20 | 20 |
| Period | LH | LH | LH | MH | MH | MH | MH | MH | MH | MH | MH | MH | MH | MH | MH | EH | EH | EH | EH | EH | EH | EH | EH | EH | EH |
| Hordeum vulgare |  | 1 |  |  |  |  |  |  |  |  |  |  |  | 1 |  |  |  | 17 | 3 |  | 4 | 1 |  |  |  |
| Triticum sp. |  |  |  |  |  |  |  |  | 3 |  |  |  |  |  |  | 1 |  | 1 |  |  |  |  |  |  |  |
| Hordeum/Triticum | 1 |  | 8 |  |  |  |  |  | 4 |  | 1 |  |  | 1 |  |  |  |  | 1 |  | 6 |  |  |  |  |
| Oryza sp. |  | 3 | 4 |  |  |  |  |  |  |  |  |  |  |  |  |  |  | 1 | 1 |  |  |  |  |  |  |
| Domest. Oryza spikelet base |  | 2 |  |  |  |  |  |  |  |  |  |  |  |  |  |  |  |  | 1 |  |  |  |  |  |  |
| Wild Oryza spikelet base |  |  |  |  |  |  |  |  |  |  |  |  |  | 135 |  |  |  |  |  |  |  |  |  |  |  |
| Immature Oryza spikelet base |  | 3 |  |  |  |  |  |  |  |  |  |  |  | 17 |  |  |  |  |  |  |  |  |  |  |  |
| Indet. Oryza spikelet base |  | 2 |  |  |  |  |  |  |  |  |  |  |  | 26 |  |  |  |  |  |  |  |  |  |  |  |
| Silicified Oryza spikelet base |  | 1 |  |  |  |  |  |  |  |  |  |  |  | 1 |  |  |  |  |  |  |  |  |  |  |  |
| Oryza husk fragment | 1 | 1 |  |  |  |  |  |  |  |  |  |  |  |  | 1 |  |  |  |  |  |  |  |  |  |  |
| Echinochloa sp. |  | 7 |  |  |  |  |  |  |  |  | 1 |  | 1 | 3 | 3 | 3 | 2 |  | 8 |  | 3 | 2 | 9 | 2 |  |
| Setaria sp. |  |  |  |  |  |  |  |  |  |  |  |  |  |  |  | 4 |  |  | 5 |  | 1 |  |  | 1 |  |
| Panicum sp. |  | 1 |  |  |  |  |  |  |  |  |  |  |  |  |  | 1 |  |  | 2 |  | 2 |  |  | 2 |  |
| SEB |  |  |  |  |  |  |  |  |  | 1 |  |  | 1 |  |  |  | 1 |  | 1 |  |  |  | 5 | 1 |  |
| Indet. millet |  | 12 |  |  |  |  |  |  |  |  |  |  |  |  | 3 | 2 |  |  |  |  | 3 |  | 4 | 1 | 2 |

**Table S6** Context-by-context raw counts of weeds at Masudpur VII. Only MNI counts given (Bates 2016)

| Context | 508 | 515 | 517 | 406 | 407 | 410 | 409 | 414 | 415 | 418 | 419 | 422 | 422 burning | 513 | 514 | 423 | 425 | 428 | 429 | 426 | 430 | 522 | 525 | 526 | 527 |
| --- | --- | --- | --- | --- | --- | --- | --- | --- | --- | --- | --- | --- | --- | --- | --- | --- | --- | --- | --- | --- | --- | --- | --- | --- | --- |
| Litres | 20 | 20 | 20 | 20 | 20 | 20 | 20 | 10 | 5 | 20 | 20 | 20 | 20 | 20 | 20 | 20 | 20 | 20 | 20 | 10 | 20 | 20 | 20 | 20 | 20 |
| Period | LH | LH | LH | MH | MH | MH | MH | MH | MH | MH | MH | MH | MH | MH | MH | EH | EH | EH | EH | EH | EH | EH | EH | EH | EH |
| Trianthema triquetra |  |  |  |  |  |  |  |  |  |  |  |  |  |  |  | 1 | 1 |  |  |  |  |  |  |  | 1 |
| Stellaria sp. | 9 |  |  | 1 |  | 1 |  |  |  |  | 1 |  | 2 |  |  | 2 |  | 82 | 2 |  | 6 |  |  |  |  |
| Eleocharis sp. | 2 |  |  |  |  |  |  |  |  |  |  | 2 |  |  | 1 | 1 | 14 |  | 4 |  | 2 |  |  | 1 | 1 |
| Scirpus sp. |  | 1 |  |  |  |  |  |  |  |  |  |  |  |  | 2 |  |  |  |  |  |  | 2 |  | 1 |  |
| Cyperaceae | 2 | 14 | 2 | 2 | 1 |  |  |  |  |  | 4 | 8 | 1 | 3 | 11 | 20 | 21 | 5 | 35 | 1 | 23 | 1 | 7 | 1 | 1 |
| (all) Fabaceae | 1 |  | 1 |  |  |  |  |  |  |  |  |  |  |  |  |  |  | 8 | 1 |  |  |  |  |  |  |
| Fumaria sp. |  |  |  |  |  |  |  |  |  |  |  |  |  |  |  |  | 1 |  | 2 |  |  |  |  | 1 |  |
| Papaveraceae |  | 1 |  |  |  |  |  |  |  |  |  |  |  |  |  |  |  |  |  |  |  |  |  |  |  |
| Chrysopogon sp. |  |  |  |  |  |  |  |  |  | 1 |  |  |  |  |  |  |  |  | 1 |  |  |  |  |  |  |
| Pennisetum sp. |  | 1 |  |  |  |  |  |  |  |  |  |  |  |  |  |  |  |  |  |  |  |  |  |  |  |
| Big Millet |  | 1 |  |  |  |  |  |  |  |  |  |  |  |  |  |  |  |  |  |  |  |  |  |  |  |
| Grass Type 1 |  |  |  |  |  |  |  |  |  |  |  |  |  |  |  |  |  |  | 2 |  |  |  |  |  |  |
| Indet. Grass | 1 | 1 |  |  |  |  |  |  |  |  | 2 |  | 1 |  | 2 | 2 |  | 5 | 2 | 2 | 1 | 1 | 2 | 4 |  |
| Polygonum sp. |  |  |  |  |  |  |  |  |  |  |  |  |  |  |  | 2 |  |  |  |  |  |  |  |  |  |
| Solanaceae |  |  |  |  |  |  |  |  |  |  |  |  |  |  |  |  |  |  | 2 |  |  |  |  |  |  |
| Indet. round weed | 2 | 16 | 2 | 1 |  |  | 1 |  |  |  | 1 | 1 | 2 | 6 | 3 | 1 | 3 | 3 | 5 |  |  | 1 | 4 | 1 |  |

**Table S7** Context-by-context raw counts of cereals at Masudpur I. Only MNI counts given (Bates 2016)

| Context | 109 | 110 | 111 | 113 | 115 | 116 | 119 | 120 | 121 | 125 | 126 | 128 | 302 | 303 | 304 | 305 | 308 | 310 | 314 | 317 | 319 | 323 | 321 | 129 | 130 | 132 | 134 | 135 | 137 |
| --- | --- | --- | --- | --- | --- | --- | --- | --- | --- | --- | --- | --- | --- | --- | --- | --- | --- | --- | --- | --- | --- | --- | --- | --- | --- | --- | --- | --- | --- |
| Litres | 40 | 40 | 60 | 40 | 5 | 2 | 40 | 40 | 20 | 40 | 40 | 40 | 40 | 40 | 40 | 80 | 40 | 80 | 40 | 40 | 40 | 40 | 40 | 40 | 40 | 40 | 20 | 40 | 10 |
| Hordeum vulgare |  |  | 1 | 1 | 11 | 5 |  | 12 |  | 81 | 5 | 16 |  |  |  |  |  | 5 | 4 | 10 | 14 | 154 | 133 |  | 3 |  | 69 | 6 | 57 |
| 6-row Hord. rachis |  |  |  |  | 4 |  |  |  |  | 1 |  |  |  |  |  |  |  | 1 | 3 |  |  | 6 |  |  |  |  | 1 |  |  |
| 2-row Hord. rachis |  |  |  |  | 2 |  |  |  |  | 6 |  |  |  |  |  |  |  |  |  |  |  | 2 |  |  |  |  | 1 |  |  |
| Hord. rachis |  |  |  |  | 3 |  |  |  |  | 73 |  |  |  |  |  |  |  |  |  |  |  |  |  |  |  |  | 6 |  |  |
| cf. Hord. rachis |  |  |  |  | 11 |  |  |  |  |  |  |  |  |  |  |  |  |  |  |  |  |  |  |  |  |  |  |  |  |
| Triticum sp. |  |  |  |  | 3 |  |  |  |  | 15 |  | 3 |  |  |  |  |  | 2 | 3 |  | 1 | 6 | 17 |  | 1 |  |  | 2 | 5 |
| Hordeum/Triticum |  | 2 |  | 1 | 1 | 2 | 1 | 20 | 2 | 24 | 4 | 9 |  |  | 5 |  | 15 |  | 4 | 1 | 1 | 98 | 42 | 1 |  | 1 | 66 |  | 1 |
| Large cereal rachis |  |  |  |  |  |  |  |  |  | 127 |  |  |  |  |  |  |  |  |  |  |  |  | 1 |  |  |  |  |  |  |
| Oryza sp. | 1 |  |  | 2 | 1 |  | 1 |  |  | 3 |  | 1 |  |  |  | 1 | 1 | 2 | 3 | 18 | 22 | 1008 | 12 | 2 |  |  |  |  | 2 |
| Domest. Oryza spikelet base |  |  |  |  |  |  |  |  |  |  |  |  |  |  |  |  |  | 3 | 3 | 2 | 47 | 48 | 1 |  |  |  |  |  |  |
| Wild Oryza spikelet base |  |  |  |  |  |  |  |  |  |  |  |  |  |  |  |  |  | 2 |  | 4 | 59 | 472 | 8 |  |  |  |  |  |  |
| Immature Oryza spikelet base |  |  |  |  |  |  |  |  | 1 |  |  |  |  |  |  |  |  |  | 1 |  | 9 | 50 |  |  |  |  |  |  |  |
| Indet. Oryza spikelet base | 1 |  |  |  |  |  |  |  |  |  |  |  |  |  |  |  |  | 4 | 8 |  | 78 | 606 | 5 |  |  |  |  |  |  |
| Silicified Oryza spikelet base |  |  |  |  |  |  |  |  |  |  |  |  |  |  |  |  |  | 3 |  |  | 2 |  | 1 |  |  |  |  |  |  |
| Oryza husk fragment |  |  |  |  |  |  |  |  |  |  |  |  |  |  |  |  |  | 1 |  |  |  |  |  |  |  |  |  |  |  |
| Echinochloa sp. | 1 | 14 | 13 |  | 2 |  | 7 | 24 |  | 17 | 2 | 3 | 1 |  | 1 | 6 | 10 | 22 | 24 | 44 | 61 | 266 | 37 | 6 | 1 | 2 | 41 | 2 | 2 |
| Setaria sp. |  |  | 2 | 1 | 1 |  | 5 | 16 |  | 7 | 2 | 4 |  | 2 | 2 | 2 |  | 5 | 27 | 66 | 27 | 706 | 24 | 10 | 1 |  | 28 | 1 | 1 |
| Panicum sp. | 1 | 2 | 1 |  | 17 |  |  | 4 |  | 6 | 2 |  |  |  |  | 1 |  | 2 | 9 | 14 | 1 | 12 | 5 |  | 1 |  | 1 |  |  |
| SEB |  | 4 | 2 | 1 | 1 |  |  | 12 |  | 6 |  | 3 |  |  |  | 3 | 9 | 19 | 6 | 4 | 2 | 86 | 5 | 16 | 2 |  | 13 | 2 |  |
| Indet. millet |  |  |  | 2 | 3 |  | 7 | 12 |  | 21 | 1 |  | 1 |  | 2 | 1 | 2 | 6 | 16 | 22 | 11 | 228 | 12 |  |  |  | 25 |  | 4 |

**Table S8** Context-by-context raw counts of weeds at Masudpur I. Only MNI counts given (Bates 2016)

| Context | 109 | 110 | 111 | 113 | 115 | 116 | 119 | 120 | 121 | 125 | 126 | 128 | 302 | 303 | 304 | 305 | 308 | 310 | 314 | 317 | 319 | 323 | 321 | 129 | 130 | 132 | 134 | 135 | 137 |
| --- | --- | --- | --- | --- | --- | --- | --- | --- | --- | --- | --- | --- | --- | --- | --- | --- | --- | --- | --- | --- | --- | --- | --- | --- | --- | --- | --- | --- | --- |
| Litres | 40 | 40 | 60 | 40 | 5 | 2 | 40 | 40 | 20 | 40 | 40 | 40 | 40 | 40 | 40 | 80 | 40 | 80 | 40 | 40 | 40 | 40 | 40 | 40 | 40 | 40 | 20 | 40 | 10 |
| Trianthema triquetra |  |  |  |  |  |  |  |  |  |  |  |  |  |  |  |  |  |  |  |  |  | 12 | 3 |  |  |  | 3 |  |  |
| Stellaria sp. |  |  |  |  |  |  |  |  |  | 2 |  |  |  |  |  |  | 1 |  |  |  |  |  | 2 |  |  |  |  |  |  |
| Stellaria nemorum |  |  |  |  |  |  |  |  |  |  |  |  |  |  |  |  |  |  |  |  |  | 2 |  |  |  |  |  |  |  |
| Chenopodium album |  |  |  |  |  |  |  |  |  | 6 |  |  |  |  |  |  |  |  |  |  |  |  |  |  |  |  |  |  |  |
| Eleocharis sp. |  |  |  |  |  |  |  |  |  |  | 2 | 2 |  |  |  | 1 | 2 | 1 | 6 | 8 | 1 | 2 | 12 |  | 1 |  |  | 2 |  |
| Scirpus sp. |  |  |  |  |  |  |  |  |  | 2 |  |  |  |  |  |  |  |  |  | 16 |  |  |  |  |  |  |  |  |  |
| Cyperaceae | 1 | 8 | 46 | 8 | 11 |  | 2 |  | 2 | 105 | 7 | 15 | 1 |  | 3 | 6 | 10 | 31 | 21 | 40 | 54 | 68 | 21 | 22 | 4 |  | 1 | 4 | 1 |
| Medicago/Melilotus/  Trifolium | 1 |  |  | 7 |  |  |  |  |  | 8 | 3 | 3 |  |  |  |  |  | 5 |  | 64 | 3 | 14 | 15 | 4 |  |  | 6 | 6 |  |
| (all) Fabaceae | 1 | 2 | 1 |  |  |  | 5 | 4 |  | 9 |  |  |  |  | 1 | 1 | 9 | 3 |  | 4 | 1 | 6 | 4 | 2 | 1 | 1 | 8 | 1 |  |
| Fumaria sp. |  |  |  |  |  |  |  |  |  | 1 |  |  | 2 |  |  |  | 1 |  |  |  | 1 | 6 | 6 |  |  |  | 1 |  |  |
| Aeluropus sp. |  |  |  |  |  |  |  |  |  |  |  |  |  |  |  |  |  |  |  |  |  | 16 |  |  |  |  |  |  |  |
| Avena sp. |  |  |  |  |  |  |  |  |  |  |  |  |  |  |  |  |  |  |  |  |  | 4 | 4 |  |  |  |  |  |  |
| Brachiaria sp. |  |  | 1 |  |  |  |  |  |  | 2 |  |  |  |  |  |  |  | 1 |  |  |  |  |  |  | 1 |  | 1 |  |  |
| Chrysopogon sp. |  |  |  |  |  |  |  |  |  | 4 |  |  |  |  |  |  |  |  |  |  |  | 66 | 7 |  |  |  | 4 |  |  |
| Coix sp. |  |  |  |  |  |  |  |  |  |  |  |  |  |  |  |  |  |  |  |  |  | 2 |  |  |  |  |  |  |  |
| Echinochloa crus-galli |  |  |  |  |  |  | 1 |  |  |  |  |  |  |  |  |  |  | 1 |  | 2 | 1 |  |  |  |  |  | 1 |  |  |
| Eragrostis sp. |  |  |  |  |  |  |  |  |  | 899 |  |  |  |  |  |  |  | 1 |  |  |  |  | 7 |  |  |  | 1 |  |  |
| Big Millet |  |  |  |  |  |  |  |  |  |  |  |  |  |  |  |  |  |  |  |  |  | 4 |  |  |  |  |  |  |  |
| Large Grass |  |  |  |  |  |  |  | 8 |  | 72 |  |  |  |  |  |  |  |  |  |  |  | 54 | 15 |  |  |  | 12 |  |  |
| Grass type 1 |  | 8 |  |  |  |  |  | 4 |  | 10 |  |  |  |  |  |  |  |  |  |  | 3 | 10 | 7 |  |  |  | 4 |  |  |
| Indet. Grass | 2 |  |  | 4 | 11 |  | 2 | 4 | 4 | 22 | 3 | 6 |  |  |  | 5 | 4 | 12 | 11 | 66 | 4 | 12 | 9 | 12 | 4 |  | 17 | 7 | 5 |
| Polygonum sp. |  | 4 |  |  |  |  |  |  |  | 1 |  |  |  |  | 1 |  |  |  |  |  |  |  |  |  |  |  | 1 |  |  |
| Solanaceae |  |  |  |  |  |  |  |  |  |  |  |  |  |  |  |  |  |  |  |  |  |  | 1 |  |  |  | 3 |  |  |
| Curled embryo |  |  |  |  |  |  |  | 4 |  | 18 |  |  |  |  |  |  |  |  |  |  |  | 2 |  |  |  |  | 6 |  |  |
| Indet. round weed |  | 4 |  | 14 |  | 2 | 5 | 36 | 1 | 14 | 1 | 6 | 4 |  | 3 | 11 | 16 | 122 |  | 122 | 70 | 13704 | 120 | 12 | 1 |  | 13 | 5 |  |

**Table S9** Context-by-context raw counts of cereals at Bahola. Only MNI counts given (Bates 2016)

| Context | 109 | 110 | 111 | 112 | 113 | 115 | 116 | 120 | 121 | 133 | 117 | 122 | 123 | 125 | 126 | 127 | 128 | 129 | 131 | 131g | 132 | 134 | 135 | 136 | 137 | 138 | 139 | 140 | 141 | 143 |
| --- | --- | --- | --- | --- | --- | --- | --- | --- | --- | --- | --- | --- | --- | --- | --- | --- | --- | --- | --- | --- | --- | --- | --- | --- | --- | --- | --- | --- | --- | --- |
| Litres | 30 | 30 | 20 | 20 | 33 | 40 | 30 | 30 | 30 | 30 | 30 | 29 | 30 | 30 | 30 | 15 | 9 | 25 | 15 | 1 | 26 | 15 | 30 | 30 | 35 | 30 | 30 | 30 | 30 | 30 |
| Period | PGW | PGW | PGW | PGW | PGW | PGW | PGW | PGW | PGW | PGW | LH | LH | LH | LH | LH | LH | LH | LH | LH | LH | LH | LH | LH | LH | LH | LH | LH | LH | LH | LH |
| Hordeum vulg. | 2 |  |  |  | 1 |  |  |  |  |  |  |  |  | 1 |  |  |  |  | 1 |  |  |  |  |  | 1 |  |  |  | 2 |  |
| 6-row Hord. rachis |  |  |  |  |  |  |  |  |  |  |  |  |  |  |  |  |  |  |  | 2 |  |  |  | 1 |  |  |  |  |  |  |
| Triticum sp. |  |  |  | 1 |  |  |  |  |  |  |  |  |  |  |  |  |  |  |  |  |  |  |  |  |  |  |  |  |  |  |
| Hordeum/Triticum | 1 | 2 |  |  | 6 |  | 1 |  | 3 |  | 2 | 2 |  | 2 |  | 2 |  |  | 2 | 1 |  | 1 |  | 2 | 2 | 1 |  |  | 4 | 1 |
| Oryza sp. |  |  |  | 1 | 6 | 1 |  | 1 | 1 | 1 |  | 1 |  | 1 |  |  |  |  | 10 | 4 |  | 2 | 1 | 1 | 1 | 1 |  | 2 | 5 | 1 |
| Domest. Oryza spikelet base | 1 |  |  |  |  |  |  |  |  |  |  |  |  | 2 | 1 |  |  |  | 2 |  |  |  |  |  | 3 |  |  |  | 2 |  |
| Wild Oryza spikelet base |  |  |  |  |  |  |  |  |  |  |  |  |  |  | 1 |  |  |  |  |  |  |  |  |  |  |  |  |  |  |  |
| Indet. Oryza spikelet base | 2 | 1 |  |  | 7 |  |  |  |  |  |  |  |  | 2 | 5 |  | 1 |  | 1 |  |  |  |  |  | 3 |  |  |  |  | 1 |
| Silicified Oryza spikelet base |  |  |  |  |  |  |  |  |  |  |  |  |  |  |  |  |  |  |  |  |  |  |  |  | 1 |  |  |  |  |  |
| Echinochloa sp. | 2 |  | 1 |  | 4 | 4 | 2 | 5 |  | 2 | 2 | 8 |  | 1 | 1 | 1 |  | 2 | 5 | 6 | 4 |  |  | 1 | 1 |  | 5 | 1 | 12 | 4 |
| Setaria sp. |  |  |  |  | 3 | 3 | 1 | 2 | 1 | 2 | 2 | 4 |  | 3 |  |  |  |  | 7 | 3 | 1 |  |  | 1 |  | 1 | 3 |  | 12 | 4 |
| Panicum sp. |  |  | 1 |  |  |  | 2 |  |  | 2 |  | 1 |  |  |  |  |  |  |  |  |  |  |  |  | 1 |  | 1 |  | 2 | 1 |
| SEB |  |  |  |  | 4 | 2 |  |  |  |  |  |  |  | 1 | 1 | 1 |  |  | 2 |  | 1 |  |  | 4 |  | 2 |  |  | 2 |  |
| Indet. millet | 2 |  | 3 |  | 7 | 1 |  | 4 | 5 | 7 | 4 | 8 |  | 3 | 4 | 3 | 3 |  | 2 | 3 |  | 1 | 3 |  | 3 |  | 5 |  | 7 | 7 |

**Table S10** Context-by-context raw counts of weeds at Bahola. Only MNI counts given. (Bates 2016)

| Context | 109 | 110 | 111 | 112 | 113 | 115 | 116 | 120 | 121 | 133 | 117 | 122 | 123 | 125 | 126 | 127 | 128 | 129 | 131 | 131g | 132 | 134 | 135 | 136 | 137 | 138 | 139 | 140 | 141 | 143 |
| --- | --- | --- | --- | --- | --- | --- | --- | --- | --- | --- | --- | --- | --- | --- | --- | --- | --- | --- | --- | --- | --- | --- | --- | --- | --- | --- | --- | --- | --- | --- |
| Litres | 30 | 30 | 20 | 20 | 33 | 40 | 30 | 30 | 30 | 30 | 30 | 29 | 30 | 30 | 30 | 15 | 9 | 25 | 15 | 1 | 26 | 15 | 30 | 30 | 35 | 30 | 30 | 30 | 30 | 30 |
| Period | PGW | PGW | PGW | PGW | PGW | PGW | PGW | PGW | PGW | PGW | LH | LH | LH | LH | LH | LH | LH | LH | LH | LH | LH | LH | LH | LH | LH | LH | LH | LH | LH | LH |
| Trianthema triquetra |  |  |  |  |  |  |  |  |  |  |  |  |  |  |  |  |  |  |  |  |  |  | 1 |  |  |  |  |  | 1 |  |
| Stellaria sp. | 1 |  |  |  |  |  |  | 1 |  |  |  | 1 |  |  |  |  |  |  |  |  |  |  |  |  |  |  |  |  |  |  |
| Chenopodium sp. | 1 |  |  |  |  |  |  |  |  |  |  |  |  |  |  |  |  | 1 |  | 3 | 3 |  |  |  |  |  |  |  |  |  |
| Eleocharis sp. |  |  |  | 1 | 1 |  |  | 1 | 1 | 1 | 1 | 2 |  | 2 | 4 |  |  | 2 |  | 1 | 15 | 1 |  | 4 | 1 |  |  |  |  | 2 |
| Scirpus sp. |  |  |  |  | 1 |  |  |  |  |  |  |  |  |  |  |  |  |  |  |  |  |  |  |  |  |  |  |  |  |  |
| Cyperaceae | 1 | 2 |  |  | 15 | 8 | 17 | 2 | 8 | 3 | 6 | 11 | 6 | 17 | 3 | 1 | 1 | 3 | 22 | 17 | 3 |  | 6 | 10 | 7 | 2 | 9 | 4 | 13 | 7 |
| (all) Fabaceae |  |  |  |  | 4 |  |  |  | 1 |  |  | 2 |  | 1 |  |  |  | 1 |  | 1 |  | 1 |  |  |  |  |  |  |  |  |
| Chrysopogon sp. |  |  |  |  | 2 |  |  |  |  |  |  | 1 |  | 5 | 1 |  |  | 1 | 9 | 9 | 1 |  | 1 | 1 |  |  |  |  | 7 |  |
| Echinochloa crus-galli |  |  |  |  |  |  |  |  |  |  |  |  |  |  |  |  |  |  |  |  | 1 |  |  |  | 2 | 2 |  |  |  |  |
| Eragrostis sp. |  | 4 |  | 1 | 2 |  | 1 | 1 |  |  | 2 | 2 |  |  |  |  |  | 1 |  |  |  | 1 | 1 | 1 |  |  | 1 | 1 | 1 |  |
| Paspaspalum sp. |  |  |  |  |  | 1 |  |  |  |  |  |  |  |  |  |  |  |  |  |  |  |  |  |  |  |  |  |  |  |  |
| Grass type 1 |  |  | 1 |  | 4 | 2 |  |  | 1 | 1 |  | 1 |  |  |  |  |  |  |  |  |  |  |  |  |  |  |  |  | 1 |  |
| Indet. Grass |  | 1 | 1 |  | 7 | 1 | 4 | 1 | 5 | 3 | 6 | 4 | 2 | 3 |  | 1 | 3 |  | 1 | 2 | 2 |  | 2 | 7 | 2 | 11 |  | 4 | 5 | 7 |
| Rumex sp. |  |  |  |  |  |  |  |  |  |  |  |  |  |  |  |  |  |  |  |  |  |  |  |  |  |  |  |  | 1 |  |
| Polygonum sp. |  |  |  |  | 1 |  |  |  |  |  |  |  |  |  |  |  |  |  |  |  |  |  |  |  |  |  |  |  |  |  |
| Curled embryo |  |  |  |  |  |  |  |  |  |  |  |  |  |  |  |  |  |  |  |  |  |  |  |  |  |  |  |  | 1 |  |
| Indet. round weed | 1 |  |  |  |  |  |  |  | 1 |  |  |  |  |  |  | 1 | 1 | 3 | 11 |  | 1 |  |  |  |  | 3 |  | 1 | 5 | 5 |

**II Phytoliths**

**Table S11** Context-by-context raw counts of phytoliths at Dabli vas Chugta. Only those forms included in the analysis have been outlined here. For full dataset see Bates (2016)

| Context | 204 | 205 | 206 | 207 | 224 | 225 | 226 | 227 | 229 | 230 | 231 | 233 | 234 | 235 | 236 | 238 | 239 | 240 | 242 | 243 | 244 | 245 | 248 | 249 | 250 | 251 |
| --- | --- | --- | --- | --- | --- | --- | --- | --- | --- | --- | --- | --- | --- | --- | --- | --- | --- | --- | --- | --- | --- | --- | --- | --- | --- | --- |
| Elongate psilate | 30 | 30 | 33 | 26 | 28 | 43 | 50 | 42 | 47 | 47 | 43 | 41 | 46 | 35 | 66 | 48 | 43 | 46 | 47 | 52 | 64 | 79 | 62 | 61 | 61 | 65 |
| Elongate irregular |  |  | 2 | 1 |  | 3 |  |  | 4 | 1 | 1 | 1 |  |  |  | 1 |  |  |  |  |  |  | 2 | 2 |  | 1 |
| Elongate echinate Indet. | 4 | 8 | 6 | 9 | 6 | 6 | 12 | 5 | 14 | 5 | 10 | 13 | 8 | 11 | 18 | 3 | 3 | 11 | 6 | 9 | 5 | 21 | 16 | 13 | 21 | 12 |
| Echinate wheat/barley type | 7 |  | 5 | 11 | 4 | 4 | 12 | 24 | 7 | 2 | 7 | 4 | 2 | 7 | 18 | 3 | 5 | 6 | 3 | 7 | 6 | 33 | 12 | 9 | 8 | 4 |
| Elongate dendritic wheat/barley type |  | 1 |  | 2 | 1 |  |  |  | 3 |  |  | 1 |  |  |  |  |  |  |  |  |  | 5 |  |  |  |  |
| Elongate echinate short spine |  |  | 1 |  |  | 1 | 1 |  |  | 1 | 2 | 1 |  |  |  |  |  |  |  |  | 1 | 4 |  |  |  |  |
| Elongate echinate hook spine |  |  |  |  |  |  |  |  |  |  |  |  |  | 1 |  |  |  |  |  |  |  |  |  |  |  |  |
| Elongate dendritic short spine |  |  |  |  |  |  |  |  |  |  |  |  |  |  |  |  |  |  |  |  |  | 1 |  |  |  |  |
| Elongate echinate wavy millet Indet | 3 | 4 | 1 | 2 |  | 3 | 3 | 5 | 1 |  | 1 | 1 | 5 | 2 |  |  | 1 |  | 2 | 2 | 3 | 2 | 5 | 3 | 1 | 1 |
| Elongate echinate wavy Echinochloa |  |  |  |  |  |  |  |  |  |  |  |  |  |  |  |  |  |  |  |  |  |  |  |  |  |  |
| Elongate echinate wavy Brachiaria |  |  |  |  |  |  |  |  |  |  |  |  |  |  |  |  |  |  |  |  |  |  |  |  |  |  |
| Elongate echinate wavy Pennisetum |  |  |  |  |  |  |  |  |  |  |  |  |  |  |  |  |  |  |  |  |  |  |  |  |  |  |
| Elongate echinate wavy cf. Sorghum |  |  |  |  |  |  |  |  |  |  |  |  |  |  |  |  |  |  |  |  |  |  |  |  |  |  |
| Elongate echinate wavy cf. Setaria verticillata |  |  |  |  |  |  |  |  |  |  |  |  |  |  |  |  |  |  |  |  |  |  |  |  |  |  |
| Elongate echinate wavy cf. Setaria italica |  |  |  |  |  |  |  |  |  |  |  |  |  |  |  |  |  |  |  |  |  |  |  |  |  |  |
| Elongate echinate wavy Panicum miliaceum |  |  |  |  |  |  |  |  |  |  |  |  |  |  |  |  |  |  |  |  |  |  |  |  |  |  |
| Elongate non-psilate | 3 | 4 | 2 |  | 1 |  |  | 3 | 2 | 5 |  |  | 1 | 1 | 3 | 2 | 1 |  | 1 | 1 | 1 | 1 | 2 |  | 5 | 1 |
| Cross | 3 | 7 | 5 | 6 | 1 | 10 | 8 | 9 | 8 | 2 | 3 | 2 | 3 |  |  |  |  |  |  |  |  |  |  |  |  |  |
| Double peak husk |  |  |  |  |  |  |  |  |  |  |  |  |  |  |  |  |  |  |  |  |  |  |  |  |  |  |
| Commelinaceae (Eichhorn L) |  |  |  |  |  |  |  |  |  |  |  |  |  |  |  |  |  |  |  |  |  |  |  |  |  |  |
| Commelinaceae (Eichhorn K) |  |  |  |  |  |  |  |  |  |  |  |  |  |  |  |  |  |  |  |  |  |  |  |  |  |  |
| Commelinaceae (Eichhorn A) |  |  |  |  |  |  |  |  |  |  |  |  |  |  |  |  |  |  |  |  |  |  |  |  |  |  |
| Commelinaceae (Eichhorn I) |  |  |  |  |  |  |  |  |  |  |  |  |  |  |  |  |  |  |  |  |  |  |  |  |  |  |
| Commelinaceae Indet. Cone |  |  |  |  |  |  |  |  |  |  |  |  |  | 354 | 353 | 351 | 351 | 357 | 376 | 360 | 350 | 348 | 354 | 357 | 348 | 351 |
| Total (including other forms not listed here) | 340 | 348 | 351 | 366 | 344 | 346 | 396 | 359 | 380 | 406 | 355 | 366 | 351 |  |  |  |  |  |  |  |  |  |  |  |  |  |

**Table S11** Continued

| Context | 252 | 253 | 254 | 255A | 255B | 255C | 256 | 257 | 258 | 259 |
| --- | --- | --- | --- | --- | --- | --- | --- | --- | --- | --- |
| Elongate psilate | 63 | 46 | 65 | 65 | 76 | 48 | 61 | 82 | 63 | 62 |
| Elongate irregular | 2 |  |  |  |  | 1 | 1 |  | 3 |  |
| Elongate echinate Indet. | 11 | 7 | 16 | 18 | 11 | 20 | 24 | 5 | 13 | 13 |
| Echinate wheat/barley type | 8 | 10 | 7 | 31 | 16 | 20 | 19 | 17 | 16 | 22 |
| Elongate dendritic wheat/barley type | 2 |  | 1 |  |  |  |  |  | 1 |  |
| Elongate echinate short spine |  |  | 1 |  | 1 |  |  | 2 |  |  |
| Elongate echinate hook spine |  |  |  |  |  |  |  |  |  |  |
| Elongate dendritic short spine |  |  |  |  |  |  |  |  |  |  |
| Elongate echinate wavy millet Indet |  | 3 | 1 | 4 | 2 | 3 | 5 | 1 | 3 | 6 |
| Elongate echinate wavy Echinochloa |  |  |  |  |  |  |  |  |  |  |
| Elongate echinate wavy Brachiaria |  |  |  |  |  |  |  |  |  |  |
| Elongate echinate wavy Pennisetum |  |  |  |  |  |  |  |  |  |  |
| Elongate echinate wavy cf. Sorghum |  |  |  |  |  |  |  |  |  |  |
| Elongate echinate wavy cf. Setaria verticillata |  |  |  |  |  |  |  |  |  |  |
| Elongate echinate wavy cf. Setaria italica |  |  |  |  |  |  |  |  |  |  |
| Elongate echinate wavy Panicum miliaceum |  |  |  |  |  |  |  |  |  |  |
| Elongate non-psilate | 1 | 1 | 1 | 2 | 1 | 3 |  | 3 |  |  |
| Double peak husk |  |  |  |  |  |  |  |  |  |  |
| Commelinaceae (Eichhorn L) |  |  |  |  |  |  |  |  |  |  |
| Commelinaceae (Eichhorn K) |  |  |  |  |  |  |  |  |  |  |
| Commelinaceae (Eichhorn A) |  |  |  |  |  |  |  |  |  |  |
| Commelinaceae (Eichhorn I) |  |  |  |  |  |  |  |  |  |  |
| Commelinaceae Indet. Cone |  |  |  |  |  |  |  |  |  |  |
| Total (including other forms not listed here) | 358 | 339 | 361 | 355 | 344 | 373 | 358 | 353 | 347 | 345 |

**Table S12** Context-by-context raw counts of phytoliths at Burj. Only those forms included in the analysis have been outlined here. For full dataset see Bates (2016)

| Context | 140 | 141 | 142 | 145 | 150 | 151 | 152 | 157 | 158 | 162 | 209 | 210 | 212 | 213 | 215 | 218 | 219 | 220 | 224 |
| --- | --- | --- | --- | --- | --- | --- | --- | --- | --- | --- | --- | --- | --- | --- | --- | --- | --- | --- | --- |
| Period | PGW | PGW | PGW | PGW | PGW | PGW | PGW | PGW | PGW | PGW | EH | EH | EH | EH | EH | EH | EH | EH | EH |
| Elongate psilate | 26 | 26 | 34 | 33 | 26 | 32 | 27 | 38 | 61 | 41 | 37 | 29 | 32 | 30 | 34 | 23 | 37 | 11 | 41 |
| Elongate irregular |  | 5 |  | 1 |  |  |  | 1 |  |  |  |  | 6 |  |  |  | 1 | 2 | 2 |
| Elongate echinate Indet. | 2 | 5 | 7 | 9 | 3 |  | 2 | 2 | 1 | 4 | 9 | 12 | 12 | 3 | 3 | 8 | 8 | 5 | 8 |
| Echinate wheat/barley type | 1 | 3 | 5 | 3 | 1 | 1 | 3 | 3 | 2 | 8 | 10 | 5 | 1 | 5 | 14 | 7 | 4 | 1 | 7 |
| Elongate dendritic wheat/barley type | 1 | 1 |  |  | 1 | 2 |  |  |  |  | 3 | 1 |  | 1 |  | 4 | 2 |  |  |
| Elongate echinate short spine | 2 |  | 2 |  | 1 | 2 | 2 |  | 1 |  |  |  | 3 | 1 |  |  | 3 |  | 2 |
| Elongate echinate hook spine |  |  |  |  |  |  |  |  |  |  |  |  |  |  |  |  |  |  | 1 |
| Elongate dendritic short spine |  |  |  |  |  |  |  |  |  |  |  |  |  |  |  |  |  |  |  |
| Elongate echinate wavy millet Indet | 2 |  |  |  | 5 | 3 | 3 | 2 | 7 | 4 | 2 | 9 | 1 |  | 1 | 1 |  |  |  |
| Elongate echinate wavy Echinochloa |  |  |  |  |  |  |  |  |  |  |  |  |  |  |  |  |  |  |  |
| Elongate echinate wavy Brachiaria |  |  |  |  |  |  |  |  |  |  |  |  |  |  |  |  |  |  |  |
| Elongate echinate wavy Pennisetum |  |  |  |  |  |  |  |  | 1 |  |  |  |  |  |  |  |  |  |  |
| Elongate echinate wavy cf. Sorghum |  |  |  |  |  |  |  |  |  |  |  |  |  |  |  |  |  |  |  |
| Elongate echinate wavy cf. Setaria verticillata |  |  |  |  |  |  |  |  |  |  |  |  |  |  |  | 1 |  |  |  |
| Elongate echinate wavy cf. Setaria italica |  |  |  |  |  |  |  |  |  |  |  |  |  |  |  | 1 |  |  |  |
| Elongate echinate wavy Panicum miliaceum |  |  |  |  |  |  |  |  |  |  |  |  |  |  |  |  |  |  |  |
| Elongate non-psilate |  | 4 | 2 | 1 | 5 | 2 | 1 |  | 2 | 1 | 2 | 4 | 1 | 3 | 2 | 5 | 4 |  | 3 |
| Double peak husk |  |  |  |  |  |  |  |  |  |  |  |  |  |  |  |  |  |  |  |
| Commelinaceae (Eichhorn L) |  |  |  |  |  |  |  |  |  |  |  |  |  |  |  |  |  |  |  |
| Commelinaceae (Eichhorn K) |  |  |  |  |  |  |  |  |  |  |  |  |  |  |  |  |  |  |  |
| Commelinaceae (Eichhorn A) |  |  |  |  |  |  |  |  |  |  |  |  | 2 |  |  |  |  |  |  |
| Commelinaceae (Eichhorn I) |  |  |  |  |  |  |  |  |  |  |  |  |  |  |  |  |  |  |  |
| Commelinaceae Indet. Cone |  |  |  |  |  |  |  |  |  |  |  |  |  |  |  |  |  |  |  |
| Total (including other forms not listed here) | 359 | 381 | 355 | 368 | 364 | 356 | 360 | 351 | 364 | 380 | 407 | 361 | 366 | 384 | 362 | 363 | 357 | 343 | 355 |

**Table S13** Context-by-context raw counts of phytoliths at Masudpur VII. Only those forms included in the analysis have been outlined here. For full dataset see Bates (2016)

| Context | 405 | 409 | 414 | 415 | 418 | 423 | 425 | 429 | 508 | 513 | 514 | 515 | 517 | 520 | 522 | 524 | 525 | 526 | 527 |
| --- | --- | --- | --- | --- | --- | --- | --- | --- | --- | --- | --- | --- | --- | --- | --- | --- | --- | --- | --- |
| Period | LH | MH | MH | MH | MH | EH | EH | EH | LH | MH | MH | LH | LH | MH | EH | EH | EH | EH | EH |
| Elongate psilate | 38 | 50 | 43 | 31 | 19 | 49 | 26 | 58 | 39 | 58 | 45 | 63 | 30 | 30 | 35 | 33 | 36 | 28 | 42 |
| Elongate irregular |  |  | 2 | 3 |  |  | 1 |  | 2 | 1 | 3 | 1 | 3 |  | 2 | 1 | 4 |  |  |
| Elongate echinate Indet. | 3 | 13 | 4 |  | 3 | 4 | 4 | 14 | 7 | 5 | 13 | 20 | 8 | 1 | 2 | 5 | 3 | 4 | 7 |
| Echinate wheat/barley type | 26 | 4 | 6 | 1 | 2 | 4 | 3 | 9 | 5 | 10 | 11 | 7 | 10 | 6 | 1 | 9 | 14 | 5 | 25 |
| Elongate dendritic wheat/barley type | 1 |  | 1 |  | 2 | 2 | 3 | 3 | 2 |  |  |  |  |  |  | 2 |  | 1 | 1 |
| Elongate echinate short spine |  |  |  |  | 2 |  |  | 4 |  |  |  | 1 | 1 |  |  |  |  | 1 |  |
| Elongate echinate hook spine |  |  |  |  |  |  |  |  |  | 1 |  |  |  | 1 |  |  |  |  |  |
| Elongate dendritic short spine |  |  |  |  |  |  |  |  |  |  |  |  |  |  |  |  |  |  |  |
| Elongate echinate wavy millet Indet |  | 3 | 1 |  | 1 | 1 | 3 | 3 |  | 2 | 1 |  | 3 | 2 | 1 | 2 | 1 | 1 |  |
| Elongate echinate wavy Echinochloa |  |  |  |  |  |  |  |  |  | 1 |  |  |  |  |  | 1 |  |  |  |
| Elongate echinate wavy Brachiaria |  |  |  |  |  |  |  |  |  |  |  |  |  |  |  |  |  |  |  |
| Elongate echinate wavy Pennisetum |  |  |  |  |  | 1 |  |  |  |  |  |  |  |  |  |  |  |  |  |
| Elongate echinate wavy cf. Sorghum |  |  |  |  |  |  |  |  |  |  |  |  |  |  |  |  |  |  |  |
| Elongate echinate wavy cf. Setaria verticillata |  |  |  |  |  |  |  |  |  |  |  |  |  |  |  |  |  |  |  |
| Elongate echinate wavy cf. Setaria italica |  |  |  |  |  |  |  |  |  |  |  |  |  |  |  |  |  |  |  |
| Elongate echinate wavy Panicum miliaceum |  |  |  |  |  |  |  |  |  |  |  |  |  |  |  |  |  |  |  |
| Elongate non-psilate | 5 | 1 | 3 | 2 | 5 | 4 | 2 | 2 |  | 4 | 1 | 3 |  | 4 | 3 | 3 | 2 | 1 | 3 |
| Double peak husk |  |  |  |  |  |  |  |  |  |  |  |  |  |  |  |  |  |  |  |
| Commelinaceae (Eichhorn L) |  |  |  |  |  |  |  |  |  |  |  |  |  |  |  |  |  |  |  |
| Commelinaceae (Eichhorn K) |  |  |  |  |  |  |  |  |  |  |  |  |  |  |  |  |  |  |  |
| Commelinaceae (Eichhorn A) |  |  |  |  |  |  |  |  |  |  |  |  |  |  |  |  |  |  |  |
| Commelinaceae (Eichhorn I) |  |  |  |  |  |  |  |  |  |  |  |  |  |  |  |  |  |  |  |
| Commelinaceae Indet. Cone |  |  |  |  |  |  |  |  |  |  |  |  |  | 1 |  | 1 |  | 1 |  |
| Total (including other forms not listed here) | 395 | 349 | 343 | 359 | 388 | 367 | 344 | 345 | 363 | 368 | 378 | 387 | 341 | 368 | 365 | 370 | 345 | 411 | 346 |

**Table S14** Context-by-context raw counts of phytoliths at Masudpur I. Only those forms included in the analysis have been outlined here. For full dataset see Bates (2016)

| Context | 111 | 115 | 125 | 128 | 129 | 130 | 132 | 134 | 137 | 302 | 303 | 304 | 305 | 310 | 314 | 315 | 316 | 317 | 319 | 323 |
| --- | --- | --- | --- | --- | --- | --- | --- | --- | --- | --- | --- | --- | --- | --- | --- | --- | --- | --- | --- | --- |
| Elongate psilate | 29 | 38 | 50 | 34 | 40 | 100 | 34 | 54 | 29 | 48 | 27 | 25 | 27 | 22 | 87 | 103 | 20 | 24 | 44 | 47 |
| Elongate irregular | 1 | 3 | 4 | 2 | 3 |  | 1 | 3 |  |  | 1 |  |  |  | 1 | 2 | 2 | 1 | 5 | 1 |
| Elongate echinate Indet. | 3 | 20 | 10 | 7 | 18 | 2 | 8 | 5 | 4 | 5 | 1 | 3 | 7 | 5 | 39 | 32 | 3 | 4 | 9 | 6 |
| Echinate wheat/barley type | 4 | 29 | 33 | 9 | 26 | 7 | 16 | 8 | 7 | 7 | 1 | 4 | 4 | 1 | 40 | 41 |  | 5 | 17 | 2 |
| Elongate dendritic wheat/barley type | 1 | 8 | 17 | 1 | 5 | 2 | 1 |  |  |  | 1 |  | 1 | 2 |  | 5 | 4 | 2 | 1 | 1 |
| Elongate echinate short spine | 3 | 11 | 7 |  | 3 | 1 |  | 5 | 2 | 4 |  | 3 |  |  |  | 7 | 1 | 3 | 2 |  |
| Elongate echinate hook spine |  |  |  |  |  |  |  |  |  | 1 |  |  |  |  |  |  | 1 |  |  |  |
| Elongate dendritic short spine |  | 1 | 1 |  |  |  |  |  |  |  |  |  |  |  |  |  |  |  | 1 |  |
| Elongate echinate wavy millet Indet |  |  | 2 |  |  |  |  | 1 | 3 | 3 |  |  | 6 | 2 | 6 | 5 | 1 | 1 | 1 |  |
| Elongate echinate wavy Echinochloa |  |  |  |  |  |  |  |  |  |  |  |  |  |  |  |  |  |  |  |  |
| Elongate echinate wavy Brachiaria |  |  |  |  |  | 1 |  |  |  |  |  |  |  |  |  |  |  |  |  |  |
| Elongate echinate wavy Pennisetum |  |  |  |  |  | 2 |  |  |  |  |  |  |  |  |  |  |  |  |  |  |
| Elongate echinate wavy cf. Sorghum |  |  |  |  |  |  |  |  |  |  |  |  |  |  |  |  |  |  |  | 1 |
| Elongate echinate wavy cf. Setaria verticillata |  |  |  |  |  |  |  |  |  |  |  |  |  |  |  |  |  |  |  |  |
| Elongate echinate wavy cf. Setaria italica |  |  |  |  |  |  |  |  |  |  |  |  |  |  |  |  |  |  |  |  |
| Elongate echinate wavy Panicum miliaceum |  |  |  |  |  |  |  |  |  |  |  |  |  |  |  |  |  |  |  |  |
| Elongate non-psilate | 4 | 1 | 2 |  | 7 | 5 | 5 | 5 | 3 | 1 | 1 | 3 | 4 | 6 | 6 | 1 | 4 | 2 | 5 | 5 |
| Double peak husk |  |  |  |  |  |  |  |  |  |  |  |  |  |  |  |  |  |  |  |  |
| Commelinaceae (Eichhorn L) |  |  |  |  |  |  |  |  | 1 |  |  |  |  |  |  |  |  |  |  | 2 |
| Commelinaceae (Eichhorn K) | 2 |  |  |  |  |  |  |  |  |  |  |  |  |  |  |  |  |  |  |  |
| Commelinaceae (Eichhorn A) | 1 |  |  |  |  |  |  |  |  |  |  |  |  |  |  |  |  |  |  |  |
| Commelinaceae (Eichhorn I) | 1 |  |  |  |  |  |  |  |  |  |  |  |  |  |  |  |  |  |  |  |
| Commelinaceae Indet. Cone |  |  |  |  |  |  |  |  |  | 2 |  |  |  |  | 1 |  |  |  |  |  |
| Total (including other forms not listed here) | 345 | 369 | 363 | 369 | 357 | 406 | 358 | 406 | 358 | 343 | 350 | 339 | 390 | 336 | 367 | 355 | 336 | 339 | 367 | 346 |

**Table S15** Context-by-context raw counts of phytoliths at Bahola. Only those forms included in the analysis have been outlined here. For full dataset see Bates (2016)

| Context | 110 | 110B | 112 | 113 | 115 | 116 | 117 | 118 | 120 | 121 | 123 | 124 | 125 | 125B | 127 | 128 | 129 | 131 | 132 | 133 | 134 | 134B | 135 | 136 | 137 | 138 | 140 | 141 | 143 | 145L |
| --- | --- | --- | --- | --- | --- | --- | --- | --- | --- | --- | --- | --- | --- | --- | --- | --- | --- | --- | --- | --- | --- | --- | --- | --- | --- | --- | --- | --- | --- | --- |
| Period | PGW | PGW | PGW | PGW | PGW | PGW | LH | LH | PGW | PGW | LH | LH | LH | LH | LH | LH | LH | LH | LH | PGW | LH | LH | LH | LH | LH | LH | LH | LH | LH | LH |
| Elongate psilate | 51 | 62 | 39 | 54 | 67 | 43 | 35 | 24 | 40 | 32 | 58 | 35 | 29 | 50 | 44 | 29 | 50 | 63 | 59 | 47 | 35 | 45 | 59 | 66 | 68 | 44 | 57 | 57 | 40 | 43 |
| Elongate irregular | 3 | 2 | 2 | 3 |  | 2 | 1 |  |  | 6 |  | 1 | 1 | 2 | 3 | 4 | 1 | 3 |  | 1 |  |  |  | 1 | 6 | 1 | 3 | 1 |  |  |
| Elongate echinate Indet. | 4 | 4 | 2 | 1 | 2 | 6 | 5 | 5 | 8 | 4 | 2 | 3 | 1 | 3 | 5 | 2 | 5 | 7 | 5 | 2 | 5 | 8 | 3 | 6 | 5 | 1 | 1 | 5 | 7 | 6 |
| Echinate wheat/barley type | 1 |  | 1 | 1 | 1 | 1 |  |  | 2 |  |  | 3 | 4 | 2 | 7 | 1 | 9 | 3 |  | 3 | 1 |  | 2 | 4 | 12 | 5 | 3 | 14 |  | 2 |
| Elongate dendritic wheat/barley type |  |  |  |  |  | 1 |  |  |  |  |  |  |  | 1 | 1 | 1 |  |  |  |  |  |  |  | 1 |  |  |  |  |  |  |
| Elongate echinate short spine |  | 1 |  | 3 |  |  |  |  |  |  | 2 |  |  |  |  | 1 |  |  |  |  |  |  |  | 1 | 3 |  | 1 |  |  | 1 |
| Elongate echinate hook spine |  |  |  | 1 |  |  |  |  |  |  |  |  |  |  |  |  |  | 1 |  |  |  |  |  |  |  |  |  |  |  |  |
| Elongate dendritic short spine |  |  |  |  | 1 |  |  |  |  |  |  |  |  |  |  |  |  |  |  |  |  |  |  |  |  |  |  |  |  |  |
| Elongate echinate wavy millet Indet | 5 | 5 | 2 | 2 | 2 |  | 6 | 1 | 4 | 5 |  | 3 | 2 |  | 2 | 2 | 1 |  | 3 |  | 2 | 1 | 4 | 1 | 3 | 5 | 3 | 2 | 6 |  |
| Elongate echinate wavy Echinochloa |  |  |  |  |  |  |  |  |  |  |  |  |  |  |  |  |  |  |  |  |  |  |  |  |  |  |  |  |  |  |
| Elongate echinate wavy Brachiaria |  |  |  |  |  |  |  |  |  |  |  |  |  |  |  |  |  |  |  |  |  |  |  |  |  |  |  |  |  |  |
| Elongate echinate wavy Pennisetum |  |  |  |  |  |  |  |  |  |  |  |  |  |  |  |  |  |  |  |  |  |  |  |  |  |  | 1 |  |  |  |
| Elongate echinate wavy cf. Sorghum |  |  |  |  |  |  |  |  |  |  |  |  |  |  |  |  |  |  |  |  |  |  |  |  |  |  |  |  |  |  |
| Elongate echinate wavy cf. Setaria verticillata |  |  |  |  |  |  |  |  |  |  |  |  |  |  |  |  |  |  |  |  |  |  |  |  |  |  |  |  |  |  |
| Elongate echinate wavy cf. S. italica |  |  |  |  |  |  |  |  |  |  |  |  |  |  |  |  |  |  |  |  |  |  |  |  |  |  |  |  |  |  |
| Elongate echinate wavy Panicum miliaceum |  |  |  |  |  |  |  |  | 1 |  |  |  |  |  |  |  |  |  |  |  |  |  |  |  |  |  |  |  |  |  |
| Elongate non-psilate |  |  |  |  | 1 |  |  |  |  |  |  |  |  |  | 1 |  |  |  |  |  |  |  | 2 | 2 |  |  |  |  |  |  |
| Double peak husk |  |  |  |  |  |  |  |  |  |  |  |  |  |  |  |  |  |  |  |  |  |  |  |  |  |  |  |  |  |  |
| Commelinaceae (Eichhorn L) |  |  |  |  |  |  |  |  |  |  |  |  |  |  |  |  |  |  |  |  |  |  |  |  |  |  |  |  |  |  |
| Commelinaceae (Eichhorn K) |  |  |  |  |  |  |  |  |  |  |  |  |  |  |  |  |  |  |  |  |  |  |  |  |  |  |  |  |  |  |
| Commelinaceae (Eichhorn A) |  |  |  |  |  |  |  |  |  |  |  |  |  |  |  |  |  |  |  |  |  |  |  |  |  |  |  |  |  |  |
| Commelinaceae (Eichhorn I) |  |  |  |  |  |  |  |  |  |  |  |  |  |  |  |  |  |  |  |  |  |  |  |  |  |  |  |  |  |  |
| Commelinaceae Indet. Cone |  |  |  |  |  |  |  |  |  |  |  |  |  |  |  |  |  |  |  |  |  |  |  |  |  |  |  |  |  |  |
| Total (incl. other forms not listed here) | 352 | 340 | 342 | 372 | 344 | 327 | 345 | 333 | 346 | 367 | 331 | 355 | 347 | 376 | 340 | 333 | 354 | 355 | 359 | 342 | 334 | 341 | 341 | 369 | 379 | 374 | 351 | 350 | 355 | 343 |

**III Phytolith images**

| 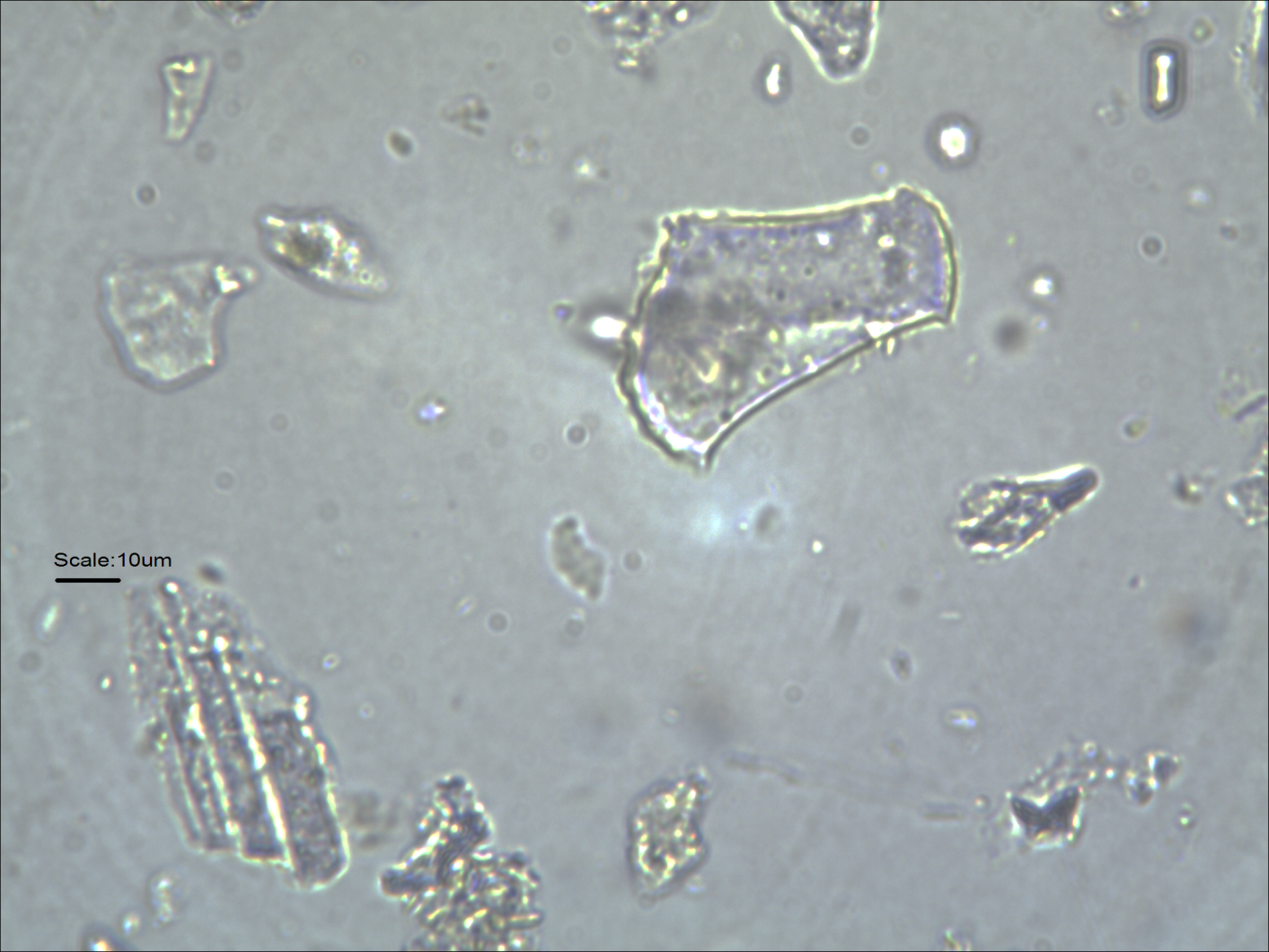 | 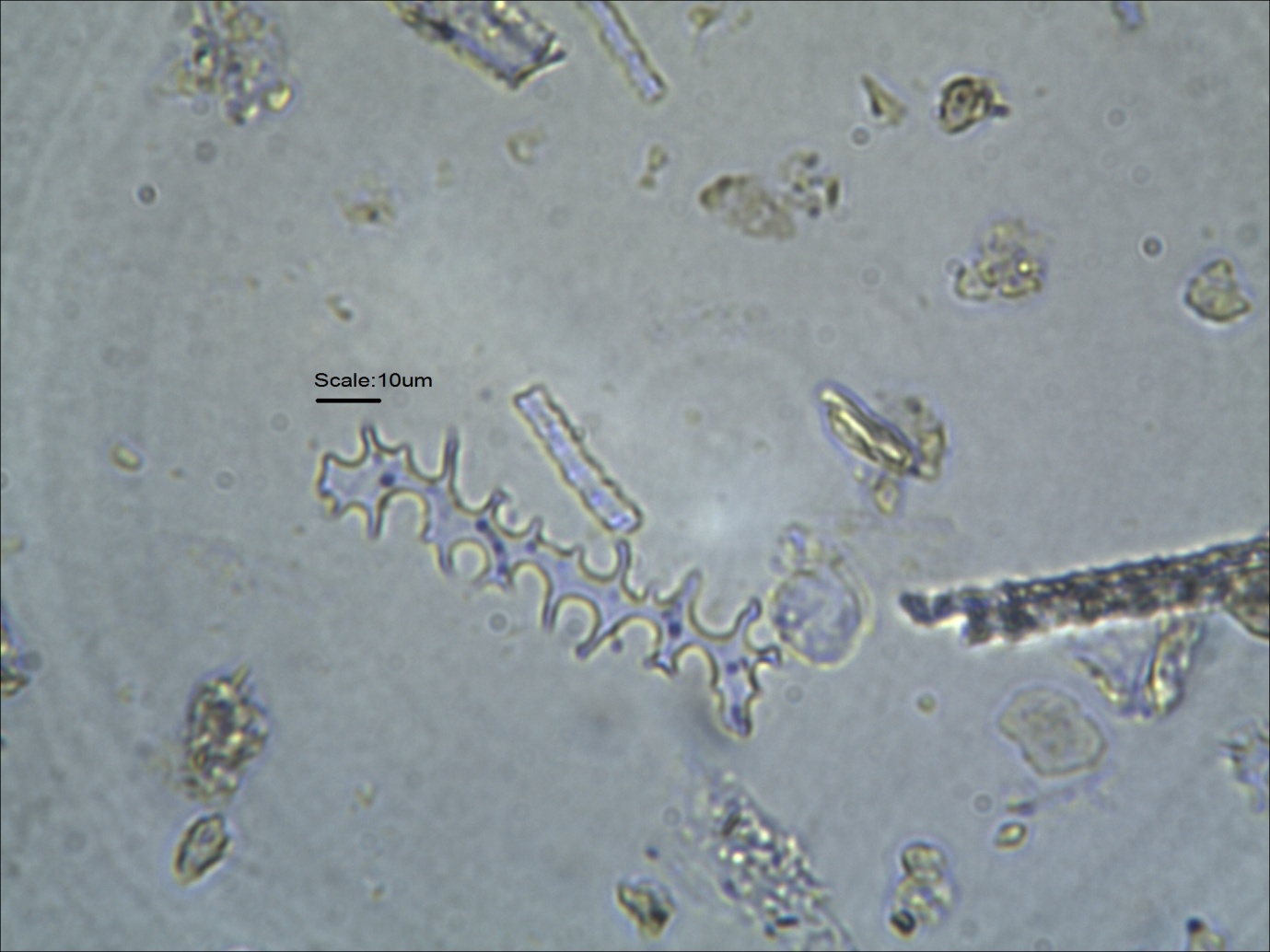 | 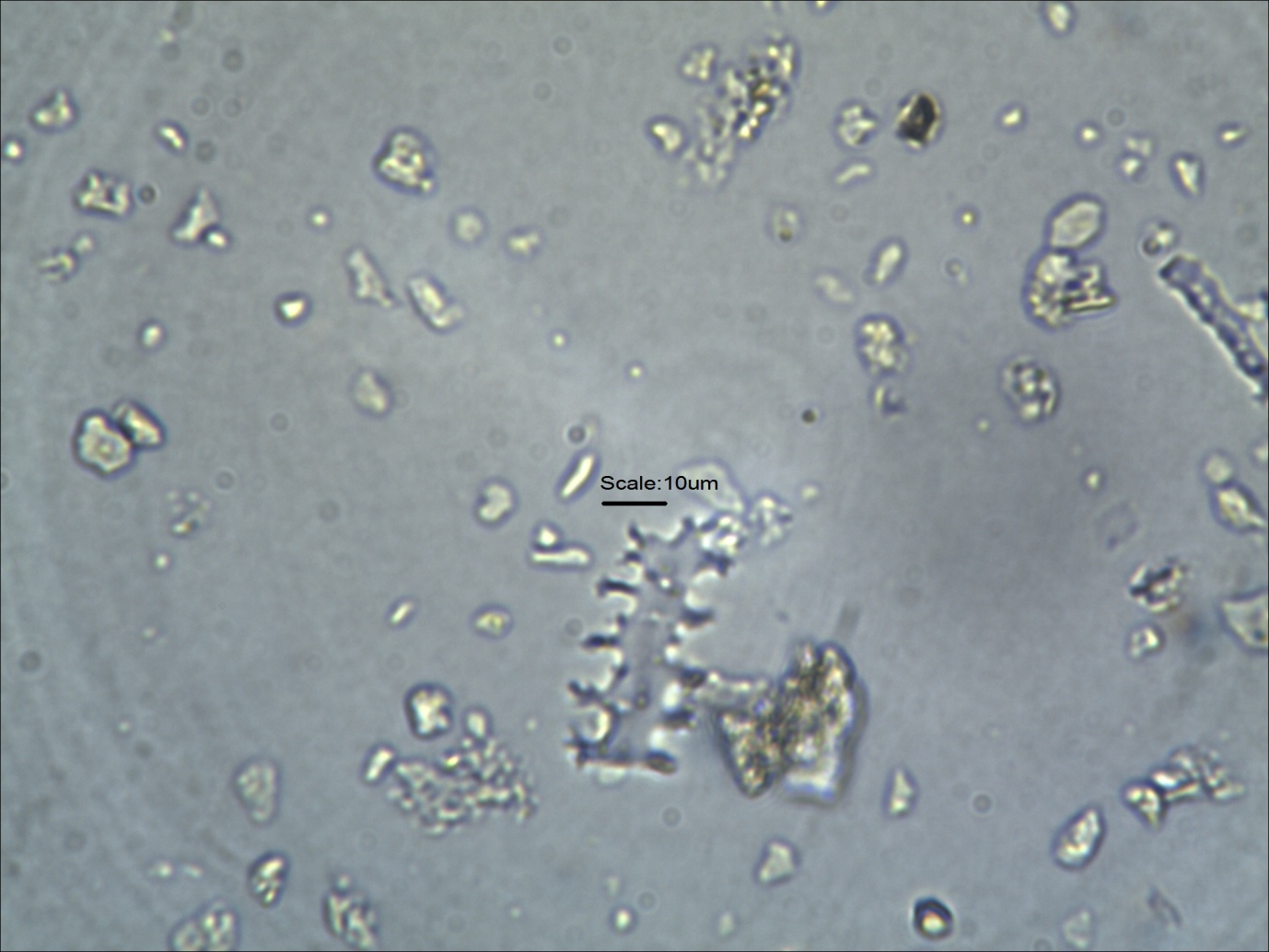 |
| --- | --- | --- |
| **Fig. S1** Elongate psilate | **Fig. S2** Elongate echinate wheat/barley type | **Fig. S3** Elongate dendritic wheat/barley type |
| 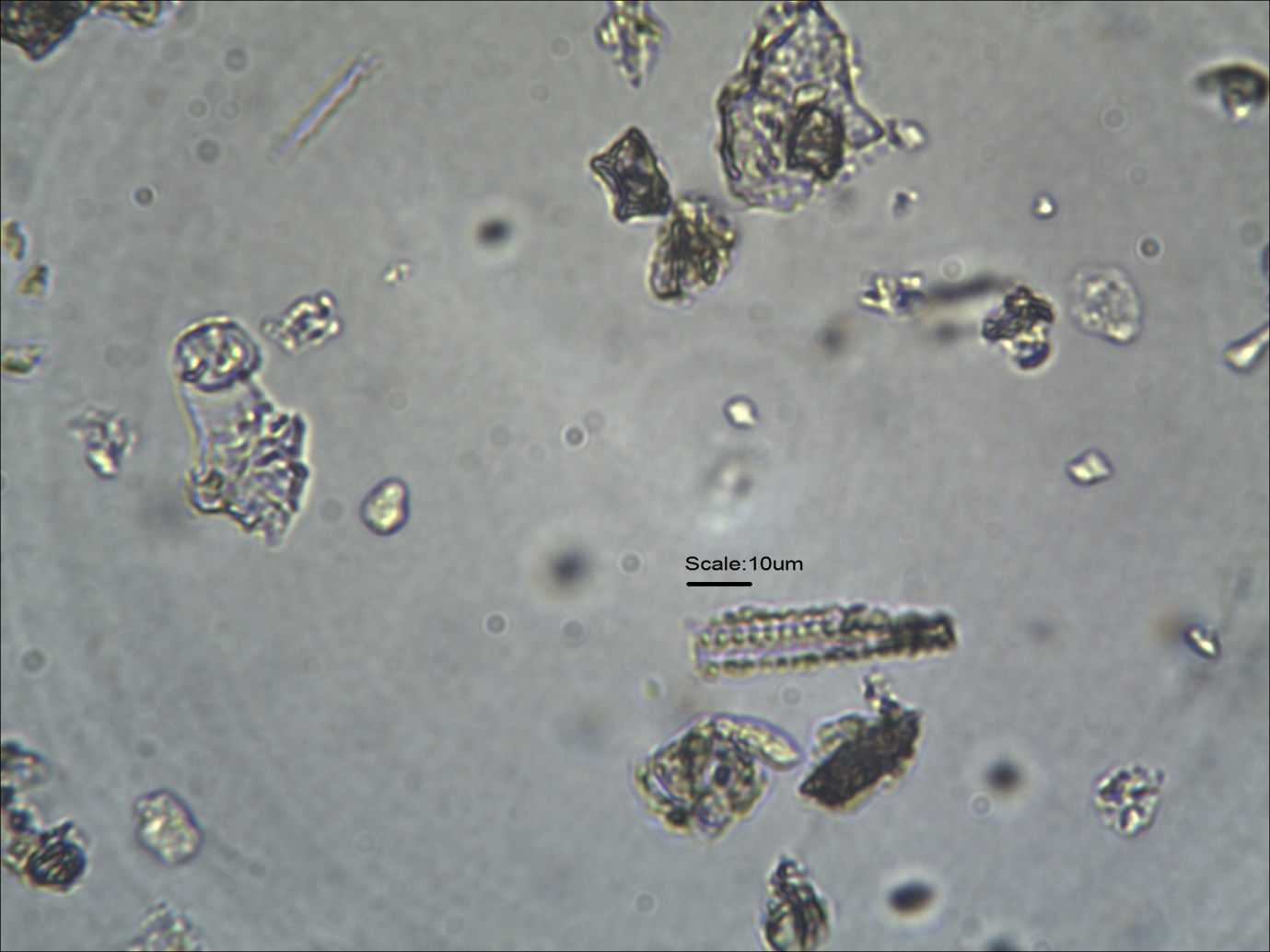 | 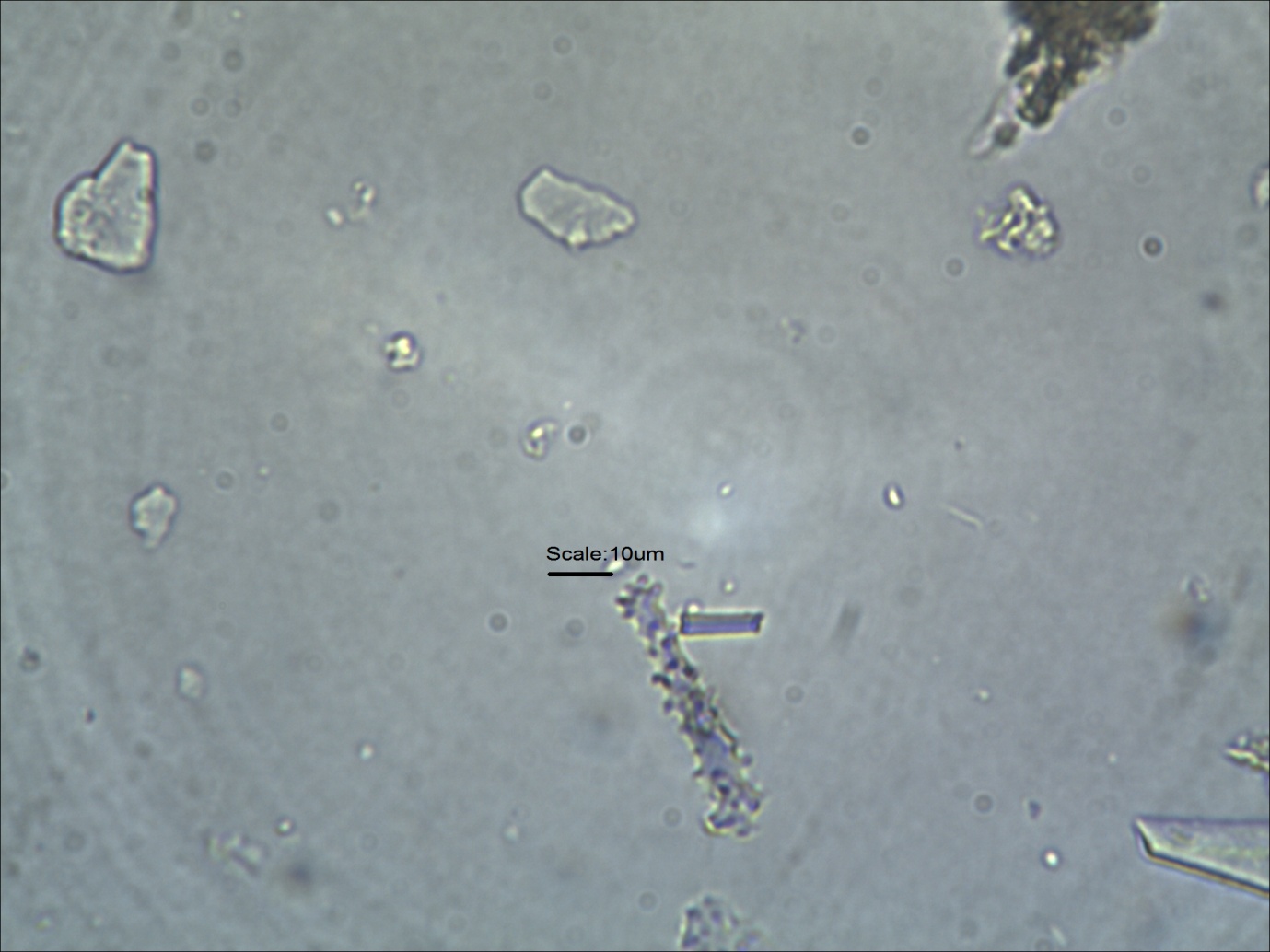 | 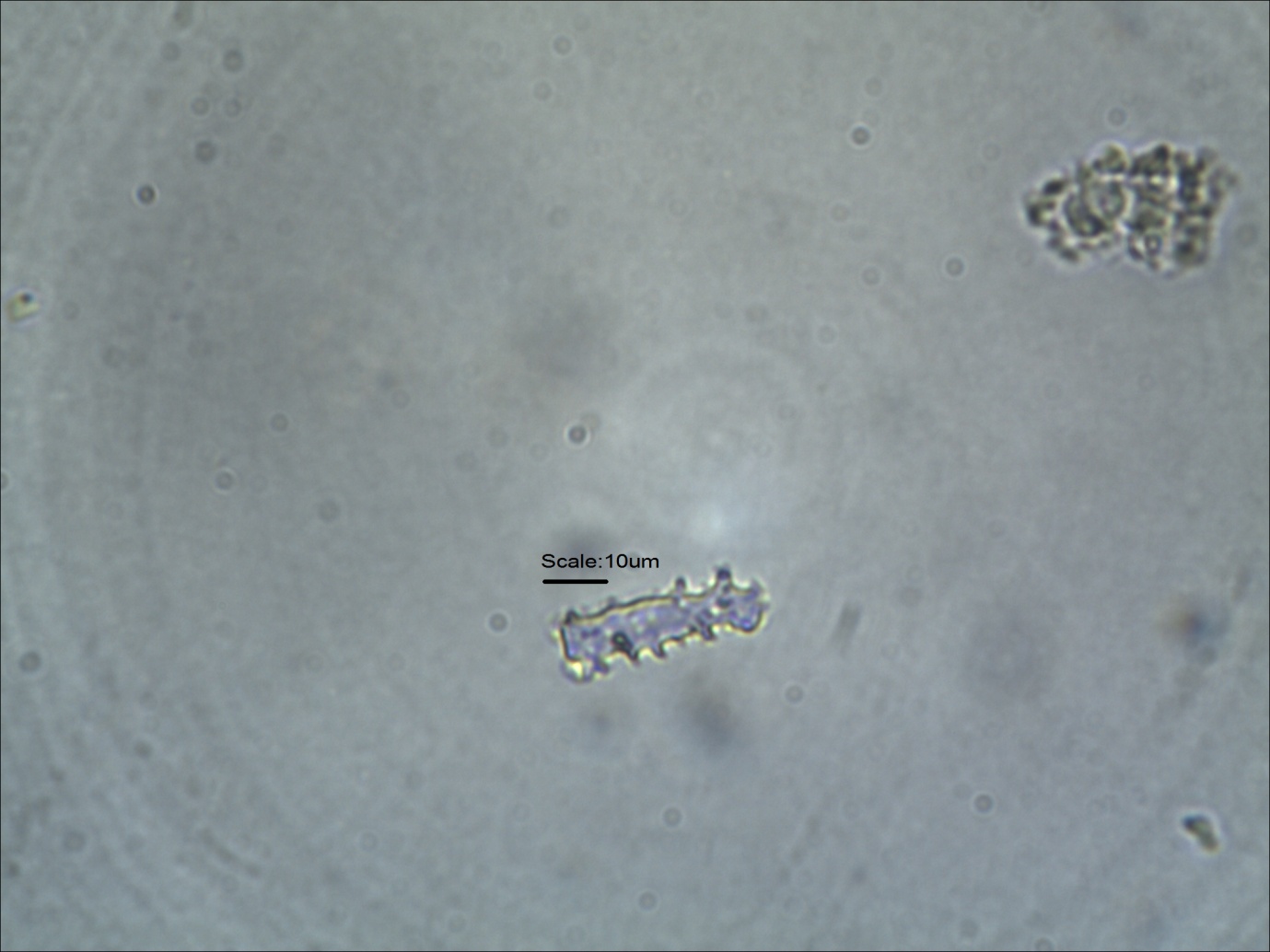 |
| **Fig. S4** Elongate echinate short spine | **Fig. S5** Elongate dendritic short spine | **Fig. S6** Elongate echinate short hook spine |
| *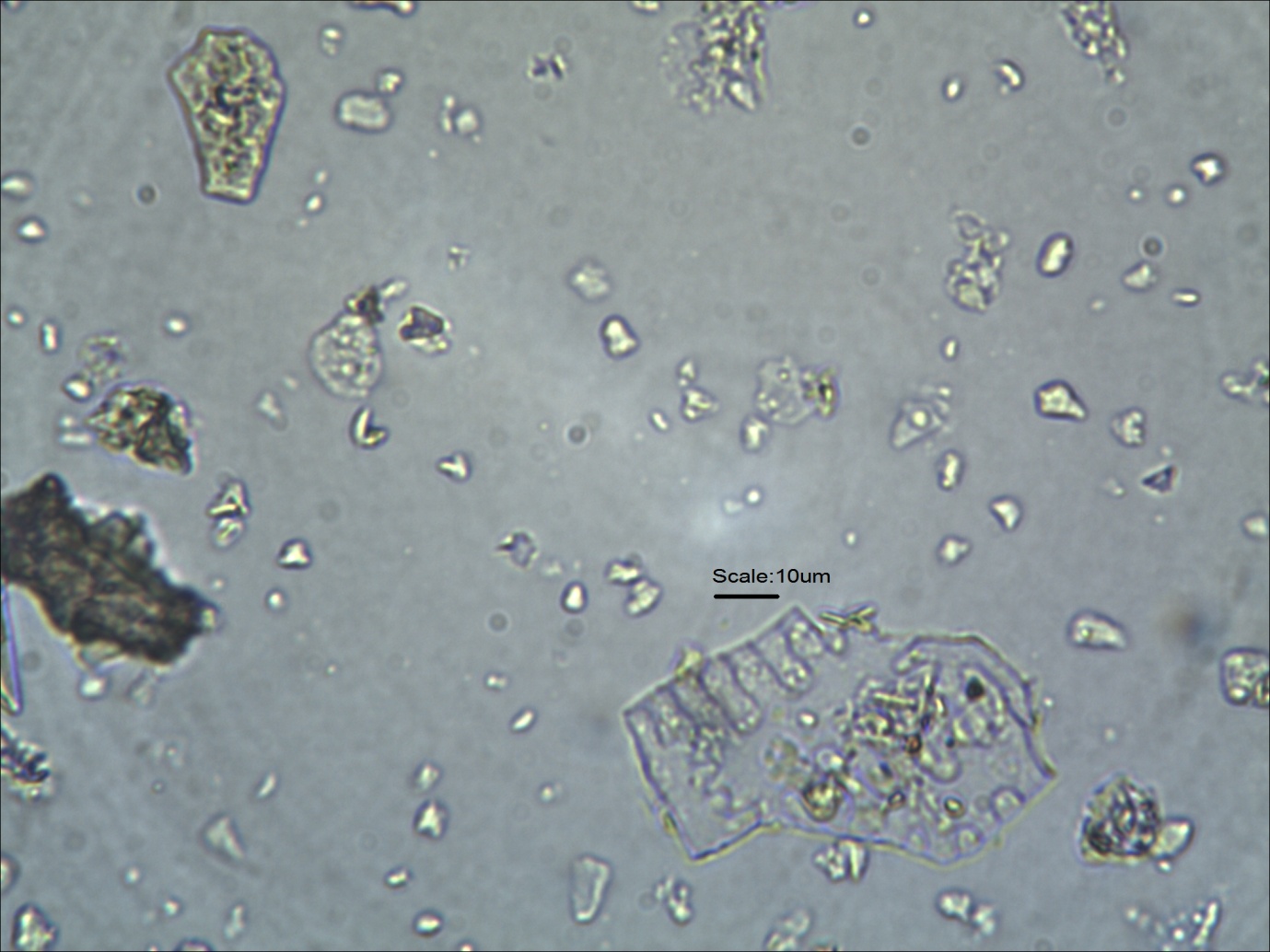* | *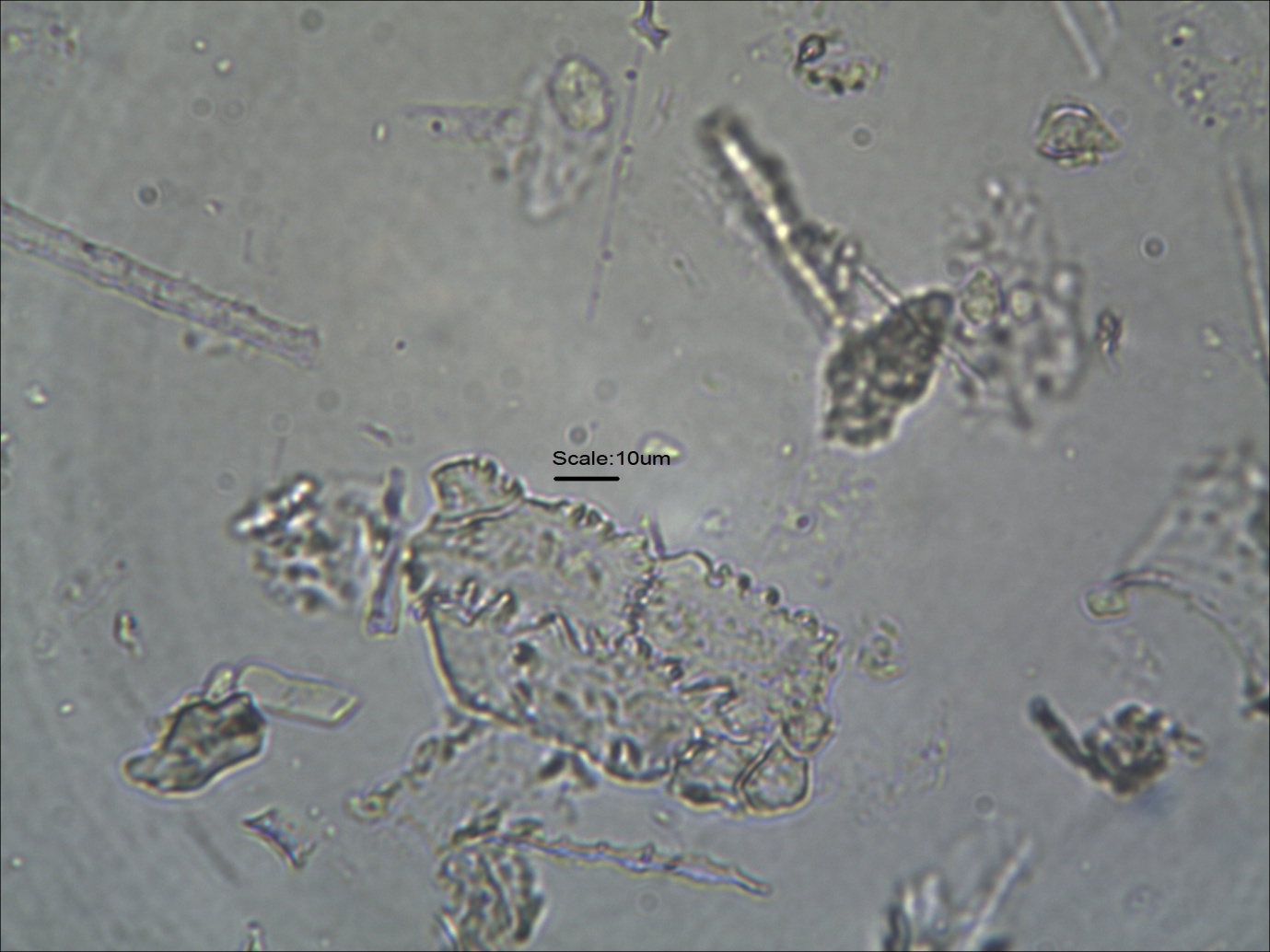* | *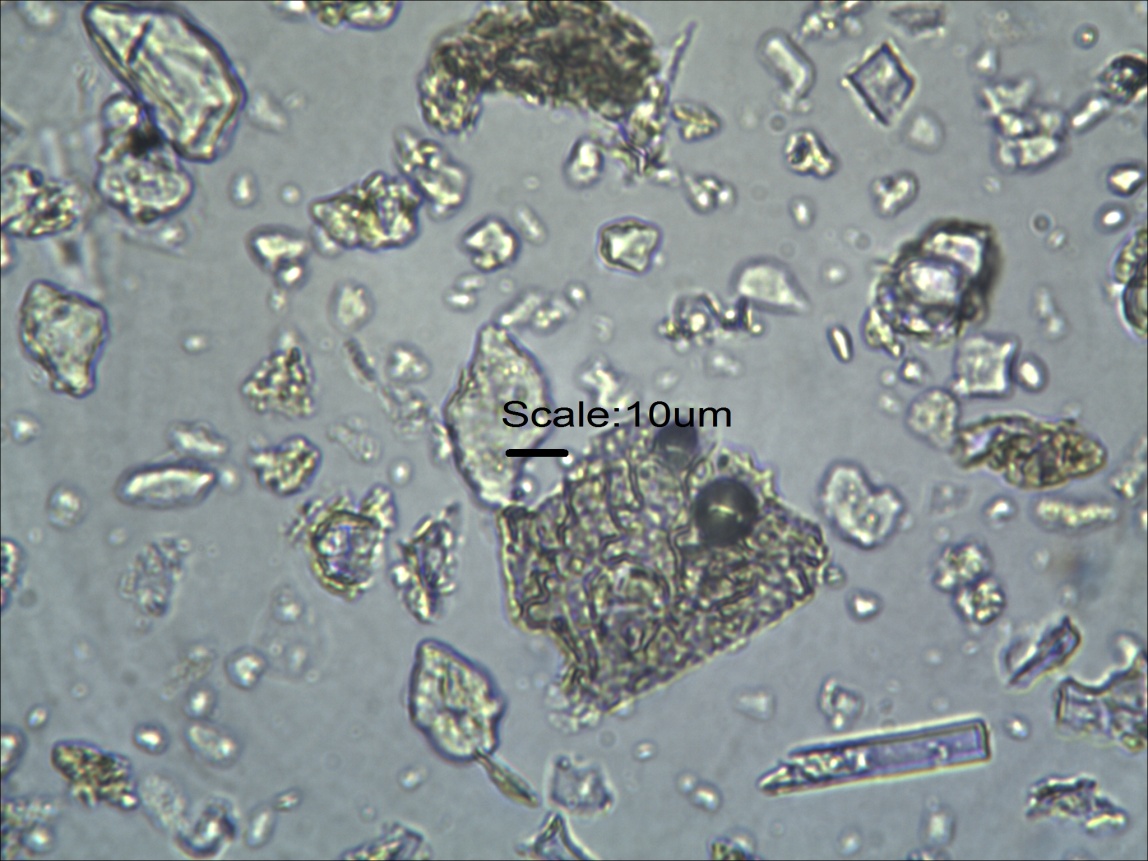* |
| **Fig. S7** Elongate wavy Echinochloa type (after Madella et al. 2013: Fig. 2) | **Fig. S8** Elongate wavy Brachiaria type (after Madella et al. 2013: Fig. 2) | **Fig. S9** Elongate wavy Sorghum type (after Madella et al. 2013: Fig. 3) |

| 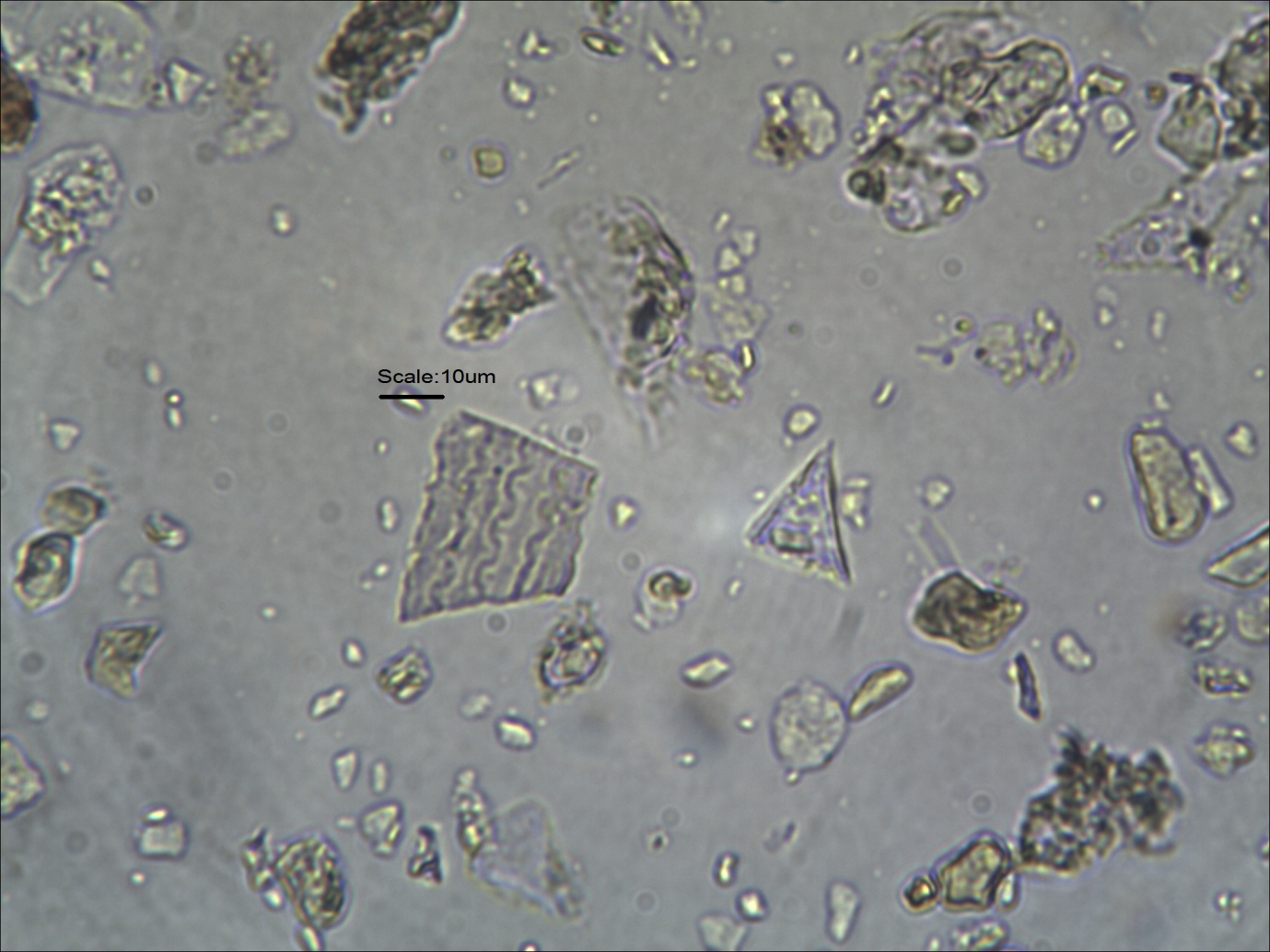 | 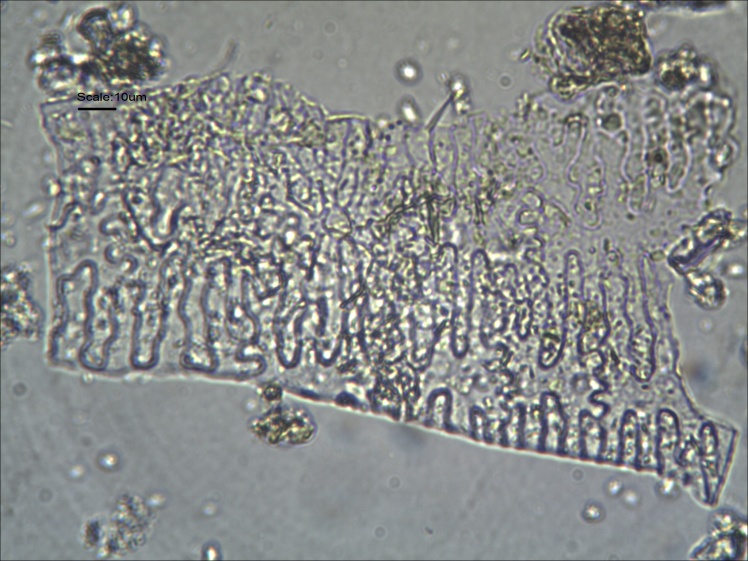 | 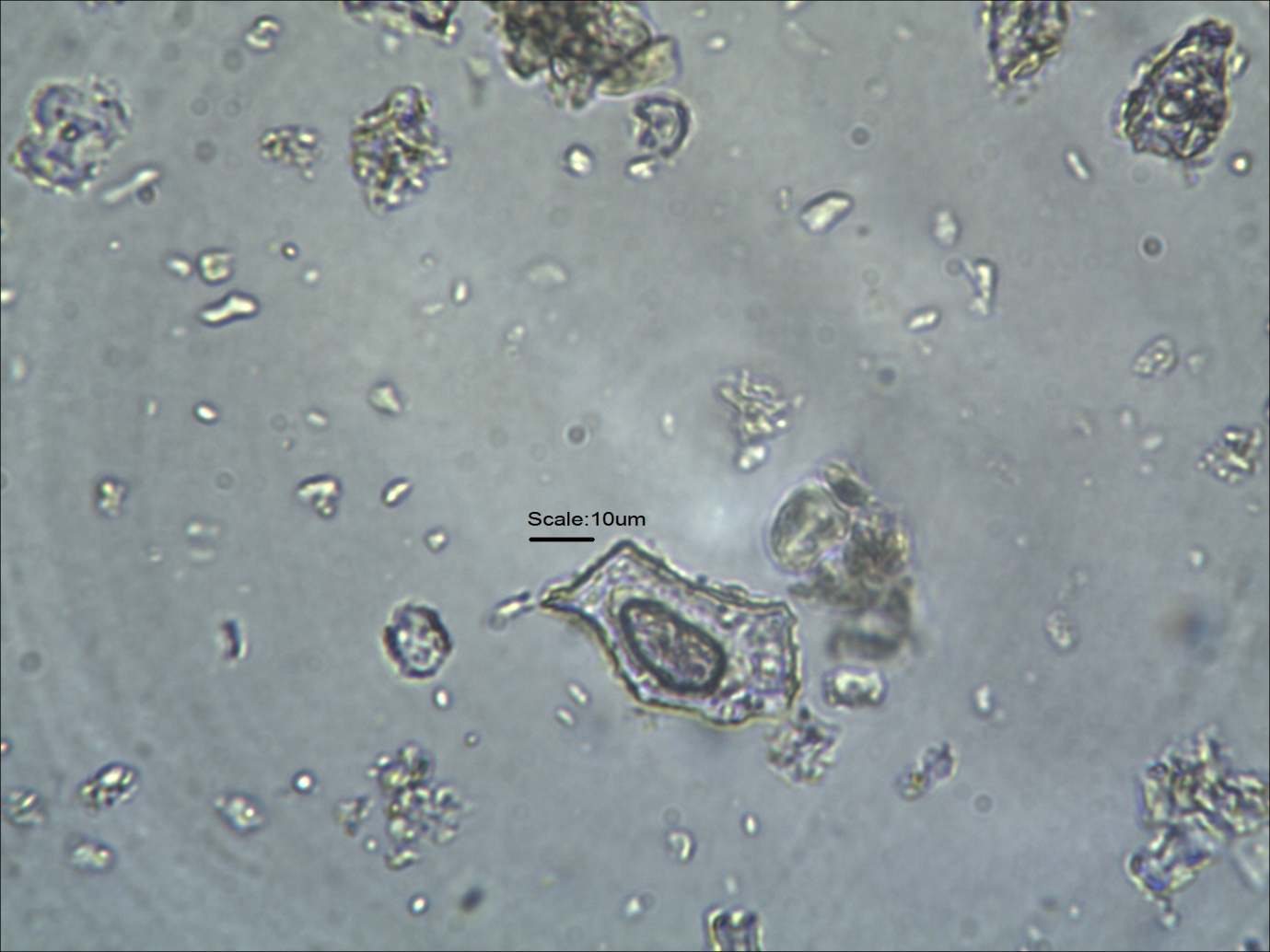 |
| --- | --- | --- |
| **Fig. S10** Elongate wavy Setaria cf. verticillata type (after Madella et al. 2013: Fig. 3) | **Fig. S11** Elongate wavy Panicum cf. miliaceum type (after Lu et al. 2010: Fig. 3, 5, 6, 8, 9, 12, 13) | **Fig. S12** Commelinaceae Type (after Eichhorn et al. 2010: Fig. 3:L) |
| 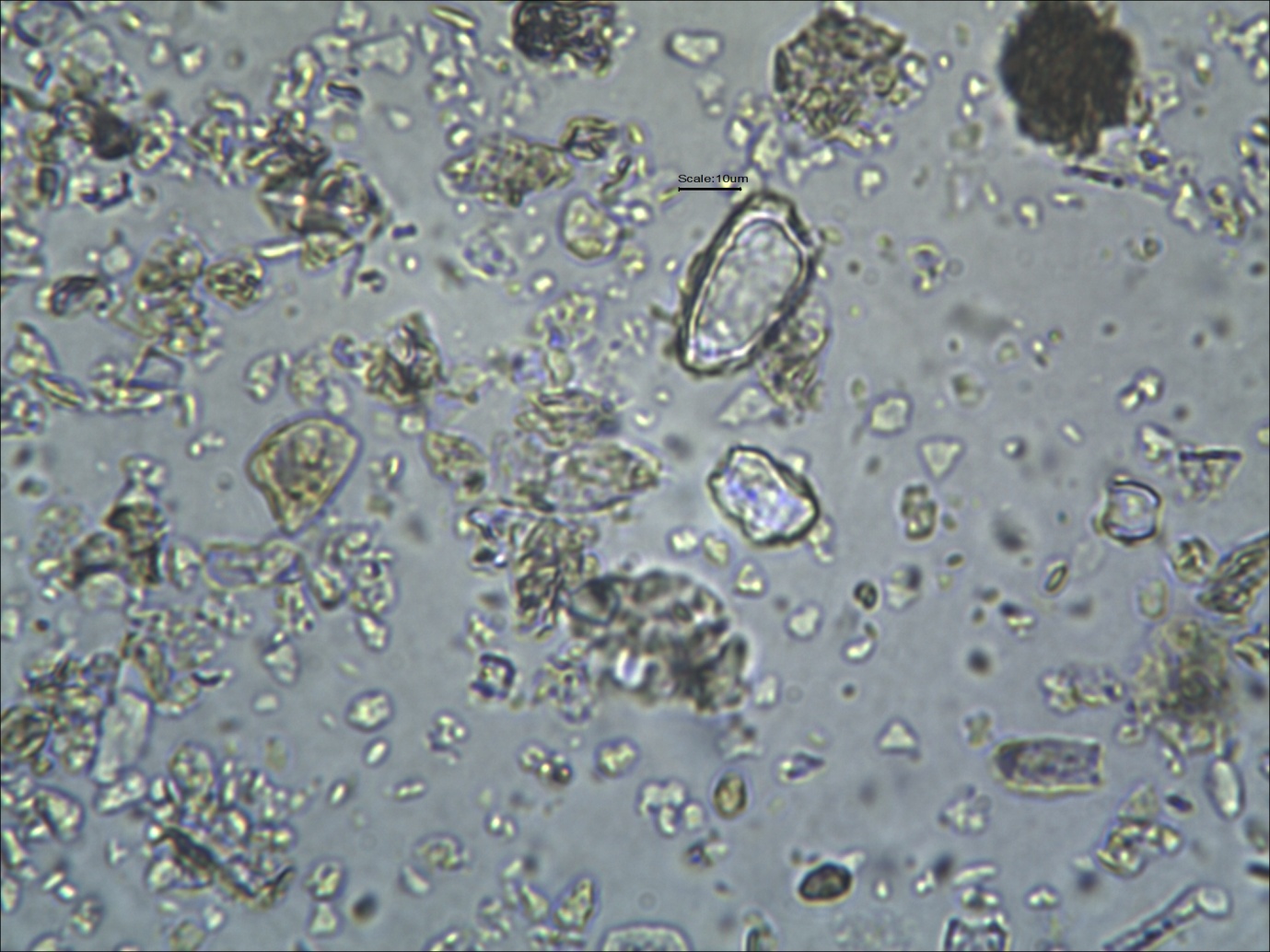 | 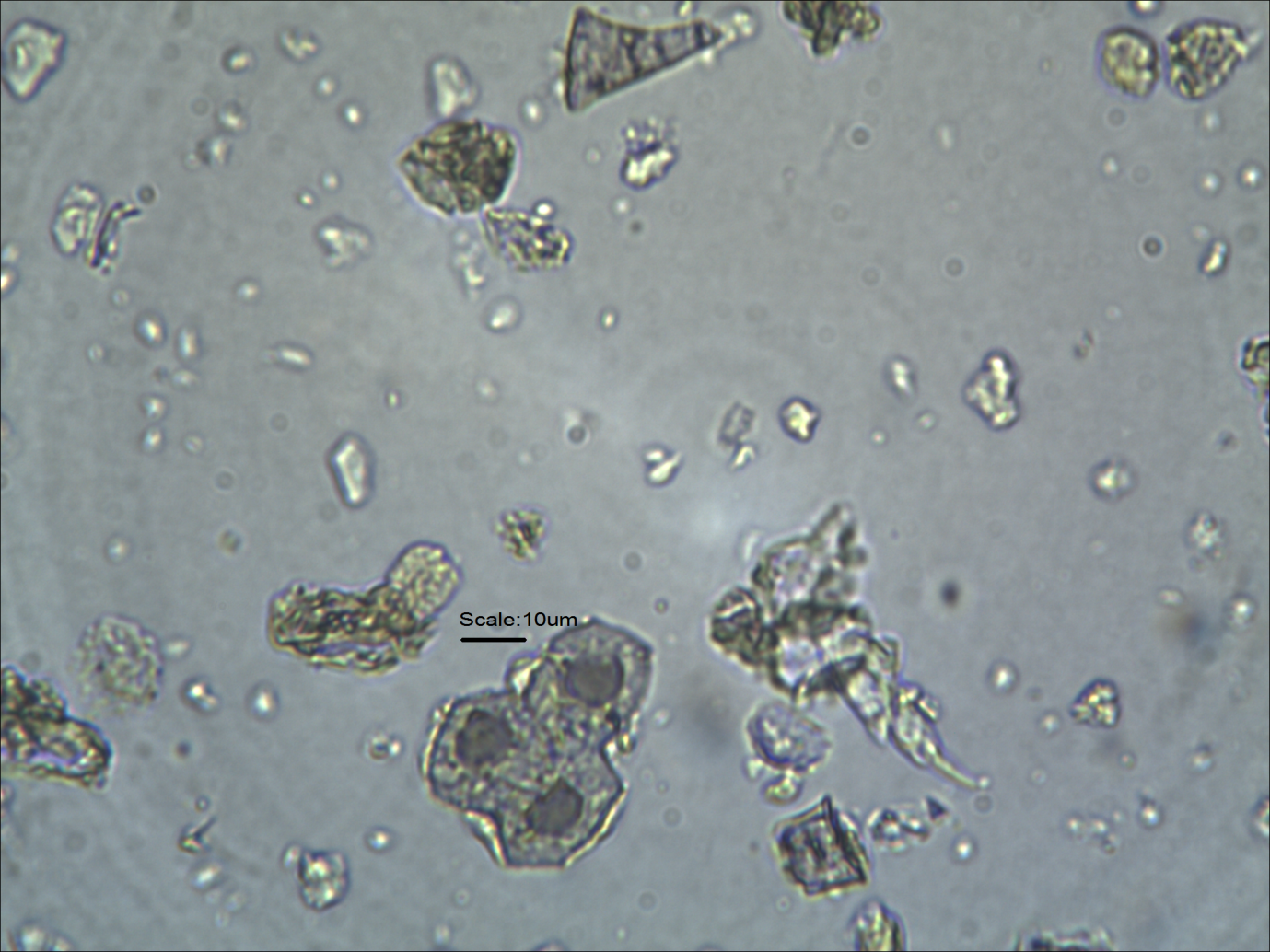 |  |
| **Fig. S13** Commelinaceae Type A (after Eichhorn et al. 2010: Fig. 3:A) | **Fig. S14** Commelinaceae Type I (after Eichhorn et al. 2010: Fig. 3:I) | |

Elongate wavy *Setaria* cf. *italica*-type (based on Lu et al. 2009: Fig. 3, 4, 5, 6, 7, 9, 11, 13) rarely found, un-photographed

Commelinaceae-type K (based on Eichhorn et al. 2010: Fig. 3:K) rarely found, un-photographed

References

Lu H, Zhang J, Wu N, Liu K, Xu D, Li Q (2009) Phytoliths analysis for the discrimination of foxtail millet (Setaria italica) and common millet (Panicum miliaceum). PLoS One 4:e4448. doi:10.1371/journal.pone.0004448

Madella M, Lancelotti C, Garc´ıa-Granero JJ (2013) Millet microre- mains. An alternative approach to understand cultivation and use of critical crops in prehistory. Archaeol Anthropol Sci. doi:10. 1007/s12520-013-0130-y
